# α-CH acidity of alkyl–B(C_6_F_5_)_2_ compounds – the role of stabilized borata-alkene formation in frustrated Lewis pair chemistry†
†Electronic supplementary information (ESI) available: Details of the DFT calculations and experimental details. CCDC 1006899–1006903. For ESI and crystallographic data in CIF or other electronic format see DOI: 10.1039/c4sc01711k



**DOI:** 10.1039/c4sc01711k

**Published:** 2014-09-26

**Authors:** Philip Moquist, Guo-Qiang Chen, Christian Mück-Lichtenfeld, Kathrin Bussmann, Constantin G. Daniliuc, Gerald Kehr, Gerhard Erker

**Affiliations:** a Organisch-Chemisches Institut , Universität Münster , Corrensstrasse 40 , 48149 Münster , Germany . Email: erker@uni-muenster.de

## Abstract

Alkyl–B(C_6_F_5_)_2_ boranes are markedly α-CH-acidic. Using DFT we have calculated the p*K*_a_-values of a series of examples. Typically, (C_6_F_5_)_2_B–CH_3_ [p*K*_a_ (calcd) = 18.3 in DMSO, 16.2 in dichloromethane] is almost as CH-acidic as cyclopentadiene. However, this α-CH–B(C_6_F_5_)_2_ acidity is in most cases not sufficient to allow for internal proton transfer in vicinal phosphane/borane frustrated Lewis pairs (FLPs). An exception is the slightly endergonic (+0.3 kcal mol^–1^) tautomerization of the *in situ* generated indane derived 1,3-P/B FLP **6** to its zwitterionic borata-alkene/phosphonium isomer **7**, which was successfully trapped by Piers' borane [HB(C_6_F_5_)_2_] to yield the stable product **8**. The pronounced α-CH[B] carbanion stabilization can be used synthetically. We have found that the dienyl borane *E*-H_2_C=C(Me)CH=CHB(C_6_F_5_)_2_ undergoes clean, thermally induced 1,4-hydrophosphination reactions with HPR_2_ (R: phenyl, mesityl, or *t*-butyl) reagents. α-CHB(C_6_F_5_)_2_ carbanion (*i.e.* borata-alkene) stabilization in the respective intermediates probably plays a decisive role in these reactions.

## Abstract 2

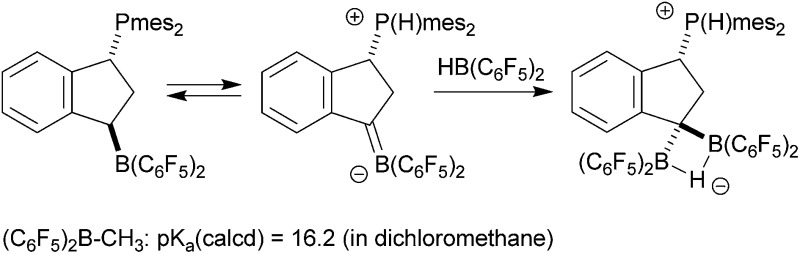
Alkyl–B(C_6_F_5_)_2_ boranes are markedly α-CH-acidic. Using DFT we have calculated the p*K*_a_-values of a series of examples. Typically, (C_6_F_5_)_2_B–CH_3_ [p*K*_a_ (calcd) = 18.3 in DMSO, 16.2 in dichloromethane] is almost as CH-acidic as cyclopentadiene.

## Introduction

**Scheme 1:**
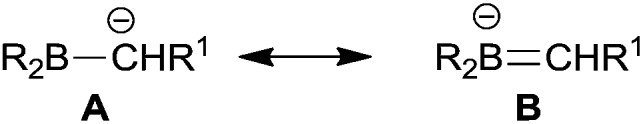


Electrophilic boranes stabilize carbanions in the α-position. The boryl carbanions (**A**) are mostly better described by their borata-alkene resonance forms (**B**) (see Scheme 1).

**Scheme 2:**
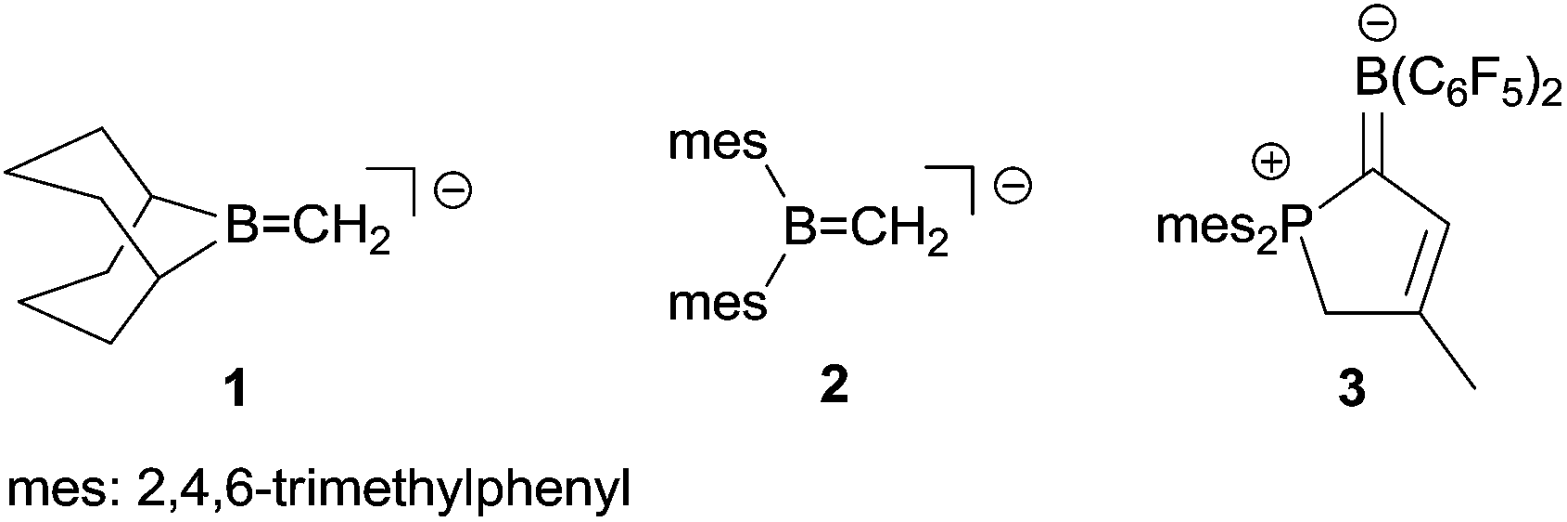


Despite the apparently significant possible stabilization of carbanions by adjacent boryl functional groups, there have not been too many isolated and well characterized examples of such borata-alkenes described so far.^2,3^ This is probably to a large extent due to the preferred competing addition of basic reagents to the borane Lewis acid over α-CH abstraction. Therefore, most of the borata-alkenes reported so far bear bulky substituents on boron to circumvent kinetically preferred boron Lewis acid/Lewis or Brønsted base adduct formation. Some typical examples (**1**,**2**) are shown in Scheme 2. In addition, we have recently reported the formation of zwitterionic borata-alkenes (**3** in Scheme 2) by a base-free pathway.^4^

Many reactive intramolecular frustrated Lewis pairs (FLPs)^5,6^ contain CH units α-positioned to Lewis acidic boryl moieties. Very often these contain pairs of strongly electron withdrawing C_6_F_5_ substituents. Therefore, it is a question of whether there may be situations of intramolecular abstraction by the phosphane or amine Lewis base of α-CHs adjacent to *e.g.* the B(C_6_F_5_)_2_ group and if FLP chemistry might, in special cases, be influenced by borata-alkene participation. Eventually this raises the question of relative acidities and basicities of the –CH_2_B(C_6_F_5_)_2_ group and the adjacent Lewis base. In assessing this problem, we found that there is surprisingly little known in detail about α-CH–boryl acidity, especially for the allegedly strongly carbanion-stabilizing B(C_6_F_5_)_2_ group, which is of relevance here.^1*c*^

We have now found a case where the phosphonium/borata-alkene tautomer of an internal P/B FLP was apparently trapped. For a detailed analysis of this situation we have then determined the p*K*_a_ values of a variety of boranes relevant to the FLP-CH acidity problem by a DFT study,^7^ and we have eventually applied our findings to carry out a small series of uncatalyzed hydrophosphination reactions of boryl-dienes, a reaction which potentially has borata-alkenes involved as the decisive reactive intermediates.

## Results and discussion

### Experimental study: generation and trapping of a phosphonium/borata-alkene system

**Scheme 3:**
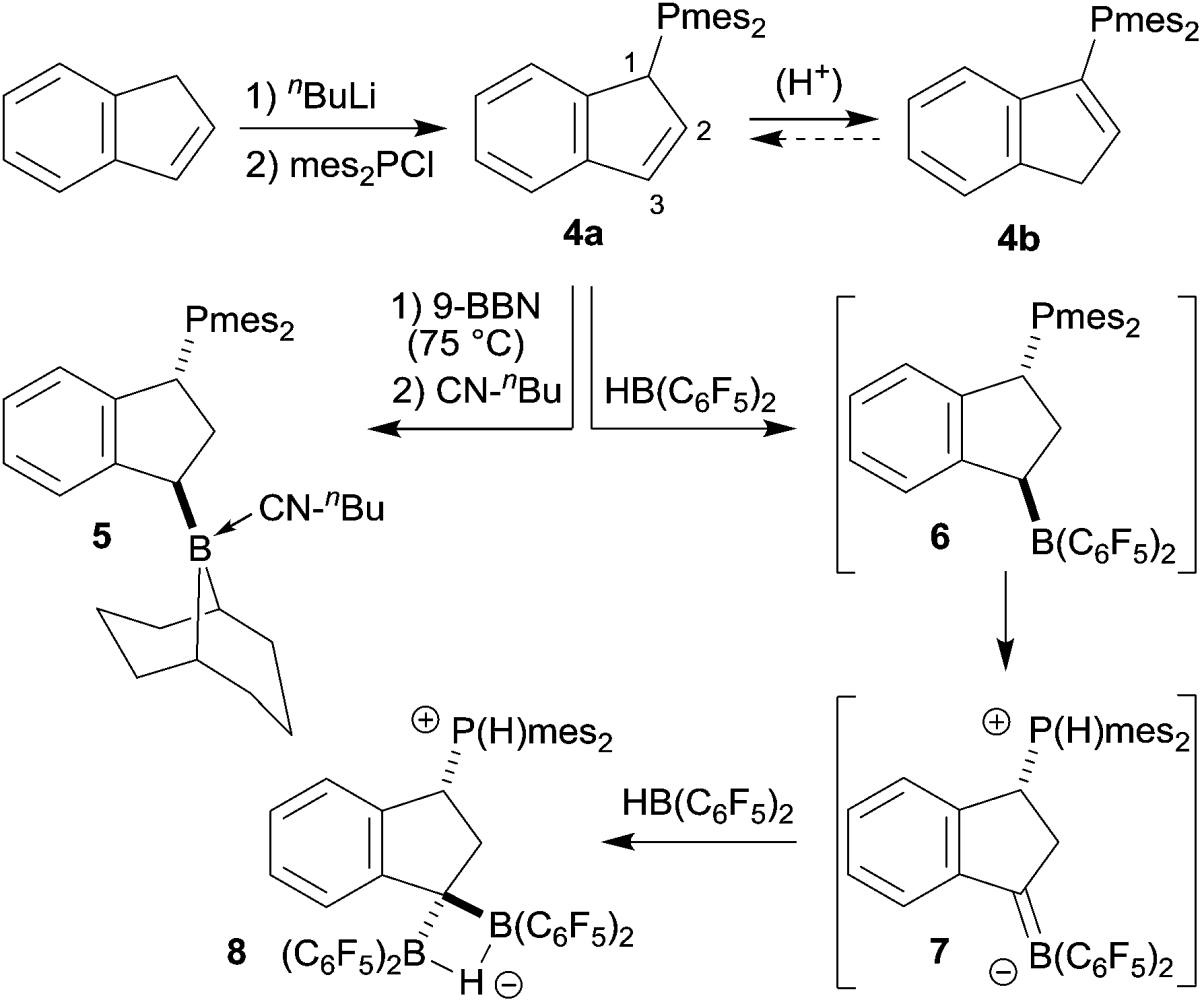


**Fig. 1:**
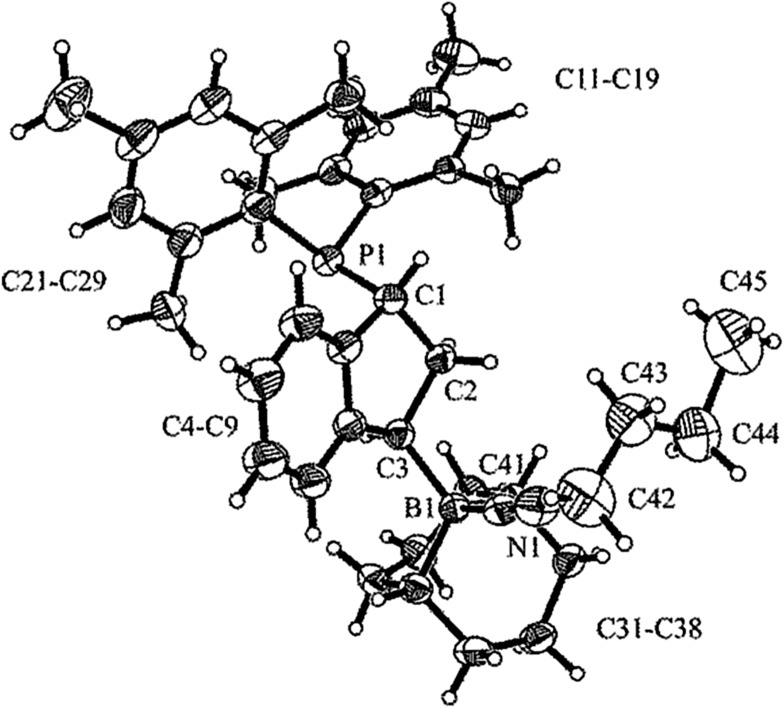
Molecular structure of compound **5** (thermal ellipsoids are shown with 30% probability). Selected bond lengths (Å) and angles (deg): P1–C1 1.864(3), C1–C2 1.549(3), C2–C3 1.549(3), C3–B1 1.649(4), B1–C41–N1 175.8(3), C41–N1–C42 176.8(3).

We prepared the dimesitylphosphino-indene system **4** by treatment of the *in situ* generated indenyllithium reagent with (mesityl)_2_PCl at 0 °C in ether. Direct workup under apparently acid free conditions gave phosphane **4a** as a white solid in 66% yield (see Scheme 3) [^31^P NMR: *δ* –14.5, ^1^H NMR: *δ* 6.97, 6.63 (3-H, 2-H), 5.27 (1-H)]. The compound is very easily isomerized to give the isomer **4b**. For example, this was achieved by stirring it with active alumina [^31^P NMR: *δ* –42.3, ^1^H NMR: *δ* 6.10 (2-H), *δ* 3.41 (CH_2_)] but isomerization took place invariably during workup. We reacted the indenyl phosphane isomer **4a** with 9-BBN to obtain a structural reference for the preferred regio- and relative stereochemistry of the hydroboration reaction of this system. The reaction of **4a** with the 9-BBN reagent was carried out in toluene at 75 °C (8 h). Then the resulting hydroboration product was directly converted to the isonitrile adduct **5** by treatment with *n*-butylisocyanide at r.t. (see Scheme 3). We isolated compound **5** as a white solid in 56% yield. It was characterized as the 1,3-disubstituted isomer by spectroscopy and by X-ray diffraction (see Fig. 1). In the crystal the bulky P(mesityl)_2_ substituent is found attached at the 1-position of the indane five-membered ring. The phosphorus coordination geometry is trigonal-pyrimidal with a sum of C–P–C angles ∑P1^CCC^ = 318.1°. The 9-BBN substituent is bonded to the distal carbon atom C3 at the same ring. The B1–C3 vector is *trans*-oriented to the P1–C1 vector. The boron atom has also attached to it the isonitrile donor ligand in a linear arrangement of the B1–C41–N1–C42 unit.

Compound **5** shows heteronuclear magnetic resonance signals at *δ* –17.7 (^31^P) and *δ* –13.7 (^11^B). The three carbon atoms of the five-membered carbocyclic subunit of the indane derivative **5** give rise to ^13^C resonances at *δ* 41.1 (C1, ^1^ *J*_PC_ = 19.4 Hz), 38.3 (C2, ^2^ *J*_PC_ = 23.4 Hz) and 34.4 (C3, br) with corresponding ^1^H NMR signals at *δ* 4.66 (1-H), 2.38, 2.18 (2-H), and 2.95 (3-H). The isonitrile carbon resonance of compound **5** occurs at *δ* 138.1.

We then reacted the phosphanes **4** with Piers' borane [HB(C_6_F_5_)_2_].^8^ The reaction of either of the indenyl phosphanes **4a** and **4b** eventually yielded the same product **8**. The reaction required two molar equivalents of the hydroboration reagent to go to completion under the applied reaction conditions (1 h, r.t.). Treatment of *e.g.* **4b** with only one molar equiv. of [HB(C_6_F_5_)_2_] under direct NMR monitoring gave *ca.* 0.4 equiv. of the product **8** and left most of the remaining starting material untouched.

On a preparative scale the **4a** + 2 [HB(C_6_F_5_)_2_] reaction was typically carried out in toluene. For workup the toluene solution was carefully layered with pentane and the mixture was kept at –35 °C for 2 days to give a precipitate of compound **8** (see Scheme 3). It was isolated in 56% yield and characterized by C, H elemental analysis and by spectroscopy. The analogous reaction of **4b** with two molar equiv. of [HB(C_6_F_5_)_2_] gave the product **8** in 60% yield. Compound **8** features a broad ^11^B NMR resonance at *δ* –16.2 and a typical ^31^P NMR doublet at *δ* –2.0 with a corresponding ^1^H NMR [P]H resonance at *δ* 7.62 (^1^ *J*_PH_ = 477.5 Hz). It shows ^13^C NMR resonances of the three saturated carbon centers of the indane five-membered ring at *δ* 40.1 (C1, ^1^ *J*_PH_ = 42.6 Hz), 38.7 (C2) and 28.4 (C3, br) with corresponding ^1^H NMR signals at *δ* 5.38 (1-H), 2.71 and 2.53 (2-H). The broad ^1^H NMR [B]–H–[B] signal was located at *δ* 3.30. The compound shows ^19^F NMR signals of four chemically different C_6_F_5_ substituents. There is at least hindered rotation around some of the B-C_6_F_5_ vectors, so that we have monitored a total of six *ortho*-fluorine ^19^F NMR signals (in a 1 : 1 : 2 : 2 : 1 : 1 ratio) and there are four equal intensity *para*-^19^F NMR signals of the C_6_F_5_ groups in compound **8**.

**Scheme 4:**
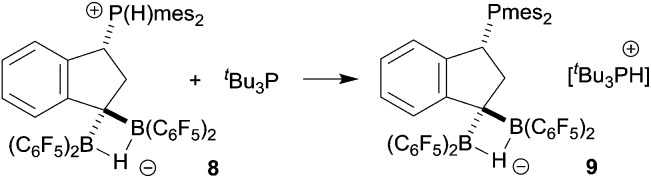


**Fig. 2:**
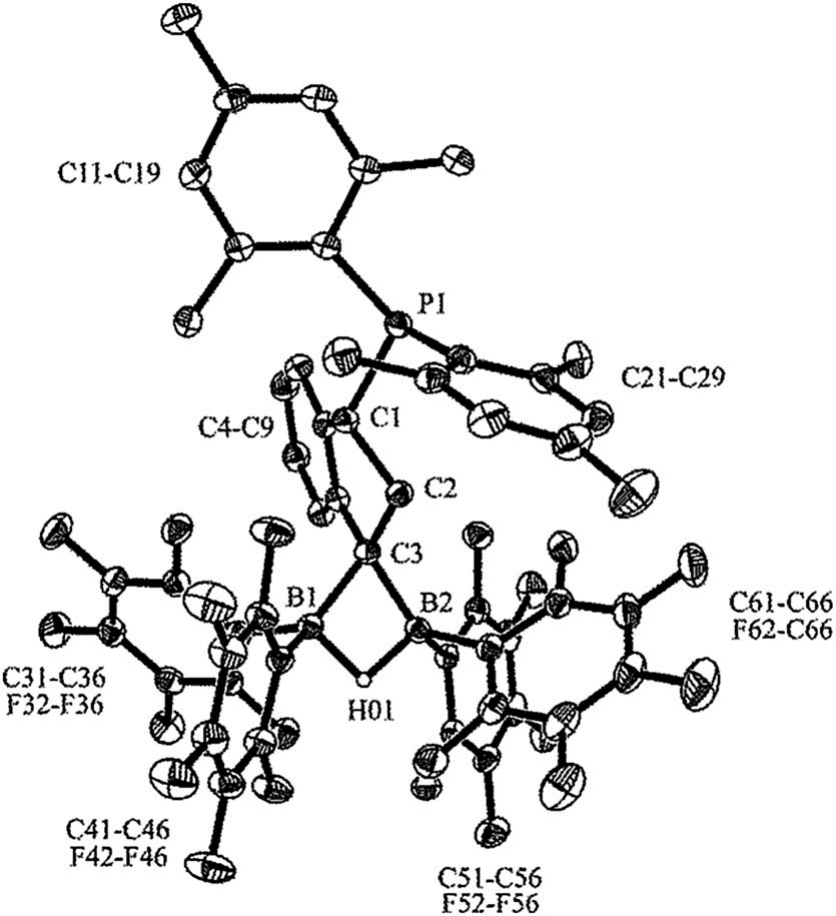
A view of the molecular structure of the anion of salt **9** (hydrogen atoms are omitted for clarity with the exception of H01; thermal ellipsoids are shown with 30% probability). Selected bond lengths (Å) and angles (deg): P1–C1 1.863(2), C1–C2 1.541(3), C2–C3 1.534(3), C3–B1 1.626(3), C3–B2 1.612(3), B1–H01 1.35(2), B2–H01 1.29(2), B1–C3–B2 73.9(2), P1–C1–C2–C3 –151.9(1), C1–C2–C3–B1 –100.1(2), C1–C2–C3–B2 172.5(2).

Since we could not obtain suitable crystals of **8** for the X-ray crystal structure analysis, we reacted it with the more basic ^*t*^Bu_3_P phosphane. This resulted in proton transfer and we obtained crystals of the salt **9** which were suitable for characterization by X-ray diffraction (see Scheme 4 and Fig. 2).

In the crystal, salt **9** shows the central indane framework of the respective anion. It features a slightly envelope shaped conformational arrangement of the annulated five-membered carbocyclic ring. This unit has the bulky (mesityl)_2_P substituent bonded to the carbon atom C1. Again, the phosphorus coordination geometry is trigonal-pyrimidal with a sum of C–P–C bond angles of ∑P1^CCC^ = 318.2°. Carbon atom C3 has a pair of B(C_6_F_5_)_2_ substituents bonded to it. The boron atoms themselves feature a bridging hydride.^9^ Consequently, the coordination geometry at each boron center is pseudo-tetrahedral. The boron atoms show sums of their respective C–B–C bond angles of ∑B1^CCC^ = 349.6° and ∑B2^CCC^ = 348.1°. There is a separate [HP^*t*^Bu_3_]^+^ cation (see Fig. 2).

Although we were not able to directly observe any intermediate on the way to **8**, we assume a reaction pathway as depicted in Scheme 3. It is likely that the hydroboration reaction of either of the isomers of **4** is reversible under the applied conditions^8*b*^ which eventually leads to the formation of the hydroboration product **6**. This then appears to undergo a proton transfer reaction (either intra- or intermolecularly) to generate the borata-alkene/phosphonium zwitterion system **7**. This contains the resonance form of an α-boryl stabilized carbanion. However, this system is apparently not persistent under our typical reaction conditions. It serves as a reactive carbon nucleophile that preferentially reacts with the [HB(C_6_F_5_)_2_] reagent present in the reaction mixture to rapidly give the observed product **8**.

### Assessment of α-CH–boryl acidity, a DFT study

The above described reaction represents a rare example of an intramolecular frustrated P/B Lewis pair undergoing proton transfer with the formation of a borata-alkene moiety (*i.e.* an α-boryl stabilized carbanion). This posed the general question of the stabilization of carbanions in the α-position to a boryl substituent. Since this had apparently not received adequate attention we decided to investigate and determine the carbanion stabilization features of a variety of α-boryl stabilized carbanions by computational methods.^7^ We calculated with density functional theory (TPSS-D3/def2-TZVP) the CH acidities of a series of boranes containing α-CH groups together with a few hydrocarbon systems used as references. The systems and the results of our DFT study are listed in Table 1. We calculated the p*K*_a_ values of these systems in several solvents. We have listed the p*K*_a_ values in dichloromethane and in DMSO in the table, some additional values can be found in the Supporting Information.†

**Table 1:** DFT-calculated p*K*_a_ values and Gibbs energies Δ*G*_rel_ of deprotonation of R–CH_2_–boryl and other compounds with the cyclopentadienyl anion Cp^–^

Entry	X–H	p*K*_a_			Δ*G*_rel_ (298 K)^*a*^ [kcal mol^–1^]		
		Vacuum	CH_2_Cl_2_	DMSO	Vacuum	CH_2_Cl_2_	DMSO
1	C_5_H_9_–H	61.1	60.7	60.4 (exp: 58)^*b*^	+58.7	+58.2	+57.7
2	PhCH_2_–H	37.4	41.1	41.7 (exp: 41)^*c*^	+26.4	+31.5	+32.2
3	9-BBN–CH_2_–H	26.7	32.4	33.1	+11.9	+19.6	+20.5
4	Mes_2_B–CH_2_–H	16.6	27.8	29.2	–1.9	+13.3	+11.2
5	(C_6_F_5_)_2_B–CH_2_–H	1.8	16.2	18.3	–22.0	–2.4	+0.5
6	C_5_H_5_–H(Cp–H)	**18.0** ^*d*^	**18.0** ^*d*^	**18.0** ^*d*^	**0.0**	**0.0**	**0.0**
7	(C_6_F_5_)_2_B–C(CH_3_)_2_–H	–2.9	13.0	15.4	–28.4	–6.8	–3.6
8	(C_6_F_5_)_2_B–CH(Ph)–H	–6.8	9.3	11.6	–33.8	–11.9	–8.7
9	(C_6_F_5_)_2_B–CH(CH=CH_2_)–H	–8.6	6.7	9.0	–36.3	–15.4	–11.3
10	Mes_2_EtP^+^–H	–61.1	–2.5	7.0	–106.6	–27.9	–15.0

^*a*^Calculated Gibbs energy Δ*G*_rel_ (TPSS-D3/def2-TZVP + COSMO) of the reaction X–H + Cp^–^ → Cp–H + X^–^.

^*b*^Extrapolated, see ref. 10.

^*c*^ Ref. 11a.

^*d*^Experimental p*K*_a_ of Cp–H in DMSO (ref. 10), taken here as the point of reference.

**Scheme 5:**
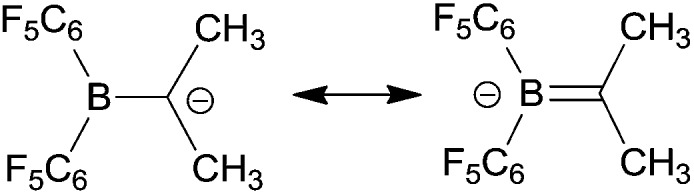


It becomes apparent from the values listed in Table 1 that the boryl groups are very substantially acidifying α-boryl alkanes thermodynamically. They stabilize the α-boryl-carbanions markedly. Thus, methyl-9-BBN (entry 3) is, by *ca.* 27 p*K*_a_ units, more acidic than *e.g.* cyclopentane (in DMSO). It is still *ca.* 8 p*K*_a_ units more acidic than toluene.^12^ The introduction of a pair of aryl groups at boron (here mesityl groups, see entry 4) has a measurable thermodynamic effect.^13^ As expected, the B(C_6_F_5_)_2_ group stabilizes the α-carbanion formation strongly. (C_6_F_5_)_2_B–CH_3_ has a (calculated) p*K*_a_ of *ca.* 18 in DMSO (*ca.* 16 in dichloromethane), which makes this borane almost as acidic as cyclopentadiene (entry 6). Alkyl substituents at the boryl α-carbanions exhibit an amazing effect in this chemistry. Usually, alkyl groups destabilize carbanions due to their electron-donating inductive effect. We have found here (by DFT) that the attachment of methyl groups on the boryl–CH system has an opposite effect: here the p*K*_a_ is lowered by *ca.* 3 p*K*_a_ units (entry 7). We assume that this is due to the high borata-alkene character of the boryl carbanions (see Scheme 5). The shortened B–C bonds of the boryl carbanions (*ca.* 1.45 Å *vs.* 1.57 Å in the α-boryl alkanes, see the ESI†) are additional evidence for this notion. These systems seem to almost behave as heteroalkenes and thus become more stabilized by increased substitution. Phenyl or vinyl substitution lead to a further stabilization of α-boryl carbanions as expected (entries 8 and 9).

To explain the relative acidities reported in Table 1, we have looked at electronic (charge stabilizing) factors which could determine the relative stabilities of the carbon bases. Table 2 correlates the charge distribution (obtained from a Natural Bond Orbital population analysis) of the anions with the acidities (p*K*_a_ values in CH_2_Cl_2_) of the corresponding CH_3_ acids. Apparently, there is no correlation of the p*K*_a_ with the charge of the CH_2_ group. However, if the boron atom is considered as a part of the anionic group, the negative charge is increasingly delocalized in the order Ph < 9-BBN < (Mes)_2_B < (C_6_F_5_)_2_B which is consistent with the calculated acidities. This supports the notion of [R_2_B=CH_2_]^–^ as a borata-alkene which has transferred most of its charge into the R substituents. Inspection of the HOMO of the anions confirms the borata-alkene character of the α-boryl carbanions (see the ESI†).

**Table 2:** Calculated (TPSS-D3/def2-TZVP) natural charges^*a*^ of the anionic CH_2_ groups and the boron atom in some anions of carbon acids R–CH_3_. Gibbs energy Δ*G*_solv_ of solvation of anions and neutral CH acids were obtained with COSMO

(R–CH_2_^–^) R =	*q*(CH_2_)	*q*(B)	*q*(B=CH_2_)	Calc. p*K*_a_ (CH_2_Cl_2_)	Δ*G*_solv_ (anion, CH_2_Cl_2_) [kcal mol^–1^]	Δ*G*_solv_ (acid, CH_2_Cl_2_) [kcal mol^–1^]
(C_6_F_5_)_2_B	–0.407	+0.268	–0.139	16.2	–33.4	–3.4
(Mes)_2_B	–0.530	+0.343	–0.187	27.8	–38.1	–3.7
9-BBN	–0.649	+0.422	–0.227	32.4	–42.7	–0.8
Ph	–0.367	—	(–0.367)	41.1	–46.9	–2.3

^*a*^See ref. 7g.

The better delocalization of the charge of *e.g.* (C_6_F_5_)_2_BCH_2_^–^ *vs.* PhCH_2_^–^ can also be concluded from the reduced Gibbs energy of solvation Δ*G*_solv_ (15.5 kcal mol^–1^ more for the latter, as calculated with the COSMO model). The solvation energy of the neutral carbon acids is comparably low for all examples in Table 2 (<5 kcal mol^–1^) and does not have a significant impact on the acidity.

Concerning the question of the Brønsted acid behavior in the P/B frustrated Lewis pairs (see above), the relative basicities of the α-boryl stabilized carbanion systems must be compared with the p*K*_a_ values of the respective phosphonium cations^13^ [here *e.g.* (mesityl)_2_EtPH^+^, see entry 10] to assess whether α-deprotonation of the boryl group might become a decisive factor in the respective intramolecular phosphine/borane frustrated Lewis pair chemistry. We see from the values listed in Table 1 that (C_6_F_5_)_2_B–CH_2_R systems that are devoid of any additional carbanion stabilizing groups are too low in acidity to become deprotonated by a phosphane in an intermolecular reaction (p*K*_a_ differences of *ca.* 10). The benzylic or allylic (C_6_F_5_)_2_B-borane systems come closer, but even here the p*K*_a_ difference seems in many cases just slightly insufficient to substantially populate the intermolecular borata-alkene/phosphonium salt alternative.

**Scheme 6:**
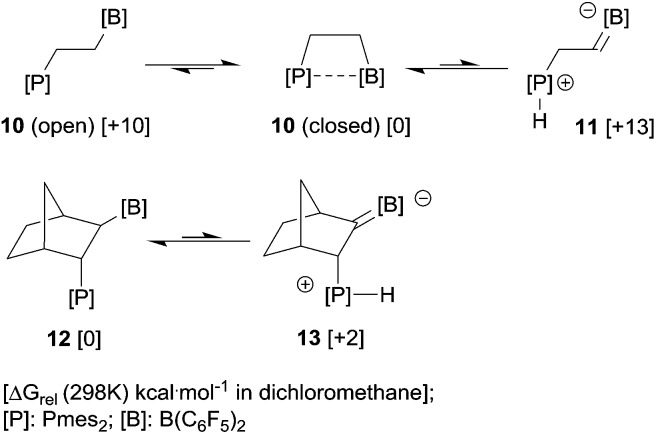


However, intramolecular cases might be different. In the relevant intramolecular frustrated P/B Lewis pair chemistry (see the examples depicted in Scheme 6) our DFT calculations have indicated the occurrence of significant energetic compensation effects due to the presence of both the α-boryl stabilized carbanion and the phosphonium cation at closely adjacent sites in the dipolar system. This compensation effect is substantial in solution and it leads to reduced energy differences between the neutral and polar tautomers of these intramolecular P/B FLP systems.

The vicinal FLPs **10** and **12** may serve as typical examples. The relative Gibbs energies of the zwitterions **11** and **13** when compared to the neutral tautomers **10** (open) and **12** are significantly lower than one would expect from the addition of the according Δ*G* values in Table 1. The close proximity of opposite charges in the zwitterions stabilizes the product of the proton transfer (the R_2_^(–)^B=CHCH_2_P^(+)^H fragment) compared to the formation of separated ions.

We had shown that this very reactive FLP exists in a closed form that shows a weak P–B interaction.^14,15^ In agreement with previous DFT calculations, the open form of **10** is about 10 kcal mol^–1^ higher in energy.^14,16^ This present study has now shown that the zwitterionic isomer **11** formally obtained by intramolecular α-CH–[B] deprotonation by the adjacent phosphane base is endergonic by +13 kcal mol^–1^, which is less than was expected from the energies and p*K*_a_ values of the isolated borata-alkene and phosphonium moieties listed in Table 1. Nevertheless, from these values the direct involvement of the tautomer **11** in the chemistry of the FLP **10** is unlikely, and so far we have not found any indication of its involvement in the typical chemistry observed for this reactive FLP system.

We had recently described the synthesis and chemistry of the FLP **12** and shown that this system is free of any significant intramolecular P/B interaction.^17^ In this case the zwitterionic isomer **13** containing the α-C–B(C_6_F_5_)_2_ carbanion located close to the phosphonium moiety was calculated to be higher by *ca.* 2 kcal mol^–1^ than the original P/B FLP tautomer. This is in accord with experiments where we have so far not observed the zwitterionic isomer under equilibrium conditions. We had also treated both the systems **10** and **12** with excess [HB(C_6_F_5_)_2_]. They both reacted very slowly and in both cases the products did not contain any phosphonium [P]–H^+^ moieties. We conclude that in these two representative cases the zwitterionic borata-alkene/phosphonium isomers are energetically located above their uncharged “normal” FLP isomers, but their energetic separation is such that it might be envisioned to eventually see them involved in their chemistry, although so far for these two typical systems this has not as yet been found experimentally in contrast to the above described indane derived system **8** (see Scheme 3).

**Scheme 7:**
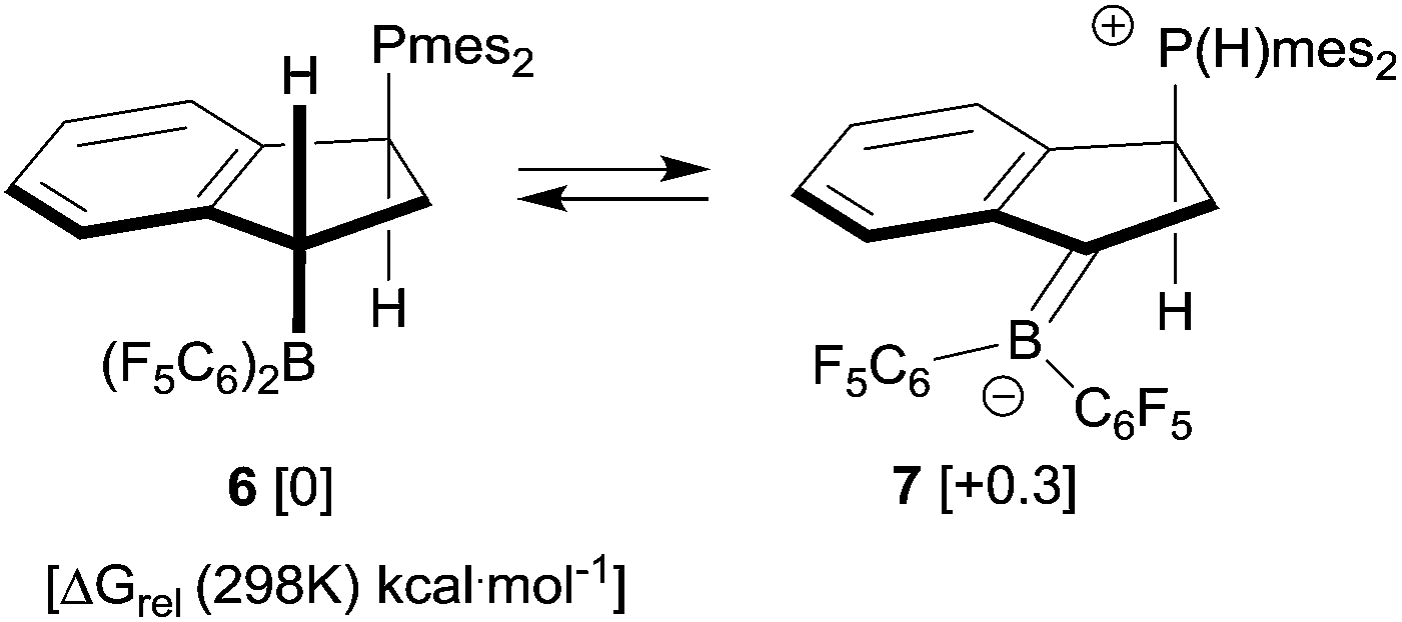


**Fig. 3:**
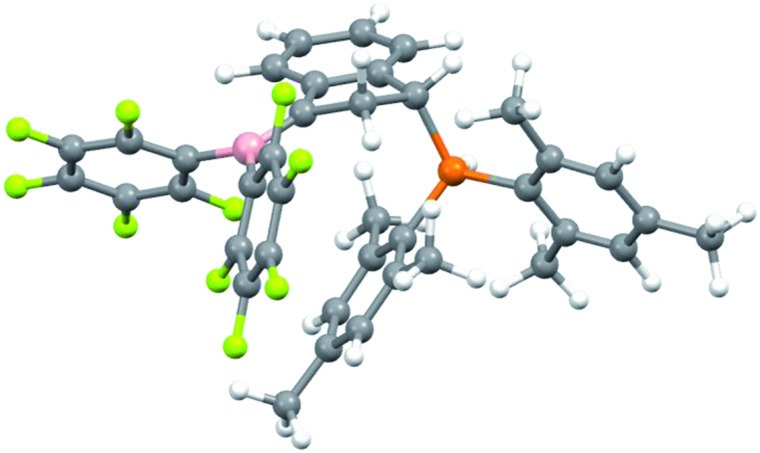
Calculated (DFT) structure of the zwitterionic compound **7**.

For that specific system our DFT study has located the zwitterionic tautomer **7** as being only 0.3 kcal mol^–1^ above the uncharged FLP **6** (in dichloromethane solution, see Scheme 7). In this case benzylic stabilization may have helped to further reduce the energy difference between the two tautomers. The calculated small energy difference between **6** and **7** makes it likely that compound **7** is indeed involved as a reactive intermediate in the formation of the eventually observed [HB(C_6_F_5_)_2_] trapping product **8** (see Scheme 3). Fig. 3 shows a view of the calculated DFT structure of the phosphonium/borata-alkene zwitterion **7**.

### Uncatalyzed hydrophosphination of a conjugated boryldiene – an application

Hydroamination^18,19^ and the related hydrophosphination are important reactions for synthesizing amines and phosphanes, respectively. Hydroaminations are mostly metal catalyzed^19^ although recently a main group (frustrated Lewis pair) induced alternative has been described.^20^ Hydrophosphinations can be performed by a radical induced reaction pathway,^21^ although hydrophosphinations are also often metal catalyzed.^22^

**Scheme 8:**
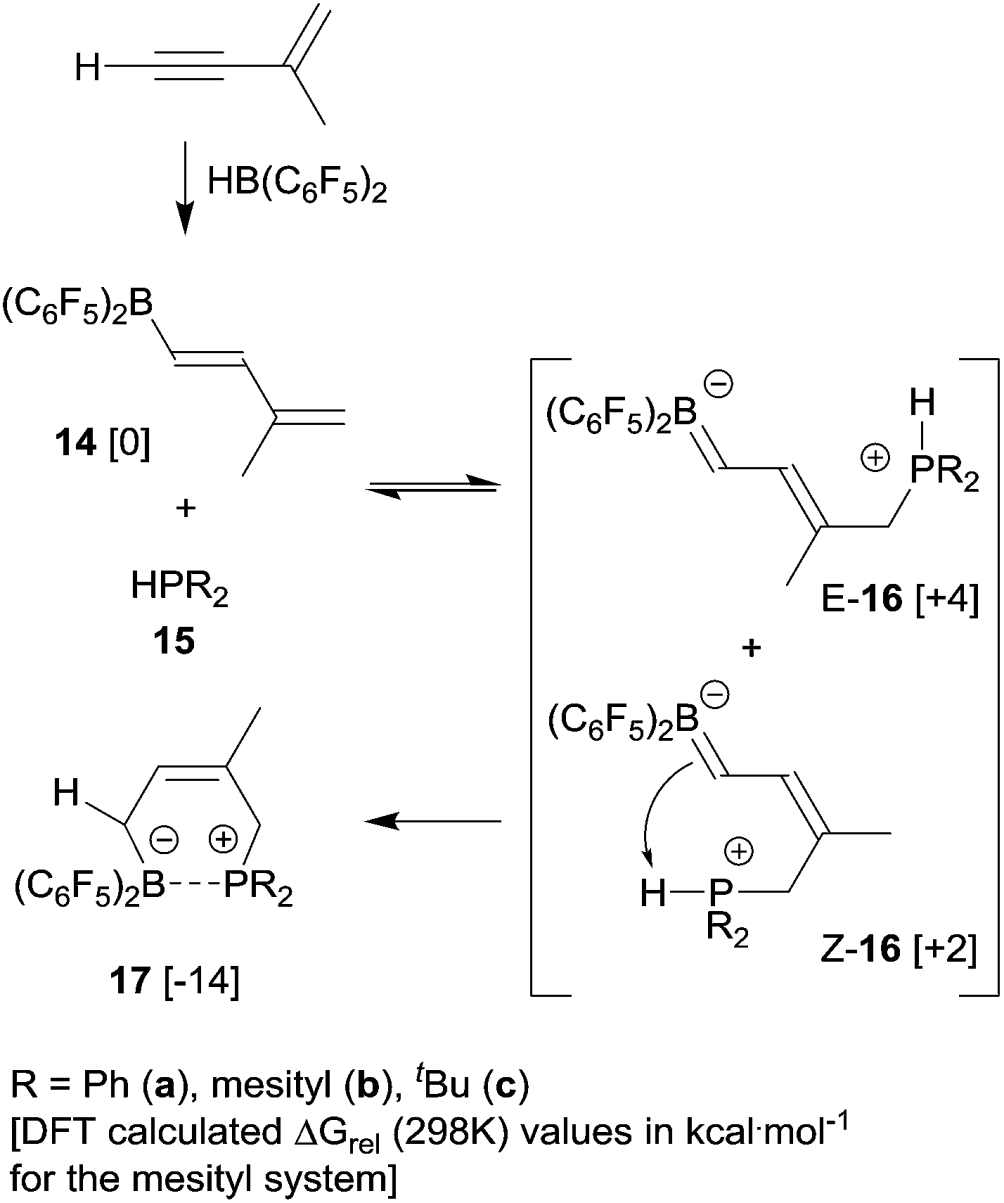


Knowing about the pronounced stabilization of carbanions in the α-position to boryl groups, especially to the easily introduced B(C_6_F_5_)_2_ substituent, as has become apparent from our study, it was tempting to search for uncatalyzed HPR_2_ addition reactions by utilizing this effect. We have, therefore, reacted a conjugated diene bearing a terminal B(C_6_F_5_)_2_ substituent **14**, with a small series of HPR_2_ reagents **15** [R: Ph (a), mesityl (b), *tert*-butyl (c)]. This resulted in clean 1,4-hydrophosphination under relatively mild reaction conditions (see Scheme 8).

For this study we generated the boryl substituted conjugated diene starting material **14** *in situ* by treatment of 2-methylbutenyne with Piers' borane [HB(C_6_F_5_)_2_] (see Scheme 8).^8b,23^ We ensured by an NMR experiment that the hydroboration reaction proceeded chemoselectively at the alkynyl moiety with an anti-Markovnikov orientation. The resulting dienyl borane **14** showed a ^11^B NMR signal at *δ* 58.8, which is typical for a Lewis acidic planar-tricoordinate RB(C_6_F_5_)_2_ situation (^19^F NMR of C_6_F_5_: Δ*δ* ^19^F_m,p_ = 12.9 ppm). Compound **14** shows ^1^H NMR resonances of the dienyl framework at *δ* 7.21, 6.90 (^3^ *J*_HH_ = 17.4 Hz, *trans*-CH=CH–) and 5.14, 5.11 (=CH_2_), respectively.

The reaction of *in situ* generated dienyl borane **14** with HPPh_2_ went to completion within 3 days at 80 °C (in toluene). Workup then gave the hydrophosphination product **17a** as a white solid in 74% yield. It was characterized by C,H-elemental analysis, spectroscopy and X-ray diffraction (for details see Tables 3 and 4 and the ESI†).

**Table 3:** Selected structural data of compounds **17** ^*a*^

Compound	**17a**	**17b**	**17c**
R	Ph	Mes	^*t*^Bu
B1–P1	2.052(2)	2.085(2)	2.069(2)
P1–C1	1.835(2)	1.834(2)	1.827(2)
C1–C2	1.513(2)	1.509(3)	1.504(3)
C2–C3	1.334(3)	1.324(3)	1.321(3)
C3–C4	1.499(3)	1.497(3)	1.497(3)
B1–C4	1.649(2)	1.643(3)	1.649(3)
C1–P1–B1	104.7(1)	101.9(1)	98.3(1)
C4–B1–P1	101.9(1)	97.0(1)	103.1(1)
P1–B1–C4–C3	–49.9(2)	67.8(2)	25.9(2)
B1–P1–C1–C2	48.8(1)	17.8(2)	57.3(2)
∑P1^CCC^	317.1	316.4	317.2
∑B1^CCC^	338.2	332.8	326.4

^*a*^Bond lengths in Å, angles in deg.

**Table 4:** Selected NMR data of the compounds **17** ^*a*^

Compound	**17a**	**17b**	**17c**
R	Ph	Mes	^*t*^Bu
^31^P	1.3	1.7	15.0
^11^B	–13.6	–8.1	–13.9
C1	27.7	31.6	21.5
C2	125.2	128.3	124.7
C3	132.1	128.4	131.1
C4	21.7	19.5	27.3
1-H	2.98	3.98/2.71	2.38
3-H	6.09	5.46	5.78
4-H	2.13	2.23/1.71	2.03
^19^F (*o*)	–129.2	–125.5/–127.4/–128.6/–131.5	–124.0/–125.5/–129.8/–132.1
^19^F (*p*)	–159.3	–158.0/–160.9	–157.4/–160.7
^19^F (*m*)	–165.0	–164.7 (2F)/–165.2/–165.8	–163.0/–163.8/–164.0/–165.0

^*a*^In CD_2_Cl_2_, chemical shifts in ppm, *δ*-scale.

**Fig. 4:**
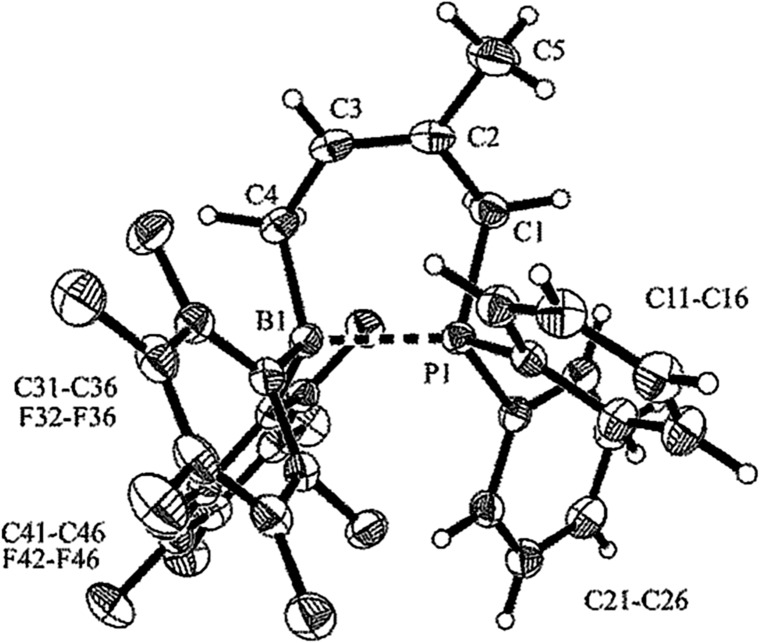
Molecular structure of compound **17a** (thermal ellipsoids are shown with 30% probability).

The X-ray crystal structure analysis of compound **17a** has confirmed the 1,4-hydrophosphination reaction to the boryl-diene. The compound shows a boat shaped central six-membered framework with a *Z*-configurated carbon–carbon double bond. There is a marked boron–phosphorus interaction. Both the boron and the phosphorus atom feature pseudo-tetrahedral coordination geometry (see Fig. 4 and Table 3). In solution compound **17a** undergoes a rapid conformational equilibration of the central heterocyclohexene ring.^24^ From the temperature dependent ^19^F NMR spectra a Gibbs activation energy of Δ*G* ^‡^(243 K) = 10.9 ± 0.3 kcal mol^–1^ has been obtained for this symmetrisation process. Consequently, we have monitored the ^1^H/^13^C NMR signals of a pair of symmetry-equivalent phenyl substituents at phosphorus and the ^19^F NMR signals of a pair of symmetry-equivalent C_6_F_5_ groups at boron (Δ*δ* ^19^F_m,p_ = 5.7) at ambient temperature (for further details see Table 4 and the ESI,† where the respective spectra are depicted).

We also reacted the *in situ* generated boryl-diene **14** with dimesitylphosphane **15b**. The reaction mixture was kept in toluene for 16 h at 60 °C. The 1,4-hydrophosphination product **17b** was then isolated in 70% yield (see Scheme 8). It was characterized by X-ray diffraction (see Table 3; the structure is depicted in the ESI†) and by spectroscopy (see Table 4). It shows the typical NMR features of a P/B Lewis pair which exhibits a marked P–B interaction as is typical for many intramolecular Lewis pairs.^6,25^

Finally, we also prepared the P/B product **17c** as the third member of this series. This was obtained in a similar way by selective uncatalyzed 1,4-hydrophosphination of the boryl-diene **14** with HP^*t*^Bu_2_ (toluene, 16 h, 60 °C). Product **17c** was isolated after workup in 79% yield. It was also characterized by C,H-elemental analysis, spectroscopy (see Table 4) and X-ray diffraction (see Table 3). Single crystals of compound **17c** were obtained from dichloromethane/pentane by the diffusion method. A view of the molecular structure of compound **17c** is depicted in the ESI.† Compound **17c** also shows dynamic temperature dependent NMR spectra due to the rapid conformational equilibration of the heterocyclohexene framework (Δ*G* ^‡^(268 K) = 11.3 ± 0.3 kcal mol^–1^, for further details including the depicted NMR spectra see the ESI†).

**Fig. 5:**
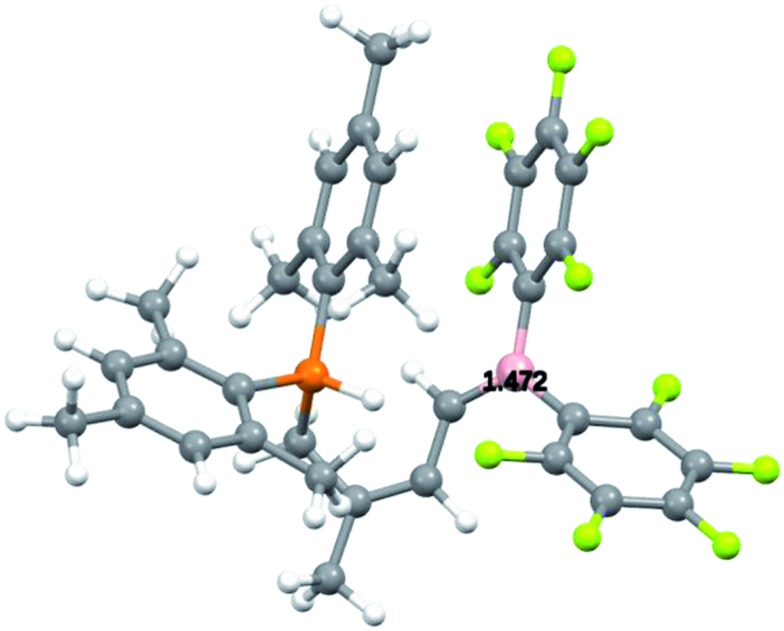
DFT-calculated structure of the reactive intermediate *Z*-**16b**.

Although we have not observed any intermediate in this reaction experimentally, it is tempting to assume that the boryl-diene **14** is attacked by the *sec*-phosphane nucleophiles in the distal conjugated position (=CH_2_).^4^ This would lead to the phosphonium/borata-alkene zwitterionic intermediates E-**16** and Z-**16**, respectively (see Scheme 8 and Fig. 5). Subsequent intramolecular proton transfer from the phosphonium unit to the borata-alkene carbanion centre would then directly give the hydrophosphination product that in all three examples of our system is found to contain a marked internal borane-phosphane Lewis acid–Lewis base interaction. The internal proton transfer reaction was actually probed by a respective deuteration experiment using the D-PPh_2_ reagent **15a** (for details see the ESI†). The phosphane addition reaction is probably reversible which would allow for a complete subsequent proton transfer reaction to eventually give the observed product **17**.

Our DFT analysis of this system (carried out for the mes_2_P substituted example) shows that the observed preferred regioselective addition of the secondary phosphane nucleophile at the terminal carbon atom of the dienyl borane is probably governed by the enhanced stabilization energy of the resulting borata-diene system **16b** (see Scheme 8). This makes the first step of the sequence, here the HPmes_2_ addition, only weakly endergonic. The subsequent H-transfer reaction is strongly exergonic, which partly comes from the formation of the borane-phosphane Lewis pair.

## Conclusions

Our study has shown that the (C_6_F_5_)_2_B substituent stabilizes an adjacent carbanion site substantially. The stabilization energy of such an α-boryl carbanion [(C_6_F_5_)_2_B–CHR]^–^ in dichloromethane solution is in the order of 56 kcal mol^–1^ relative to the cyclopentyl carbanion. That is approximately in the same order of magnitude as the stabilization of the cyclopentadienyl anion. This large stabilization of the [(C_6_F_5_)_2_B–CHR]^–^ anion can be expressed by its borata-alkene [(C_6_F_5_)_2_B=CHR]^–^ resonance form. The X-ray crystal structure analyses of the few examples of such isolated species (see Scheme 2 above) actually show olefin-like geometries for the borata-alkene moiety with markedly shortened B=C bond distances of around 1.44 Å (the adjacent B–C(aryl) bonds are much longer at >1.60 Å).^2,4^

The high degree of carbanion stabilization by the (C_6_F_5_)_2_B boryl substituent is to a large extent due to the presence of the fluorine substituents at its periphery. Their substitution for *e.g.* mesityl substituents at boron led to a decrease of stabilization by *ca.* 16 kcal mol^–1^. Consequently, boranes containing the mes_2_B group are much less CH acidic than the (C_6_F_5_)_2_B boranes (by *ca.* 11 pK_a_ units) and 9-BBN boranes are even less CH acidic (by *ca.* 16 p*K*_a_ units).

In frustrated P/B FLPs there might be a possibility to see internal proton transfer form the α-CH-position to the boryl substituent to the phosphane Lewis base. However, our study has shown (in accord with experiment) that this is not very likely to occur in many of the typical intramolecular FLPs used in this chemistry. Our study has, however, also shown that there might be exceptions and we have identified the system **7** as such by its trapping reaction with Piers' borane [HB(C_6_F_5_)_2_]. Although it seems that the formation of the borata-alkenes does not play a dominant role in frustrated Lewis pair chemistry, it might do so in other reactions. Our non-catalyzed 1,4-hydrophosphination reaction of the boryl-diene system **14** may be such a case where the thermodynamic stabilization of the borata-diene intermediate might potentially be a decisive factor in having this reaction take place as observed under the relatively mild reaction conditions.^26^

## Experimental section

### Preparation of compound **8**

Compound **4a** (38.4 mg, 0.1 mmol) was added at r.t. to a solution of HB(C_6_F_5_)_2_ (69.2 mg, 0.2 mmol) in toluene (1 mL). Then the reaction mixture was stirred for 1 h. The obtained yellow solution was layered with pentane and stored in the freezer at –32 °C for 2 days until a white, amorphous powder precipitated. The liquid was removed and the solid was washed with pentane (3 × 2 mL). Then the solid was dried *in vacuo* to give compound **8** (60.2 mg, 56 % yield) as a white powder. Anal. calc. for C_51_H_31_B_2_F_20_P_1_: C, 56.91; H, 2.90. Found: C, 56.11; H 2.82. For the NMR data see the ESI†.

### Preparation of compound **9**

*t*-Bu_3_P (20.2 mg, 0.1 mmol) was added at r.t. to a solution of compound **8** (107.6 mg, 0.1 mmol) in CD_2_Cl_2_ (1 mL). Then the reaction mixture was stirred for 1 h. Crystals of compound **9** suitable for the X-ray crystal structure analysis were obtained from the reaction mixture by slow evaporation of CD_2_Cl_2_ at –36 °C.

### Preparation of compound **17a**

Bis(pentafluorophenyl)borane (0.346 g, 1.0 mmol, 1 equiv.) and 2-methylbut-1-en-3-yne (0.072 g, 1.1 mmol, 1.1 equiv.) were suspended in toluene (5 mL) and stirred for 4 h at room temperature. Then diphenylphosphane (0.186 g, 1.0 mmol, 1 equiv.) in toluene (5 mL) was added and the reaction mixture was heated at 80 °C for 3 days. Subsequently all volatiles were removed *in vacuo* and the obtained residue was washed with cold pentane (3 × 1 mL). After drying *in vacuo* compound **17a** (0.442 g, 0.74 mmol, 74% yield) was obtained as a white solid. Crystals suitable for the X-ray crystal structure analysis were obtained by slow diffusion of pentane to a dichloromethane solution of compound **17a** at –35 °C. M.p.: 184 °C. Anal. Calc. for C_29_H_18_BF_10_P: C: 58.22; H: 3.03. Found: C: 58.41; H: 2.82. For the NMR data see the ESI†.

### Preparation of compound **17b**

The reaction procedure was similar to that described for the preparation of compound **17a**: bis(pentafluorophenyl)borane (0.346 g, 1.0 mmol, 1 equiv.) and 2-methylbut-1-en-3-yne (0.072 g, 1.1 mmol, 1.1 equiv.) in toluene (5 mL) reacted with dimesitylphosphane (0.270 g, 1.0 mmol, 1 equiv.) in toluene (5 mL) after heating at 60 °C for 16 h to give compound **17b** (0.477 g, 0.70 mmol, 70% yield) as a light yellow solid. Crystals suitable for the X-ray crystal structure analysis were obtained by slow diffusion of pentane to a dichloromethane solution of compound **17b** at –35 °C. M.p.: 167 °C. Anal. Calc. for C_35_H_30_BF_10_P: C: 61.60; H: 4.43. Found: C: 61.52; H: 4.29. For the NMR data see the ESI†.

### Preparation of compound **17c**

The reaction procedure was similar to that described for the preparation of compound **17a**: bis(pentafluorophenyl)borane (0.346 g, 1.0 mmol, 1 equiv.) and 2-methylbut-1-en-3-yne (0.072 g, 1.1 mmol, 1.1 equiv.) in toluene (5 mL) reacted with di-*tert*-butylphosphane (0.146 g, 1.0 mmol, 1 equiv.) in toluene (5 mL) after heating at 60 °C for 16 h to give compound **17c** (0.439 g, 0.79 mmol, 79% yield) as a white solid. Crystals suitable for the X-ray crystal structure analysis were obtained by slow diffusion of pentane to a dichloromethane solution of compound **17c** at –35 °C. M.p.: 187 °C. Anal. Calc. for C_25_H_26_BF_10_P: C: 53.79; H: 4.69. Found: C: 54.13; H: 4.53. For the NMR data see the ESI†.
