# Supplementary material for: α-CH acidity of alkyl–B(C6F5)2 compounds – the role of stabilized borata-alkene formation in frustrated Lewis pair chemistry
Source: Chem Sci. 2014 Sep 26;6(1):816–25. doi: 10.1039/c4sc01711k (PMC5592807; doi:10.1039/c4sc01711k)
Supplement: Supplementary file 1 [file SC-006-C4SC01711K-s001.pdf]

Electronic Supplementary Material (ESI) for Chemical Science.
This journal is © The Royal Society of Chemistry 2014

α-CH Acidity of Alkyl-B(C_6F_5)$_2$ Compounds – the Role of Stabilized Borataalkene Formation in Frustrated Lewis Pair Chemistry

Philip Moquist,† Guo-Qiang Chen,† Christian Mück-Lichtenfeld, Kathrin Bussmann, Constantin G. Daniliuc, Gerald Kehr, and Gerhard Erker*

Organisch-Chemisches Institut, Universität Münster, Corrensstrasse 40, 48149 Münster, Germany

†these authors contributed equally to this work

Supporting Information

General Procedure. All syntheses involving air- and moisture sensitive compounds were carried out using standard Schlenk-type glassware (or in a glove box) under an atmosphere of argon. Solvents were dried and stored under an argon atmosphere. NMR spectra were recorded on a Agilent DD2- 500 MHz (^{1}H: 500 MHz, ^{13}C: 126 MHz, ^{19}F: 470 MHz, ^{11}B: 160 MHz, ^{31}P: 202 MHz) and on a Agilent DD2- 600 MHz (^{1}H: 600 MHz, ^{13}C: 151 MHz, ^{19}F: 564 MHz, ^{11}B: 192 MHz, ^{31}P: 243 MHz). ^{1}H NMR and ^{13}C NMR: chemical shifts are given relative to TMS and referenced to the solvent signal. ^{19}F NMR: chemical shifts are given relative to $CFCl_3$ (δ = 0, external reference), ^{11}B NMR: chemical shifts are given relative to $BF_3{\cdot}Et_2O$ (δ = 0, external reference), ^{31}P NMR: chemical shifts are given relative to H_3PO_4 (85% in D_2O) (δ = 0, external reference). NMR assignments were supported by additional 2D NMR experiments. Elemental analyses were performed on a Elementar Vario El III. IR spectra were recorded on a Varian 3100 FT-IR (Excalibur Series). Melting points and decomposition points were obtained with a DSC 2010 (TA Instruments). HRMS was recorded on GTC Waters Micromass (Manchester, UK).

X-Ray crystal structure analyses. Data sets were collected with a Nonius KappaCCD diffractometer. Programs used: data collection, COLLECT (R. W. W. Hooft, Bruker AXS, 2008, Delft, The Netherlands); data reduction Denzo-SMN (Z. Otwinowski, W. Minor, *Methods Enzymol.* **1997**, *276*, 307-326); absorption correction, Denzo (Z. Otwinowski, D.

Borek, W. Majewski, W. Minor, *Acta Crystallogr.* **2003**, *A59*, 228-234); structure solution SHELXS-97 (G. M. Sheldrick, *Acta Crystallogr.* **1990**, *A46*, 467-473); structure refinement SHELXL-97 (G. M. Sheldrick, *Acta Crystallogr.* **2008**, *A64*, 112-122) and graphics, XP (BrukerAXS, 2000). Thermals ellipsoids are shown with 30% probability, *R*-values are given for observed reflections, and wR^2 values are given for all reflections. *Exceptions and special features*: The n-Butyl group at N1 atom is disordered over two positions. Several restraints (SADI, SIMU, ISOR and SAME) were used in order to improve refinement stability. For the compound **17b** a disordered half dichloromethane molecule and for the compound **17c** one badly disordered dichloromethane molecule were found in the asymmetrical unit and could not be satisfactorily refined. The program SQUEEZE (A. L. Spek J. Appl. Cryst., 2003, 36, 7-13) was therefore used to remove mathematically the effect of the solvents. The quoted formula and derived parameters are not included the squeezed solvent molecules.

Materials. Chlorodimesitylphosphane [Bartlett, R. A.; Olmstead, M. M.; Power, P. P.; Siegel, G. A. *Inorg. Chem.* **1987**, *26*, 1941-1946.], $B(C_6F_5)_3$ (**1**) [(a) Wang, C.; Erker, G.; Kehr, G.; Wedeking, K.; Fröhlich, R. *Organometallics*, **2005**, *24*, 4760–4773. (b) Massey, A. G.; Park, A. J. *J. Organomet. Chem.* **1964**, *2*, 245–250. (c) Massey, A. G.; Park, A. J.; Stone, F. G. A. *Proc. Chem. Soc.* **1963**, 212.], $HB(C_6F_5)_2$ [(a) D. J. Parks, W. E. Piers, G. P. A. Yap, *Organometallics* **1998,** *17*, 5492; (b) D. J. Parks, R.E. v. H. Spence and W. E. Piers, *Angew. Chem. Int. Ed. Engl.* 1995, **34**, 809.] and $HPMes_2$ [Bartlett, R. A.; Olmstead, M. M.; Power, P. P.; Sigel, G. A. *Inorg. Chem.*, 1987, **26**, 1941-1946.] were prepared according to the literature. **10a** and **10b** were synthesized by a modified procedure of K. A. Fallis, G. K. Anderson, N. P. Rath, *Organometallics*, **1992**, *11*, 885-888.

1-Indenyldimesitylphosphane (4a)

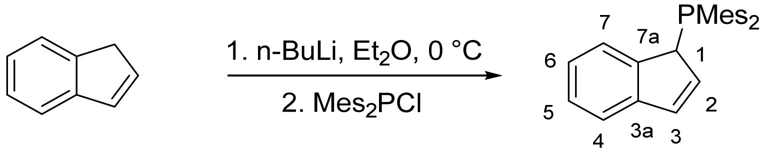

Scheme S1

Indene (1.16 g, 10.0 mmol) and Et_2O (40 mL) were added to a 100 mL Schlenk flask. The solution was cooled to 0 °C and *n*-BuLi (6.25 mL, 1.6 M in hexane, 10 mmol) was added. The yellow solution was stirred for 30 min and Mes_2PCl (3.04 g, 10.0 mmol, 0.5 M solution in Et_2O) was added and the reaction was stirred at 0 °C for 1.5 h. The cloudy suspension was filtered to remove LiCl and then concentrated in vacuo at 0 °C. The obtained residue was

washed with pentane to give compound **4a** as a white powder (2.55 g, 66% yield), which could be stored indefinitely at -36 °C. **MS:** exact mass calcd. for $C_{27}H_{29}P^{+}H^{+}$: 385.2085 found: 385.2085. **Anal.** calc. for $C_{27}H_{29}P$: C, 84.34; H, 7.60. Found: C, 83.35; H, 7.39.

^{1}H NMR (500 MHz, 299 K, CD_2Cl_2): δ = 7.43 (m, 1H, 4-H), 7.26 (m, 1H, 5-H), 6.98 (m, 1H, 6-H), 6.97 (m, 1H, 3-H), 6.87 (m, 2H, *m*-Mes^{a}), 6.83 (m, 2H, *m*-Mes^{b}), 6.64 (m, 1H; 7-H), 6.63 (m, 1H, 2-H), 5.27 (m, 1H, 1-H), 2.38 (d, $^{3}J_{PH}$ = 1.5 Hz, 6H, *o*-$CH_3^{Mes,b}$), 2.30 (s, 3H, *p*-$CH_3^{Mes,a}$), 2.253 (s, 3H, *p*-$CH_3^{Mes,b}$), 2.248 (m, 6H, *o*-$CH_3^{Mes,a}$).

^{13}C{^{1}H} NMR (126 MHz, 299 K, CD_2Cl_2): δ = 145.1 (d, $^{3}J_{PC}$ = 1.3 Hz, C3a), 145.0 (d, $^{2}J_{PC}$ = 12.5 Hz, C7a), 144.0 (d, $^{2}J_{PC}$ = 15.6 Hz, *o*-Mes^{a}), 142.4 (d, $^{2}J_{PC}$ = 16.0 Hz, *o*-Mes^{b}), 139.2 (*p*-Mes^{a}), 138.1 (*p*-Mes^{b}), 137.6 (d, $^{2}J_{PH}$ = 8.3 Hz, C2), 132.8 (d, $^{1}J_{PC}$ = 33.2 Hz, *i*-Mes^{b}), 132.1 (d, $^{3}J_{PC}$ = 7.3 Hz, C3), 132.0 (d, $^{2}J_{PC}$ = 31.7 Hz, *i*-Mes^{a}), 130.6 (d, $^{3}J_{PC}$ = 2.7 Hz, *m*-Mes^{b}), 130.3 (d, $^{3}J_{PC}$ = 3.8 Hz, *m*-Mes^{a}), 126.9 (d, $^{5}J_{PC}$ = 1.3 Hz, C5), 125.2 (d, $^{3}J_{PC}$ = 5.2 Hz, C7), 124.8 (d, $^{4}J_{PC}$ = 1.3 Hz, C6), 121.6 (C4), 47.5 (d, $^{1}J_{PC}$ = 27.1 Hz, C1), 23.2 (d, $^{3}J_{PC}$ = 14.8 Hz, *o*-$CH_3^{Mes,b}$), 23.0 (d, $^{3}J_{PH}$ = 15.3 Hz, *o*-$CH_3^{Mes,a}$), 21.1 (*p*-$CH_3^{Mes,a}$), 20.9 (*p*-$CH_3^{Mes,b}$).

^{31}P{^{1}H} NMR (202 MHz, 299 K, CD_2Cl_2): δ = −14.5 ($\nu_{1/2}$ ~ 2 Hz).

^{31}P NMR (202 MHz, 299 K, CD_2Cl_2): δ = −14.5 ($\nu_{1/2}$ ~ 20 Hz).

^{1}H, ^{1}H GCOSY (500/500 MHz, 299 K, CD_2Cl_2)[selected traces]**:** δ^{1}H/δ^{1}H = 7.26 / 7.43, 6.98 (5-H / 4-H, 6-H), 6.98 / 7.26, 6.64 (6-H / 5-H, 7-H), 6.87 / 2.30, 2.25 (*m*-Mes^{a} / *p*-$CH_3^{Mes,a}$, *o*-$CH_3^{Mes,a}$), 6.83 / 2.38, 2.25 (*m*-Mes^{b} / *o*-$CH_3^{Mes,b}$, *p*-$CH_3^{Mes,b}$).

^{1}H{^{1}H} TOCSY (500 MHz, 299 K, CD_2Cl_2)[selected experiment]**:** δ$^{1}H_{ir}$/δ$^{1}H_{res}$ = 5.27 / 6.97, 6.63 (1-H / 3-H, 2-H).

^{1}H, ^{13}C GHSQC (500/126 MHz, 299 K, CD_2Cl_2): δ^{1}H/δ^{13}C = 7.43 / 121.6 (C4), 7.26 / 126.9 (C5), 6.98 / 124.8 (C6), 6.97 / 132.1 (C3), 6.87 / 130.3 (*m*-Mes^{a}), 6.83 / 130.6 (*m*-Mes^{b}), 6.64 / 125.2 (C7), 6.63 / 137.6 (C2), 5.27 / 47.5 (C1), 2.38 / 23.2 (*o*-$CH_3^{Mes,b}$), 2.30 / 21.1 (*p*-$CH_3^{Mes,a}$), 2.253 / 20.9 (*p*-$CH_3^{Mes,b}$), 2.248 / 23.0 (*o*-$CH_3^{Mes,a}$).

^{1}H, ^{13}C GHMBC (500/126 MHz, 299 K, CD_2Cl_2)[selected traces]**:** δ^{1}H/δ^{13}C = 7.43 / 145.0, 132.1, 124.8 (4-H / C7a, C3, C6), 6.64 / 145.1, 126.9, 47.5 (7-H / C3a, C5, C1), 2.38 / 142.4, 132.8, 130.6 (*o*-$CH_3^{Mes,b}$ / *o*-Mes^{b}, *i*-Mes^{b}, *m*-Mes^{b}), 2.30 / 139.2, 130.3 (*p*-$CH_3^{Mes,a}$ / *p*-Mes^{a}, *m*-Mes^{a}), 2.253 / 138.1, 130.6 (*p*-$CH_3^{Mes,b}$ / *p*-Mes^{b}, *m*-Mes^{b}), 2.248 / 144.0, 132.0, 130.3 (*o*-$CH_3^{Mes,a}$ / *o*-Mes^{a}, *i*-Mes^{a}, *m*-Mes^{a}).

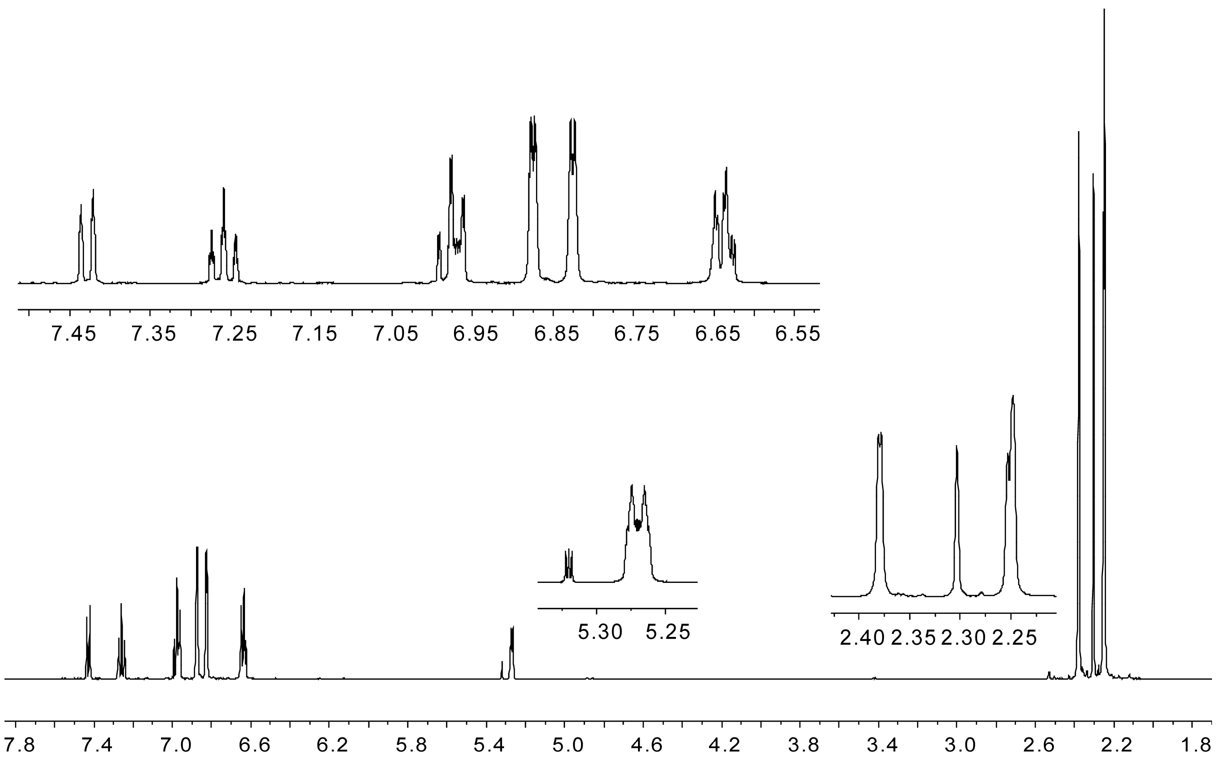

Figure S1: ^{1}H NMR (500 MHz, 299 K, CD_2Cl_2) of compound **4a**

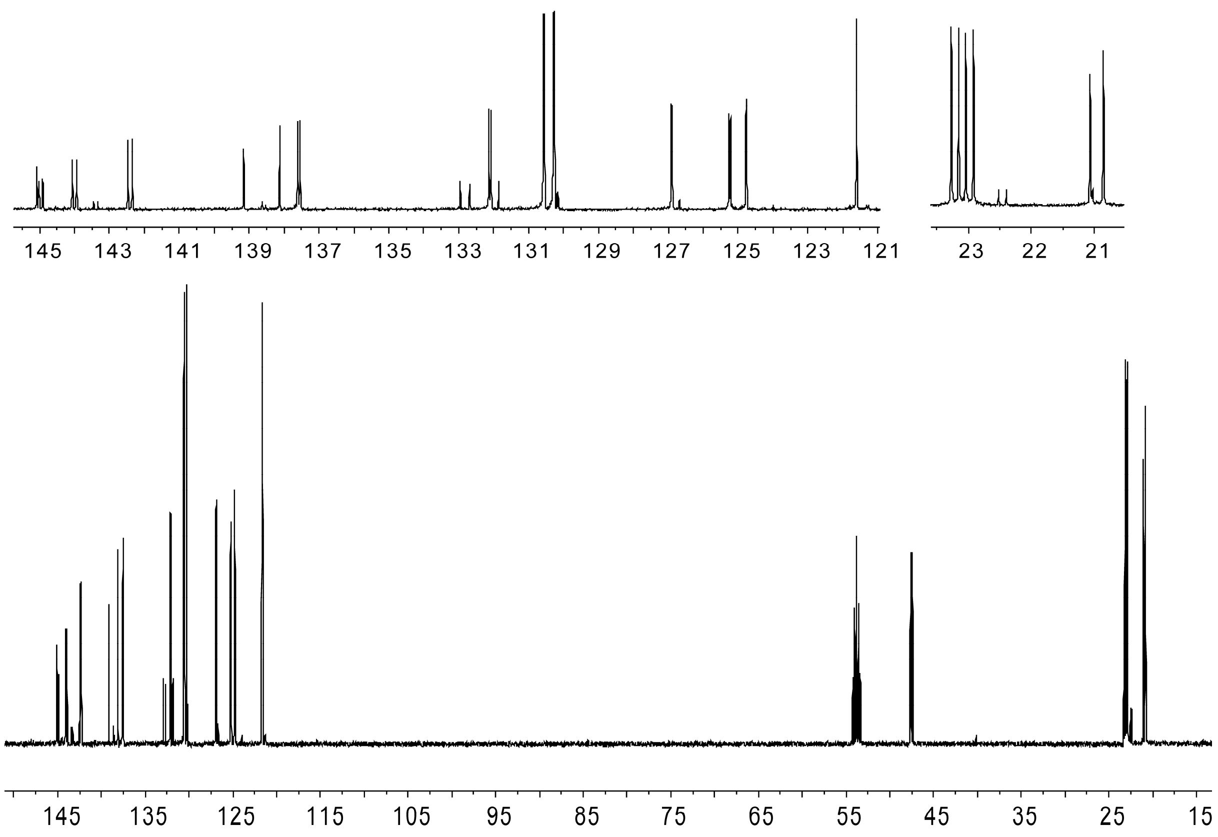

Figure S2: $^{13}C\{^{1}H\}$ NMR (126 MHz, 299 K, CD_2Cl_2) of compound **4a**

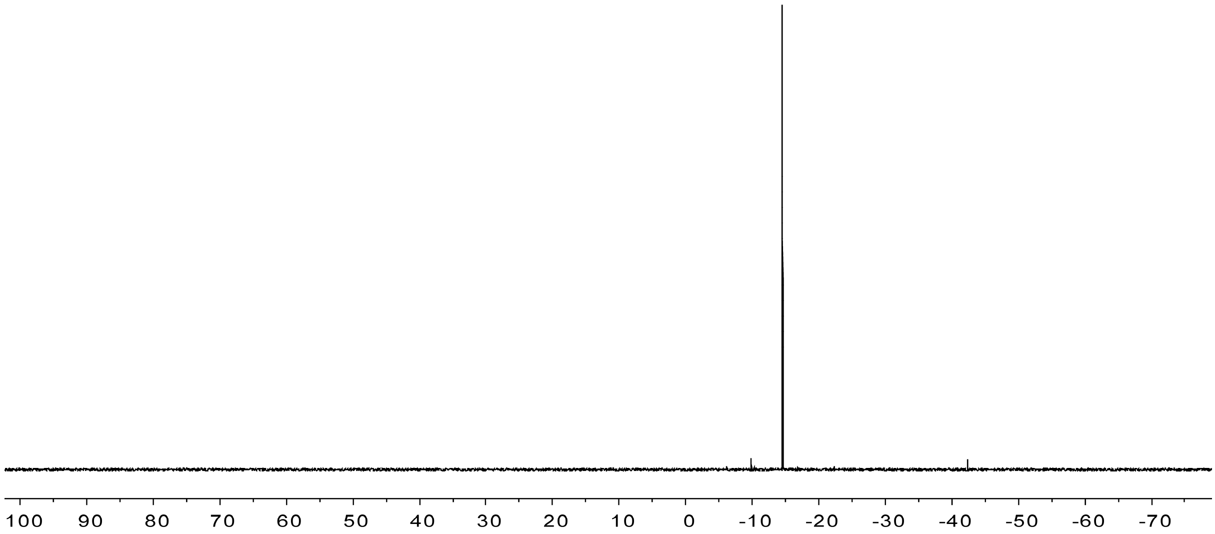

Figure S3: $^{31}P\{^1H\}$ NMR (202 MHz, 299 K, CD_2Cl_2) of compound **4a**

3-Indenyldimesitylphosphane (4b)

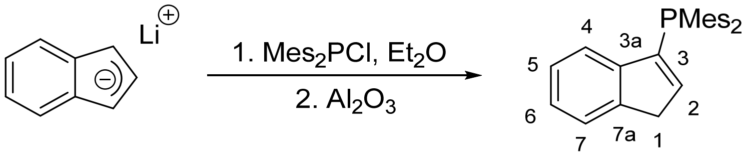

Scheme S2

A solution of indenyllithium (244 mg, 2.0 mmol) in Et_2O (10 mL) and a solution of Mes_2PCl (609 mg, 2.0 mmol) in Et_2O (10 mL) were prepared separately. The flasks were cooled to 0 °C and the Mes_2PCl solution was transferred to the indenyllithium solution. Then the reaction mixture was stirred for 1h and the LiCl was removed by filtration. Alumina (50 mg, 0.4 mmol, Activity III) was added to the solution and stirred for 1 h at r.t. The alumina was removed by filtering through Celite and the solvent of the filtrate was removed in vacuo. The resulting powder was washed with pentane (3 mL) to give compound **4b** (450 mg, 49%) as a white powder. **MS:** exact mass calcd. for $C_{27}H_{29}P^+H^+$: 385.2085 found: 385.2084 **Anal.** calc. for $C_{27}H_{29}P$: C, 84.34; H, 7.60. Found: C, 83.40; H, 7.53.

^{1}H NMR (500 MHz, 299 K, CD_2Cl_2): δ = 7.49 (m, 1H, 7-H), 7.36 (m, 1H, 4-H), 7.25 (m, 1H, 5-H), 7.22 (m, 1H, 6-H), 6.84 (m, 4H, *m*-Mes), 6.10 (m, 1H, 2-H), 3.41 (m, 2H, 1-H), 2.26 (s, 6H, *p*-CH_3^{Mes}), 2.23 (m, 12H, *o*-CH_3^{Mes}).

$^{13}C\{^1H\}$ NMR (126 MHz, 299 K, CD_2Cl_2): δ = 147.9 (d, $^2J_{PC}$ = 29.3 Hz, C3a), 144.5 (d, $^3J_{PC}$ = 6.4 Hz, C7a), 143.4 (d, $^2J_{PC}$ = 15.5 Hz, *o*-Mes), 140.7 (d, $^1J_{PC}$ = 13.8 Hz, C3), 138.6 (*p*-Mes), 138.5 (d, $^2J_{PC}$ = 1.9 Hz, C2), 130.2 (d, $^1J_{PC}$ = 15.2 Hz, *i*-Mes), 130.1 (d, $^3J_{PH}$ = 3.8 Hz,

m-Mes), 126.7 (C5), 125.2 (C6), 124.0 (d, ${}^4J_{PC}$ = 1.4 Hz, C7), 121.3 (d, ${}^3J_{PC}$ = 7.1 Hz, C4), 40.1 (d, ${}^3J_{PC}$ = 2.0 Hz, C1), 22.4 (d, ${}^3J_{PC}$ = 15.6 Hz, *o*-CH_3^{Mes}), 21.0 (*p*-CH_3^{Mes}).

${}^{31}P\{{}^1H\}$ NMR (202 MHz, 299 K, CD_2Cl_2): δ = −42.3 ($\nu_{1/2}$ ~ 1 Hz)

${}^{31}P$ NMR (202 MHz, 299 K, CD_2Cl_2): δ = −42.3 ($\nu_{1/2}$ ~ 20 Hz)

${}^1H\{{}^1H\}$ TOCSY (500 MHz, 299 K, CD_2Cl_2)[selected experiments]**:** $\delta^1H_{ir}/\delta^1H_{res}$ = 7.49 / 7.36, 7.25, 7.22 (7-H / 4-H, 5-H, 6-H), 6.84 / 2.26, 2.23 (*m*-Mes / *p*-, *o*-CH_3^{Mes}), 3.41 / 6.10 (1-H / 2-H).

${}^1H,{}^{13}C$ GHSQC (500/126 MHz, 299 K, CD_2Cl_2): $\delta^1H/\delta^{13}C$ = 7.49 / 124.0 (C7), 7.36 / 121.3 (C4), 7.25 / 126.7 (C5), 7.22 / 125.2 (C6), 6.84 / 130.1 (*m*-Mes), 6.10 / 138.5 (C2), 3.41 / 40.1 (C1), 2.26 / 21.0 (*p*-CH_3^{Mes}), 2.23 / 22.4 (*o*-CH_3^{Mes}).

${}^1H,{}^{13}C$ GHMBC (500/126 MHz, 299 K, CD_2Cl_2)[selected traces]**:** $\delta^1H/\delta^{13}C$ = 7.49 / 147.9, 126.7 (7-H / C3a, C5), 7.36 / 144.5, 125.2 (4-H / C7a, C6), 3.41 / 147.9, 144.5, 140.7, 138.5 (1-H / C3a, C7a, C3, C2), 2.26 / 138.6, 130.1 (*p*-CH_3^{Mes} / *p*-Mes, *m*-Mes), 2.23 / 143.4, 130.2, 130.1 (*o*-CH_3^{Mes} / *o*-Mes, *i*-Mes, *m*-Mes).

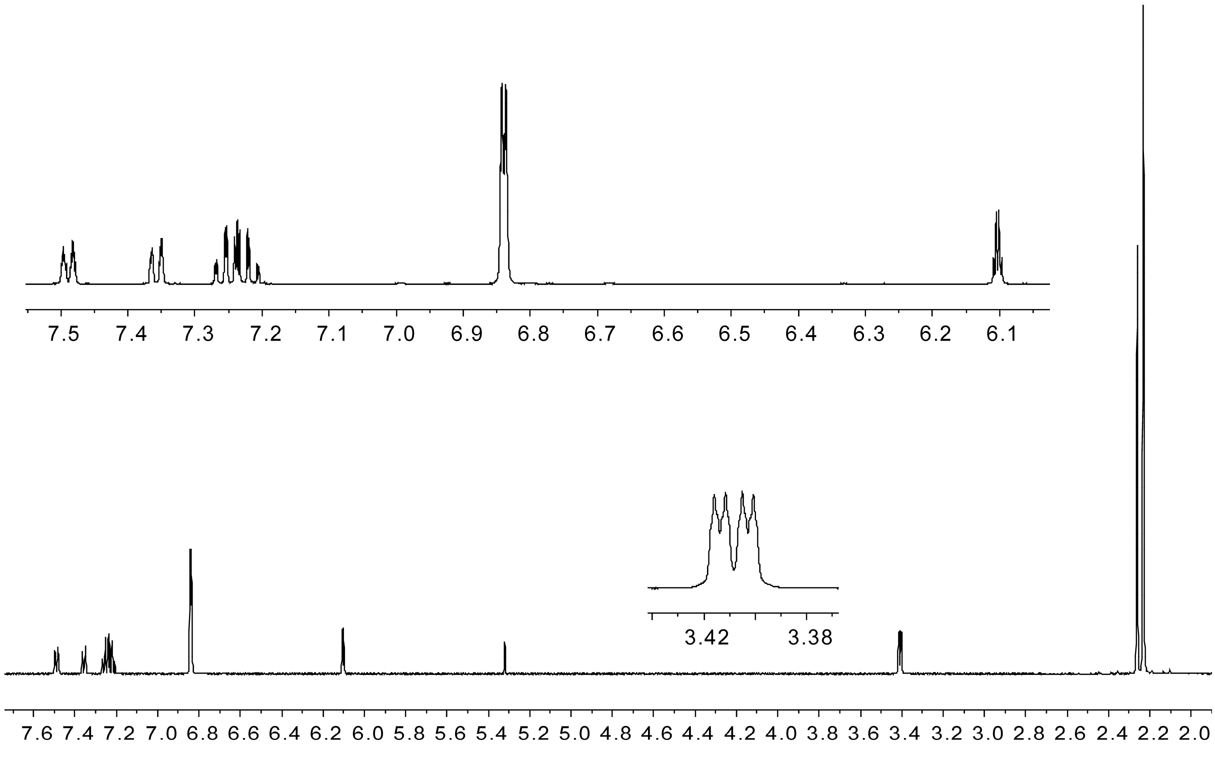

Figure S4: ${}^1H$ NMR (500 MHz, 299 K, CD_2Cl_2) of compound **4b**

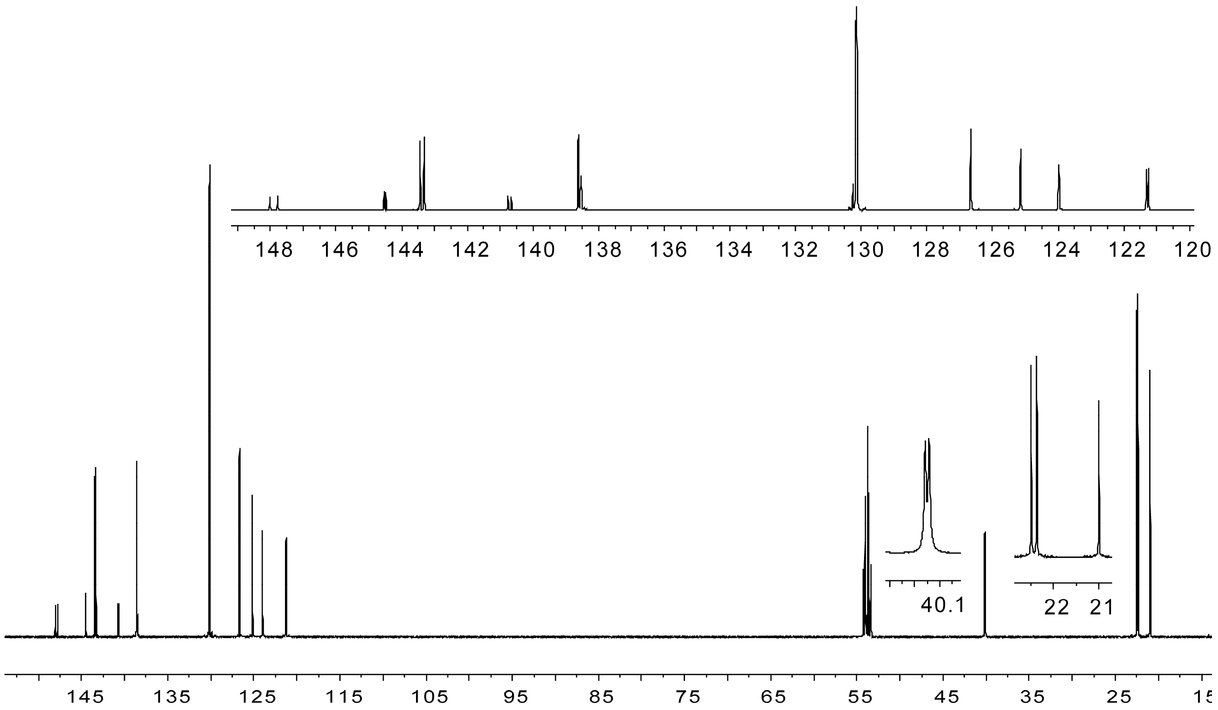

Figure S5: $^{13}C\{^{1}H\}$ NMR (126 MHz, 299 K, CD_2Cl_2) of compound **4b**

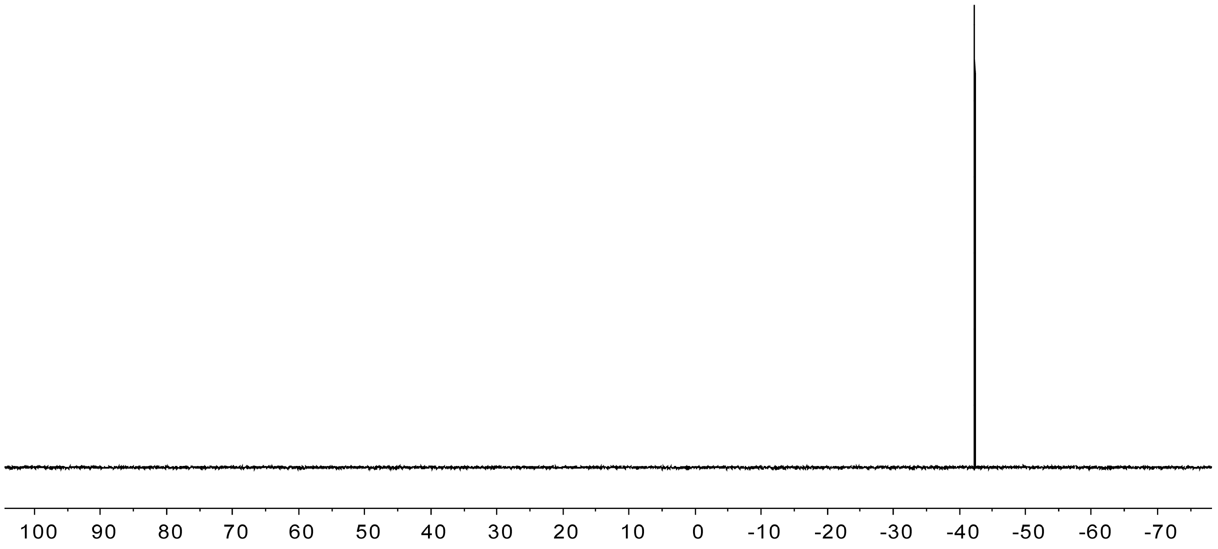

Figure S6: $^{31}P\{^{1}H\}$ NMR (202 MHz, 299 K, CD_2Cl_2) of compound **4b**

Compound 5

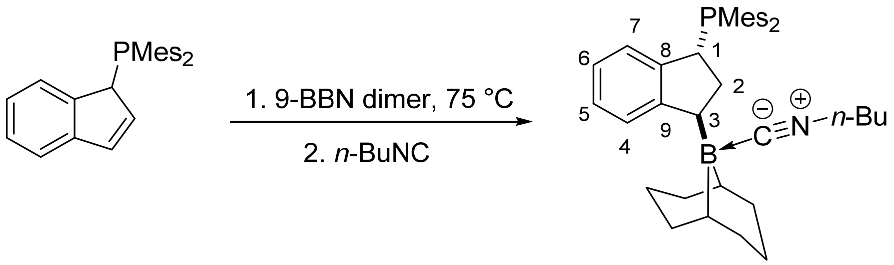

Scheme S3

Caution: Isocyanates are toxic and must be handled with due care.

Compound **4a** (38.4 mg, 0.1 mmol) was added to a solution of 9-BBN dimer (13.6 mg, 0.055 mmol) in toluene (1 mL) at r.t. Then the reaction mixture was heated at 75 °C for 8 h. After the reaction mixture was cooled to r.t. *n*-BuNC (8.3 mg, 0.1 mmol) was added. The resulting light yellow solution was stirred at r.t. for 1 h followed by the removal of all volatiles in vacuo. The obtained sticky oil was dissolved in pentane (5 mL) and the solvent was removed in vacuo. This procedure was repeated until a white solid was formed indicating complete removal of the toluene. Then the solid was washed with cold pentane (1 mL) and dried in vacuo to give compound **5** (60.2 mg, 56 %) as a white powder. Crystals suitable for the X-ray crystal structure analysis were obtained by slow diffusion of pentane into a solution of compound **5** in CH_2Cl_2 at -36 °C. **Anal.** calc. for $C_{40}H_{53}N_1B_1P_1$: C, 81.48; H, 9.06; N, 2.38. Found: C, 77.82; H, 9.22; N, 2.03.

^{1}H NMR (600 MHz, 299 K, CD_2Cl_2): δ = 7.27 (d, $^3J_{HH}$ = 7.5 Hz, 1H, 4-H), 6.97 (tm, $^3J_{HH}$ = 7.5 Hz, 1H, 5-H), 6.77 (m, 2H, *m*-Mes^a), 6.72 (m, 2H, *m*-Mes^b), 6.61 (tm, $^3J_{HH}$ = 7.5 Hz, 1H, 6-H), 6.09 (d, $^3J_{HH}$ = 7.5 Hz, 1H, 7-H), 4.66 (m, 1H, 1-H), 3.38 (2H), 1.28 (2H), 1.08 (2H), 0.76 (3H)(each m, Bu^{NC}), 2.95 (m, 1H, 3-H), 2.38, 2.18 (each m, each 1H, 2-H), 2.34 (s, 3H, *o*-$CH_3^{Mes,a}$), 2.23 (s, 3H, *p*-$CH_3^{Mes,b}$), 2.22 (s, 3H, *p*-$CH_3^{Mes,a}$), 2.05 (s, 3H, *o*-$CH_3^{Mes.b}$), 2.02/1.70, 1.97/1.56, 1.88/1.74, 1.86/1.52, 1.82/1.71, 1.74/1.55 (each m, each 1H, $CH_2^{9\text{-}BBN}$)[2], 1.39, 0.85 (each br, each 1H, $CH^{9\text{-}BBN}$), [[1] from the ghsqc experiment].

^{13}C{^{1}H} NMR (126 MHz, 299 K, CD_2Cl_2): δ = 154.4 (d, $^3J_{PC}$ = 3.6 Hz, C9), 144.5 (br d, $^2J_{PC}$ = 13.8 Hz, *o*-Mes^b), 144.1 (d, $^2J_{PC}$ = 10.4 Hz, C8), 142.1 (d, $^2J_{PC}$ = 14.0 Hz, *o*-Mes^a), 138.4 (d, $^4J_{PC}$ = 0.8 Hz, *p*-Mes^b), 138.1 (CN^{Bu})[2], 137.1 (*p*-Mes^a), 133.7 (d, $^1J_{PC}$ = 33.9 Hz, *i*-Mes^a), 132.9 (d, $^1J_{PC}$ = 22.4 Hz, *i*-Mes^b), 130.5 (d, $^3J_{PC}$ = 2.0 Hz, *m*-Mes^a), 129.4 (d, $^3J_{PC}$ = 3.8 Hz, *m*-Mes^b), 126.3 (d, $^5J_{PC}$ = 3.0 Hz, C5), 125.8 (d, $^3J_{PC}$ = 5.1 Hz, C7), 123.8 (d, $^4J_{PC}$ = 2.1 Hz, C4), 123.6 (d, $^4J_{PC}$ = 2.5 Hz, C6), 43.4 (m), 30.5, 19.6, 13.2 (Bu^{NC}), 41.1 (d, $^1J_{PC}$ = 19.4 Hz, C1), 38.3 (d, $^2J_{PC}$ = 23.4 Hz, C2), 35.2, 35.0, 30.21, 30.15, 25.9, 24.9 ($CH_2^{9\text{-}BBN}$), 34.4 (br, C3), 23.4, 22.7 (br, $CH^{9\text{-}BBN}$)[1], 23.0 (d, $^3J_{PC}$ = 12.9 Hz, *o*-$CH_3^{Mes,a}$), 22.5 (d, $^3J_{PC}$ = 15.1 Hz, *o*-

$CH_3^{Mes,b}$), 21.0 (p-$CH_3^{Mes,b}$), 20.8 (p-$CH_3^{Mes,a}$), [[1] from the ghsqc experiment; [2] from the ghmbc experiment].

$^{31}P\{^1H\}$ NMR (243 MHz, 299 K, CD_2Cl_2): $\delta = -17.7$ ($\nu_{1/2} \sim 15$ Hz).

^{31}P NMR (243 MHz, 299 K, CD_2Cl_2): $\delta = -17.7$ (m).

$^{11}B\{^1H\}$ NMR (192 MHz, 299 K, CD_2Cl_2): $\delta = -13.7$ ($\nu_{1/2} \sim 300$ Hz).

^{11}B NMR (192 MHz, 299 K, CD_2Cl_2): $\delta = -13.7$ ($\nu_{1/2} \sim 300$ Hz).

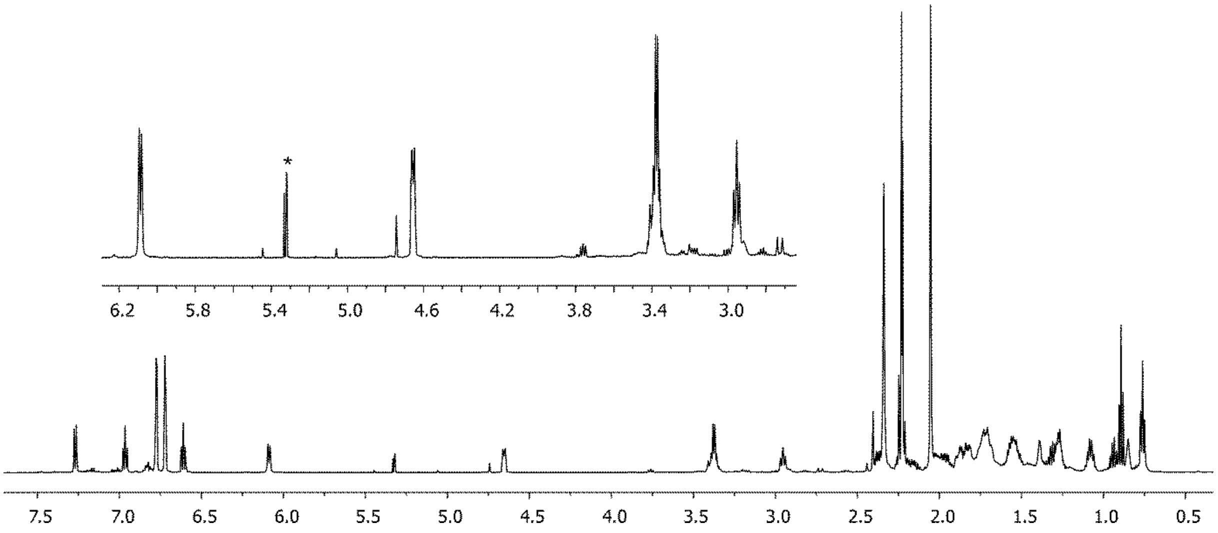

Figure S7: ^1H NMR (600 MHz, 299 K, CD_2Cl_2(*)) of compound **5**

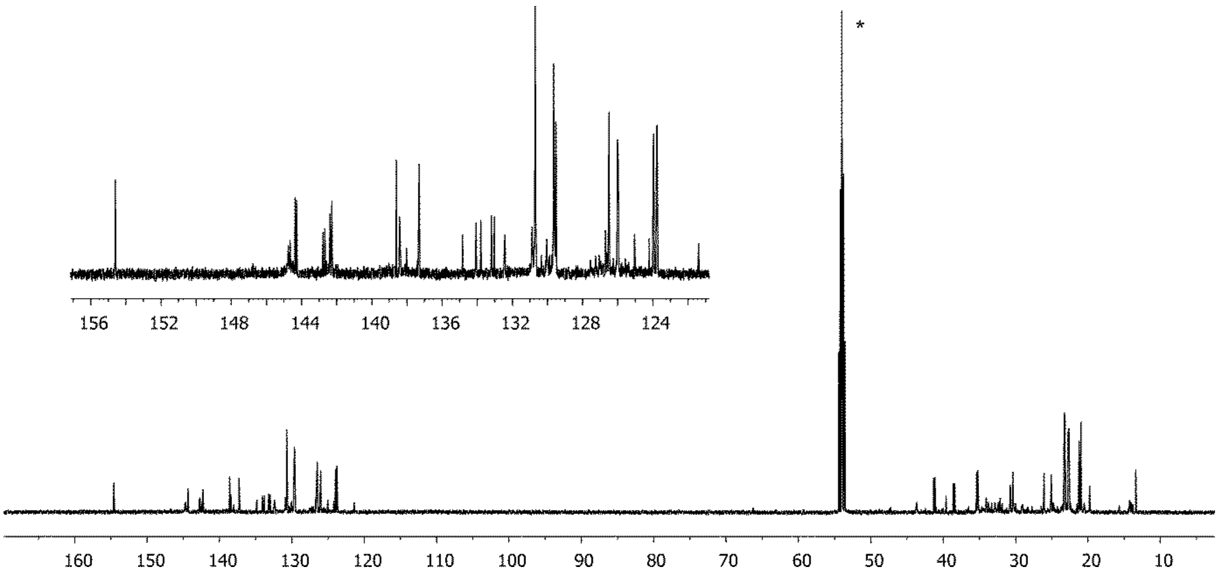

Figure S8: $^{13}C\{^1H\}$ NMR (126 MHz, 299 K, CD_2Cl_2(*)) of compound **5**

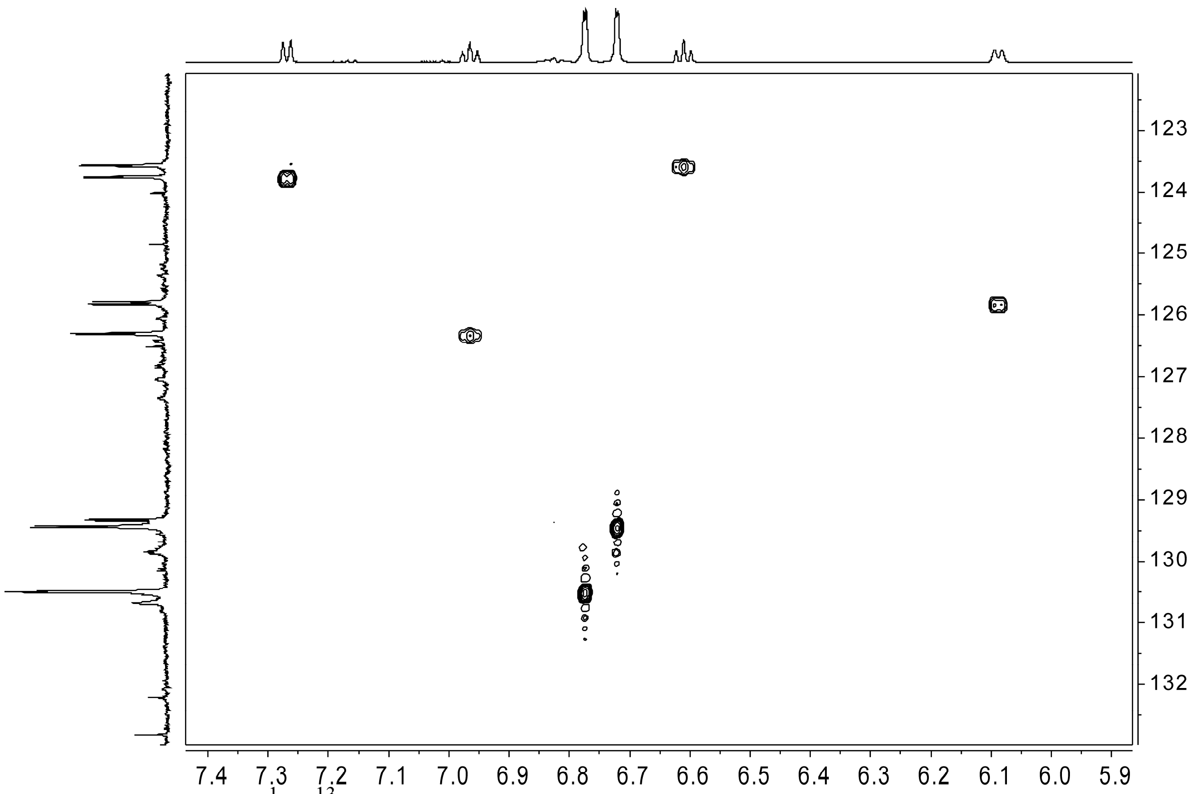

Figure S9: ^{1}H, ^{13}C GHSQC (600/151 MHz, 299 K, CD_2Cl_2) of compound **5**
[*Comment*: projection from ^{1}H NMR (600 MHz), $^{13}C\{^{1}H\}$ NMR (126 MHz)]

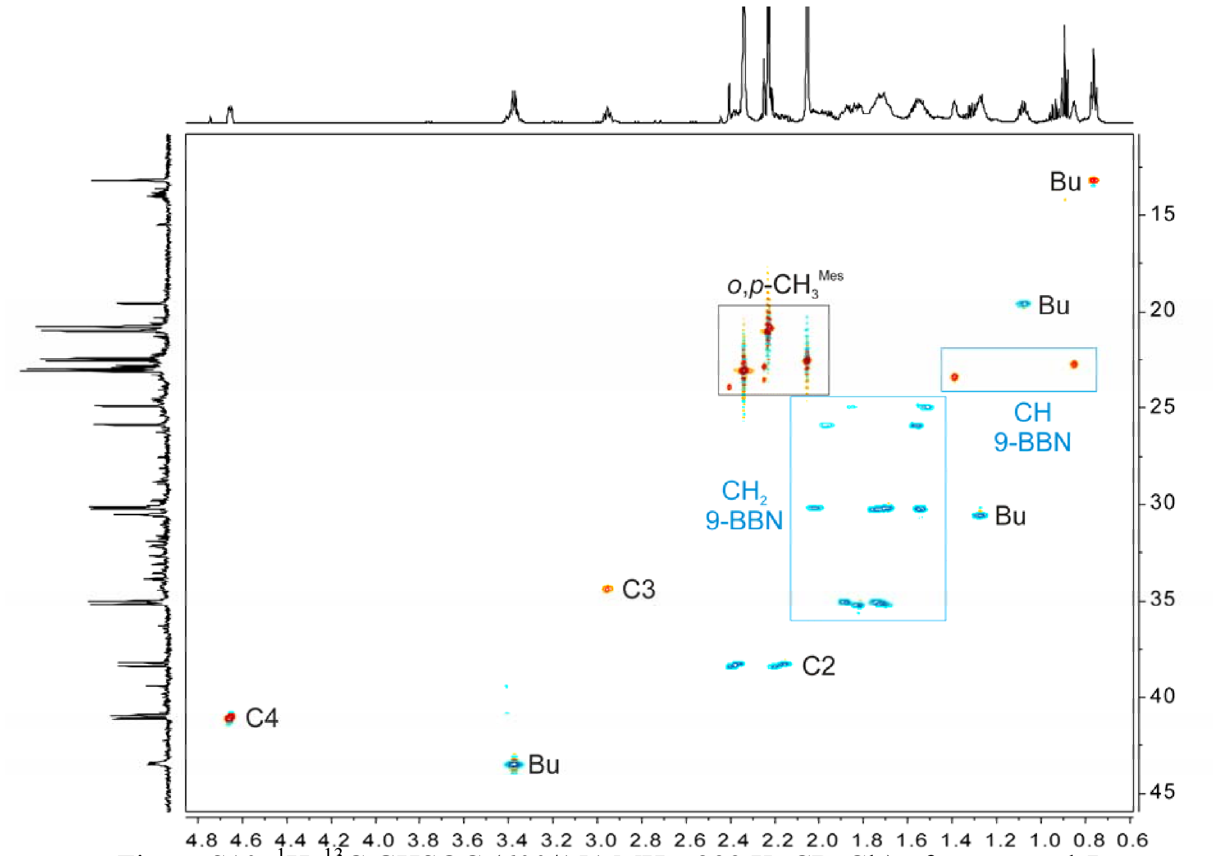

Figure S10: ^{1}H, ^{13}C GHSQC (600/151 MHz, 299 K, CD_2Cl_2) of compound **5**
red: CH and CH_3; blue: CH_2
[*Comment*: projection from ^{1}H NMR (600 MHz), $^{13}C\{^{1}H\}$ NMR (126 MHz)]

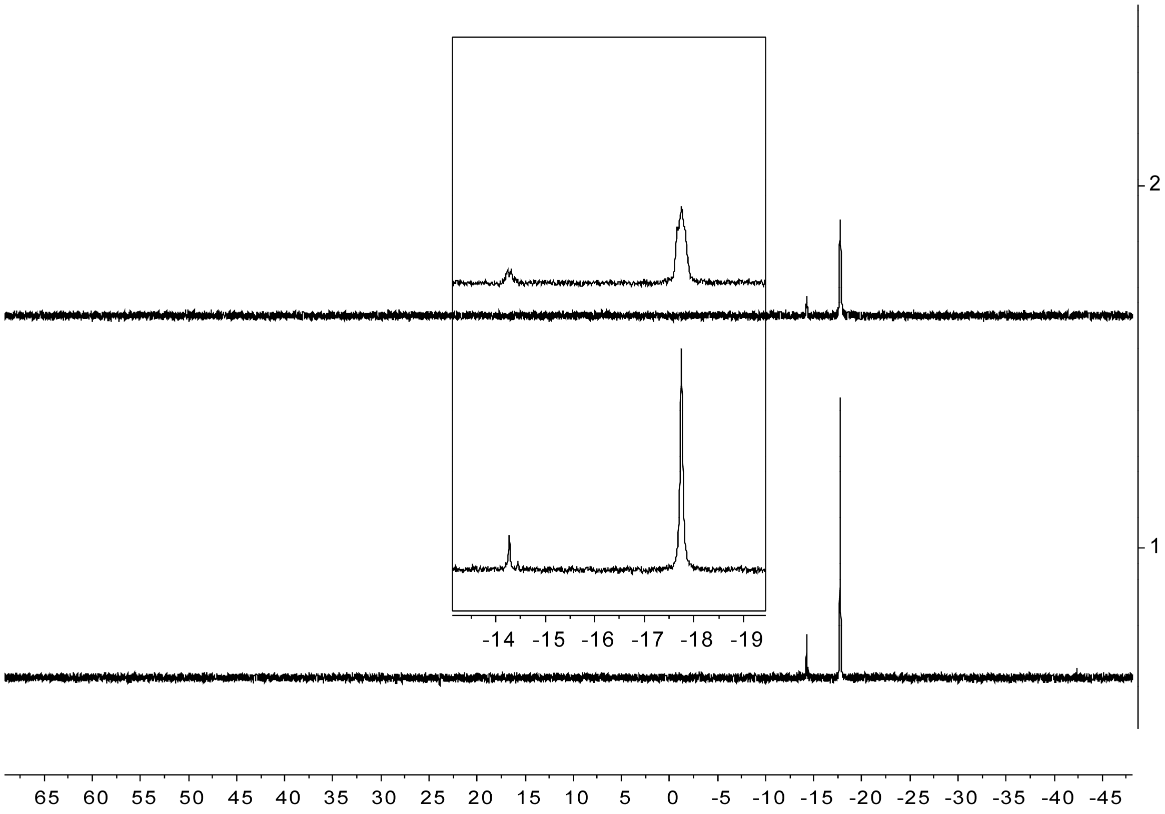

Figure S11: (1) $^{31}P\{^{1}H\}$ and (2) ^{31}P NMR (243 MHz, 299 K, CD_2Cl_2) of compound **5**

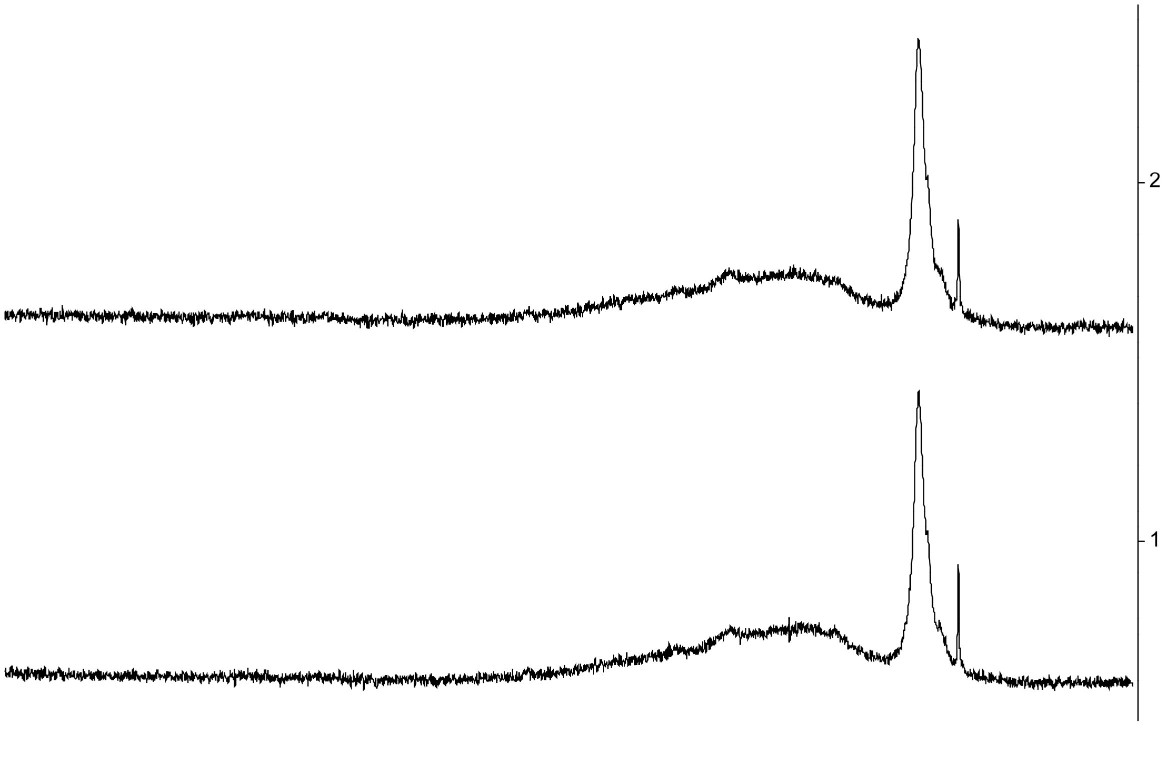

Figure S12: (1) $^{11}B\{^{1}H\}$ and (2) ^{11}B NMR (192 MHz, 299 K, CD_2Cl_2) of compound **5**

Figure S13: **X-ray crystal structure analysis of compound 5**. formula $C_{40}H_{53}BNP$, M = 589.61, colourless crystal, 0.15 x 0.10 x 0.05 mm, a = 14.6590(9), b = 12.4208(4), c = 20.7836(9) Å, β = 108.784(3) °, V = 3582.7(3) Å^3, ρ_{calc} = 1.093 gcm^{-3}, μ = 0.863 mm^{-1}, empirical absorption correction (0.881 ≤ T ≤ 0.958), Z = 4, monoclinic, space group $P2_1/c$ (No. 14), λ = 1.54178 Å, T = 223(2) K, ω and φ scans, 28433 reflections collected (±h, ±k, ±l), [(sinθ)/λ] = 0.60 Å^{-1}, 6234 independent (R_{int} = 0.066) and 4372 observed reflections [I>2σ(I)], 422 refined parameters, R = 0.054, wR^2 = 0.146, max. (min.) residual electron density 0.20 (-0.23) e.Å^{-3}, hydrogen atoms calculated and refined as riding atoms.

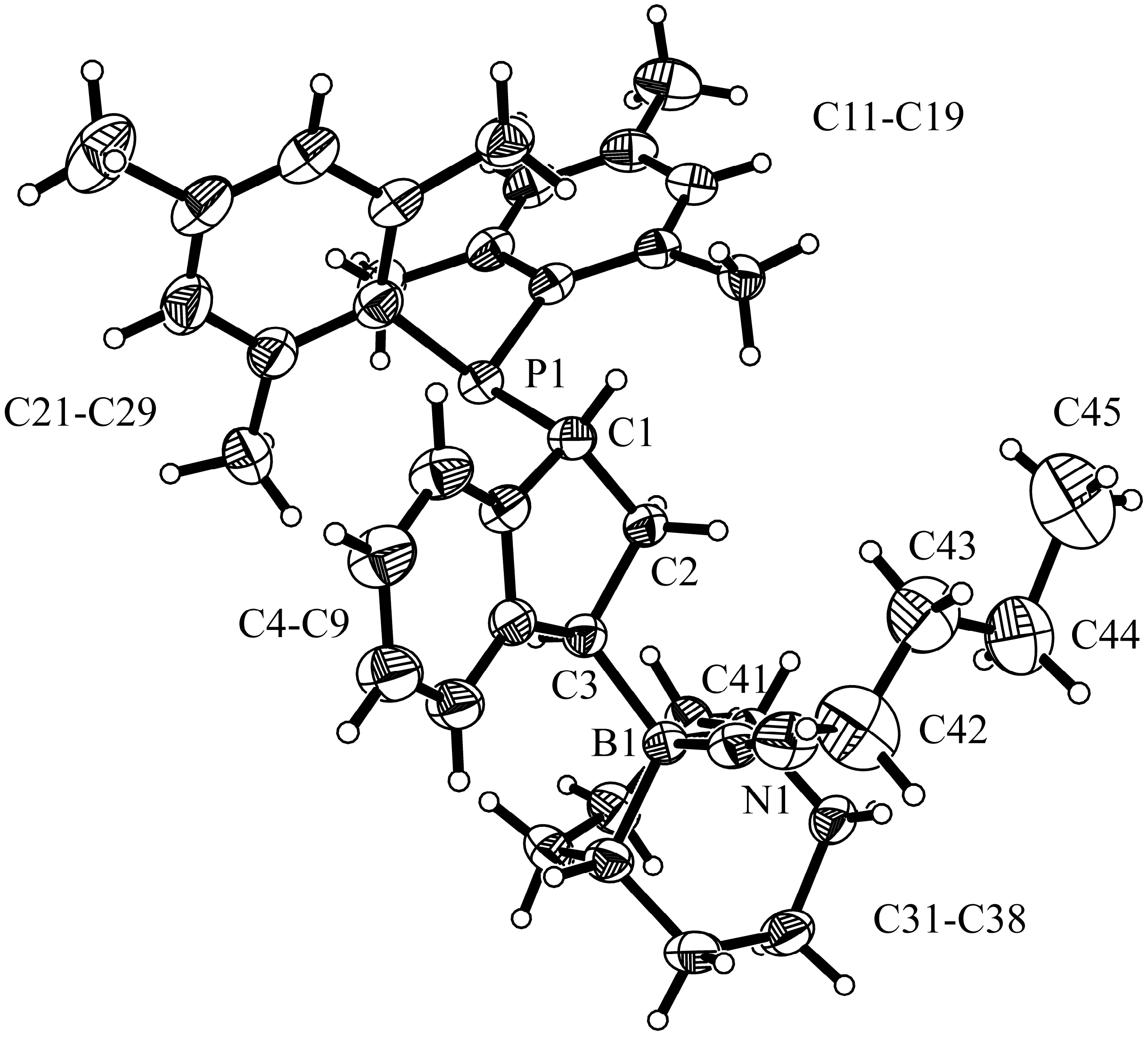

Compound 8

1st Experiment:

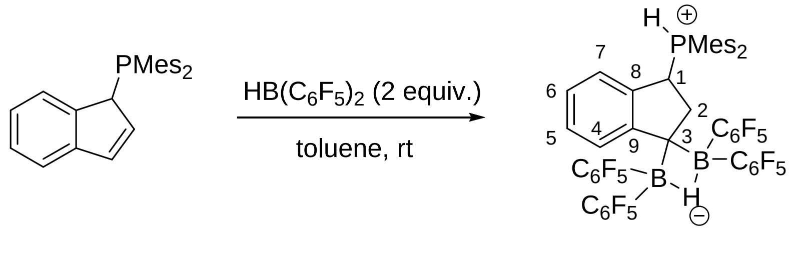

Scheme S4

Compound **4a** (38.4 mg, 0.1 mmol) was added at r.t. to a solution of $HB(C_6F_5)_2$ (69.2 mg, 0.2 mmol) in toluene (1 mL). Then the reaction mixture was stirred for 1 h. The obtained yellow solution was layered with pentane and stored in the freezer at -32 °C for 2 days until a white, amorphous powder precipitated. The liquid was removed and the solid was washed with pentane (3 x 2 mL). Then the solid was dried in vacuo to give compound **8** (60.2 mg, 56 %) as a white powder. **Anal.** calc. for $C_{51}H_{31}B_2F_{20}P_1$: C, 56.91; H, 2.90. Found: C, 56.11; H 2.82,

^{1}H NMR (500 MHz, 299 K, CD_2Cl_2): δ = 7.62 (dd, $^1J_{PH}$ = 477.5 Hz, $^2J_{HH}$ = 10.7 Hz, 1H, PH), 7.17 (d, $^4J_{PH}$ = 4.2 Hz, 2H, *m*-Mes^a), 7.10 (d, $^4J_{PH}$ = 4.3 Hz, 2H, *m*-Mes^b), 6.77 (m, 1H, 6-H), 6.72 (m, 1H, 5-H), 6.65 (m, 1H, 7-H), 5.76 (m, 1H, 4-H), 5.38 (m, 1H, 1-H), 3.30 (br, 1H, B-H-B), 2.71, 2.53 (each m, each 1H, 2-H), 2.52 (s, 6H, *o*-$CH_3^{Mes,b}$), 2.45 (br s, 6H, *o*-$CH_3^{Mes,a}$), 2.43 (s, 3H, *p*-$CH_3^{Mes,a}$), 2.35 (s, 3H, *p*-$CH_3^{Mes,b}$).

^{13}C{^{1}H} NMR (126 MHz, 299 K, CD_2Cl_2): δ = 156.3 (dm, $^3J_{PC}$ = 8.1 Hz, C9), 146.9 (d, J = 2.8 Hz, *p*-Mes^a), 146.4 (d, J = 2.7 Hz, *p*-Mes^b), 144.6 (d, $^2J_{PC}$ = 9.5 Hz, *o*-Mes^a), 143.7 (d, $^2J_{PC}$ = 9.2 Hz, *o*-Mes^b), 135.1 (d, $^2J_{PC}$ = 2.9 Hz, C8), 132.7 (d, $^3J_{PC}$ = 9.5 Hz, *m*-Mes^a), 132.1 (d, $^3J_{PC}$ = 10.6 Hz, *m*-Mes^b), 127.2 (d, J = 2.8 Hz, C5), 124.2 (br m, C4), 123.2 (C6), 123.1 (d, $^3J_{PC}$ = 4.9 Hz, C7), 112.5 (d, $^1J_{PC}$ = 74.9 Hz, *i*-Mes^a), 112.4 (d, $^1J_{PC}$ = 75.2 Hz, *i*-Mes^b), 40.1 (dm, $^1J_{PC}$ = 42.6 Hz, C1), 38.7 (C2), 28.4 (br, C3), 22.43 (br d, $^3J_{PC}$ = 6.3 Hz, *o*-$CH_3^{Mes,a}$), 22.36 (d, $^3J_{PC}$ = 7.0 Hz, *o*-$CH_3^{Mes,b}$), 21.5 (d, J = 1.0 Hz, *p*-$CH_3^{Mes,a}$), 21.3 (d, J = 0.9 Hz, *p*-$CH_3^{Mes,b}$), [C_6F_5 not listed].

^{31}P{^{1}H} NMR (202 MHz, 299 K, CD_2Cl_2): δ = –2.0 ($\nu_{1/2}$ ~ 10 Hz).

^{31}P NMR (202 MHz, 299 K, CD_2Cl_2): δ = –2.0 (dm, $^1J_{PH}$ ~ 480 Hz).

^{11}B{^{1}H} NMR (160 MHz, 299 K, CD_2Cl_2): δ = –16.2 ($\nu_{1/2}$ ~ 600 Hz).

^{11}B NMR (160 MHz, 299 K, CD_2Cl_2): δ = –16.2 ($\nu_{1/2}$ ~ 600 Hz).

^{19}F NMR (470 MHz, 299K, CD_2Cl_2): δ = –125.2 (m, 1F), –127.2 (m, 1F), –128.4 (br, 2F), –129.3 (br, 2F), –130.7 (m, 1F), –131.5 (m, 1F)(*o*-C_6F_5), –158.75 (t, $^3J_{FF}$ = 19.8), –158.84 (t,

$^{3}J_{FF}$ = 19.8), −159.5 (t, $^{3}J_{FF}$ = 19.6), −160.0 (t, $^{3}J_{FF}$ = 20.1)(each 1F, *p*-C_6F_5), −164.7, −165.0 (Σ6F), −165.7 (1F), −166.3 (1F)(each m, *m*-C_6F_5).

^{1}H,^{1}H GCOSY (500/500 MHz, 299 K, CD_2Cl_2)[selected traces]**:** δ^{1}H/δ^{1}H = 7.17 / 2.43 (*m*-Mes^{a} / *p*-$CH_3^{Mes,a}$), 7.10 / 2.52, 2.35 (*m*-Mes^{b} / *o*-$CH_3^{Mes,b}$, *p*-$CH_3^{Mes,b}$), 5.38 / 7.62, 2.71, 2.53 (1-H / PH, 2-H, 2-H).

^{1}H{^{1}H} TOCSY (500 MHz, 299 K, CD_2Cl_2)[selected experiment]**:** δ$^{1}H_{irr}$/δ$^{1}H_{res}$ = 5.76 / 6.77, 6.72, 6.65 (4-H / 6-H, 5-H, 7-H).

^{1}H{^{1}H} 1D-NOESY (500 MHz, 299 K, CD_2Cl_2)[selected experiment]**:** δ$^{1}H_{irr}$/δ$^{1}H_{res}$ = 7.17 / 2.45, 2.43 (*m*-Mes^{a} / *o*-$CH_3^{Mes,a}$, *p*-$CH_3^{Mes,a}$).

^{1}H,^{13}C GHSQC (500/126 MHz, 299 K, CD_2Cl_2): δ^{1}H/δ^{13}C = 7.17 / 132.7 (*m*-Mes^{a}), 7.10 / 132.1 (*m*-Mes^{b}), 6.77 / 123.2 (C6), 6.72 / 127.2 (C5), 6.65 / 123.1 (C7), 5.76 / 124.2 (C4), 5.38 / 40.1 (C1), 2.71, 2.53 / 38.7 (C2), 2.52 / 22.36 (*o*-$CH_3^{Mes,b}$), 2.45 / 22.43 (*o*-$CH_3^{Mes,a}$), 2.43 / 21.5 (*p*-$CH_3^{Mes,a}$), 2.35 / 21.3 (*p*-$CH_3^{Mes,b}$).

^{1}H,^{13}C GHMBC (500/126 MHz, 299 K, CD_2Cl_2)[selected traces]: δ^{1}H/δ^{13}C = 7.62 / 144.6, 143.7, 112.5, 112.4, 40.1 (PH / *o*-Mes^{a}, *o*-Mes^{b}, *i*-Mes^{a}, *i*-Mes^{b}, C1), 7.17 / 132.7, 112.5, 22.43, 21.5 (*m*-Mes^{a} / *m*-Mes^{a}, *i*-Mes^{a}, *o*-$CH_3^{Mes,a}$, *p*-$CH_3^{Mes,a}$), 7.10 / 132.1, 112.4, 22.36, 21.3 (*m*-Mes^{b} / *m*-Mes^{b}, *i*-Mes^{b}, *o*-$CH_3^{Mes,b}$, *p*-$CH_3^{Mes,b}$), 6.65 / 156.3, 127.2, 40.1 (7-H / C9, C5, C1), 5.76 / 135.1, 123.2 (4-H / C8, C6), 2.52 / 143.7, 132.1, 112.4 (*o*-$CH_3^{Mes,b}$ / *o*-Mes^{b}, *m*-Mes^{b}, *i*-Mes^{b}), 2.43 / 146.9, 132.7 (*p*-$CH_3^{Mes,a}$ / *p*-Mes^{a}, *m*-Mes^{a}), 2.35 / 146.4, 132.1 (*p*-$CH_3^{Mes,b}$ / *p*-Mes^{b}, *m*-Mes^{b}).

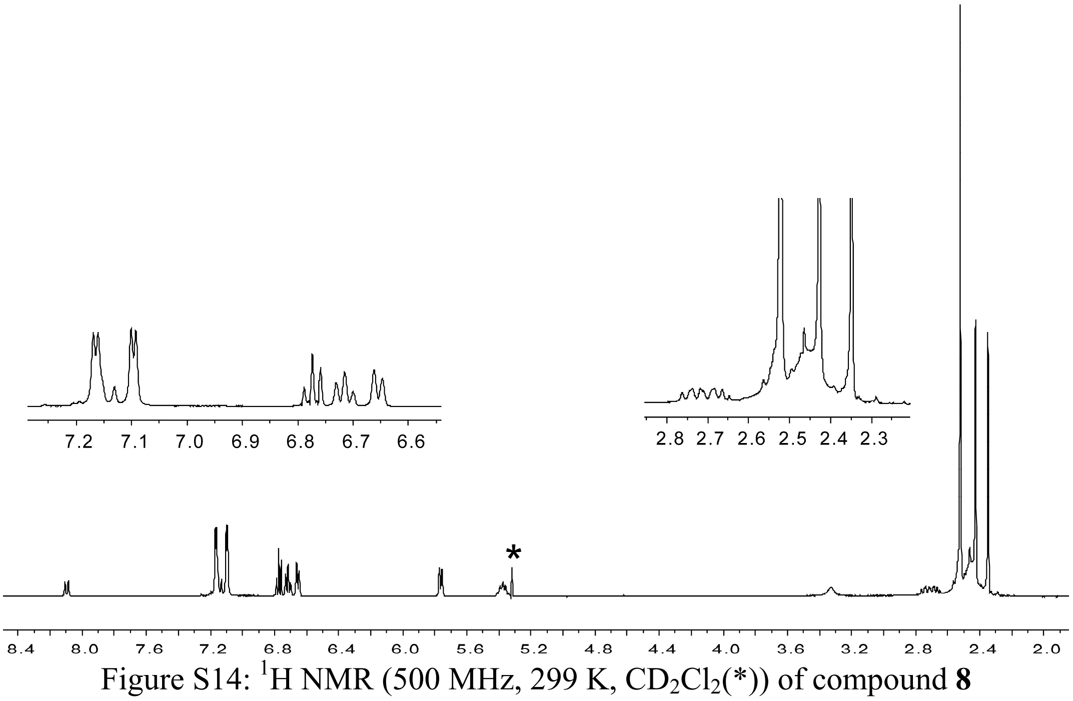

Figure S14: ^{1}H NMR (500 MHz, 299 K, CD_2Cl_2(*)) of compound **8**

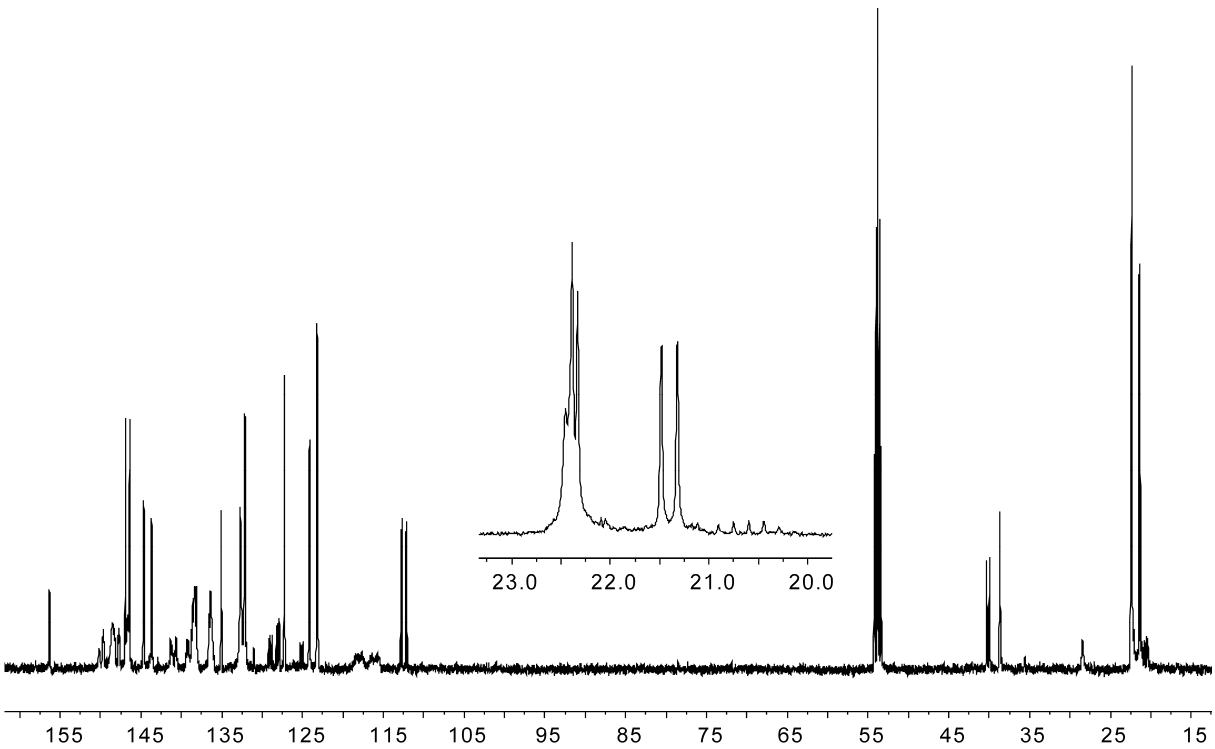

Figure S15: $^{13}C\{^{1}H\}$ NMR (126 MHz, 299 K, CD_2Cl_2) of compound **8** (traces of C_7D_8)

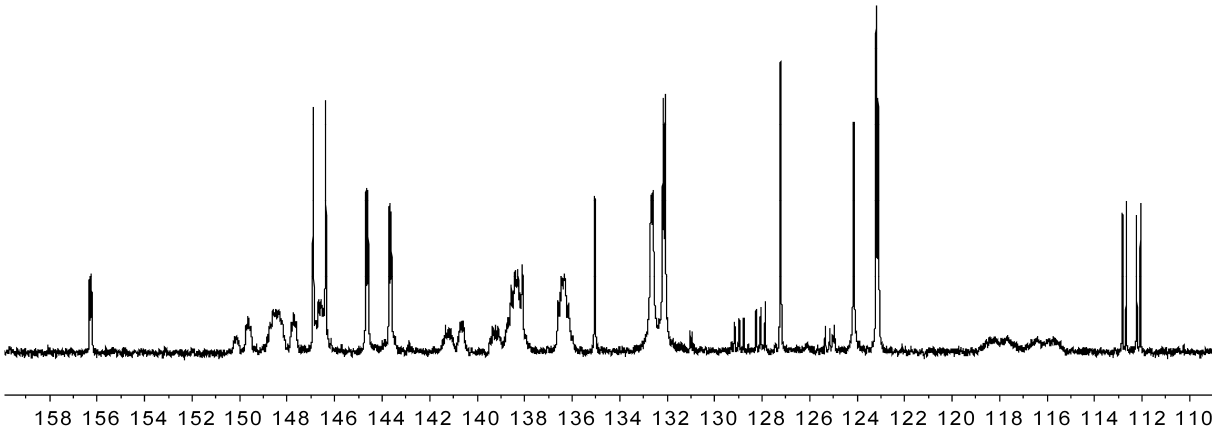

Figure S16: $^{13}C\{^{1}H\}$ NMR (126 MHz, 299 K, CD_2Cl_2) of compound **8** (traces of C_7D_8)

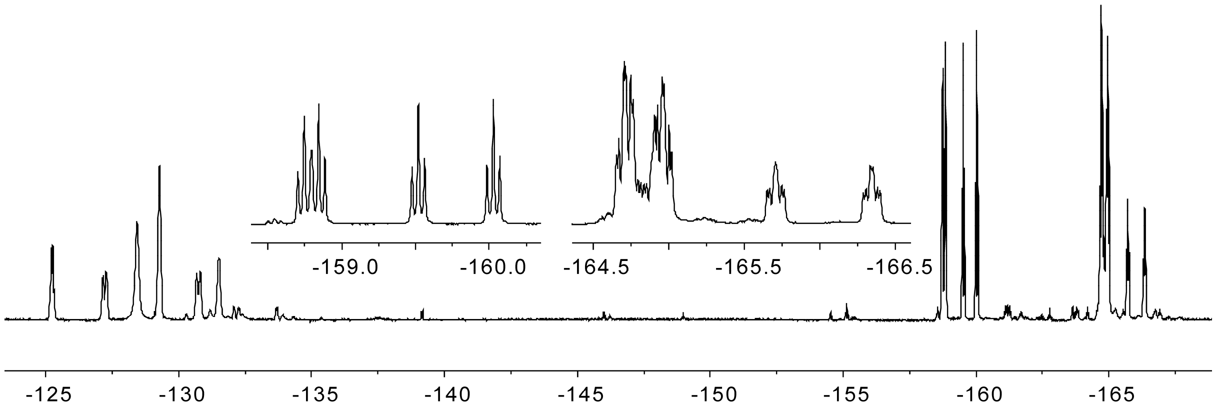

Figure S17: ^{19}F NMR (470 MHz, 299 K, CD_2Cl_2) of compound **8**

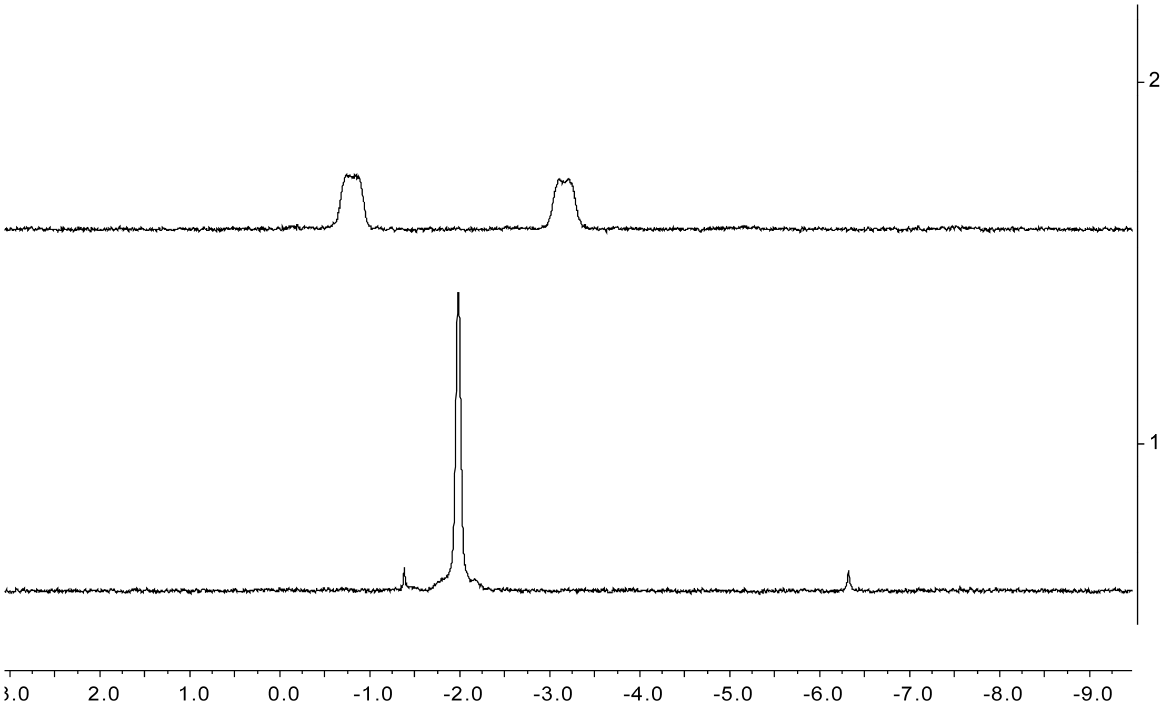

Figure S18: (1) $^{31}P\{^{1}H\}$ and (2) ^{31}P NMR (202 MHz, 299 K, CD_2Cl_2) of compound **8**

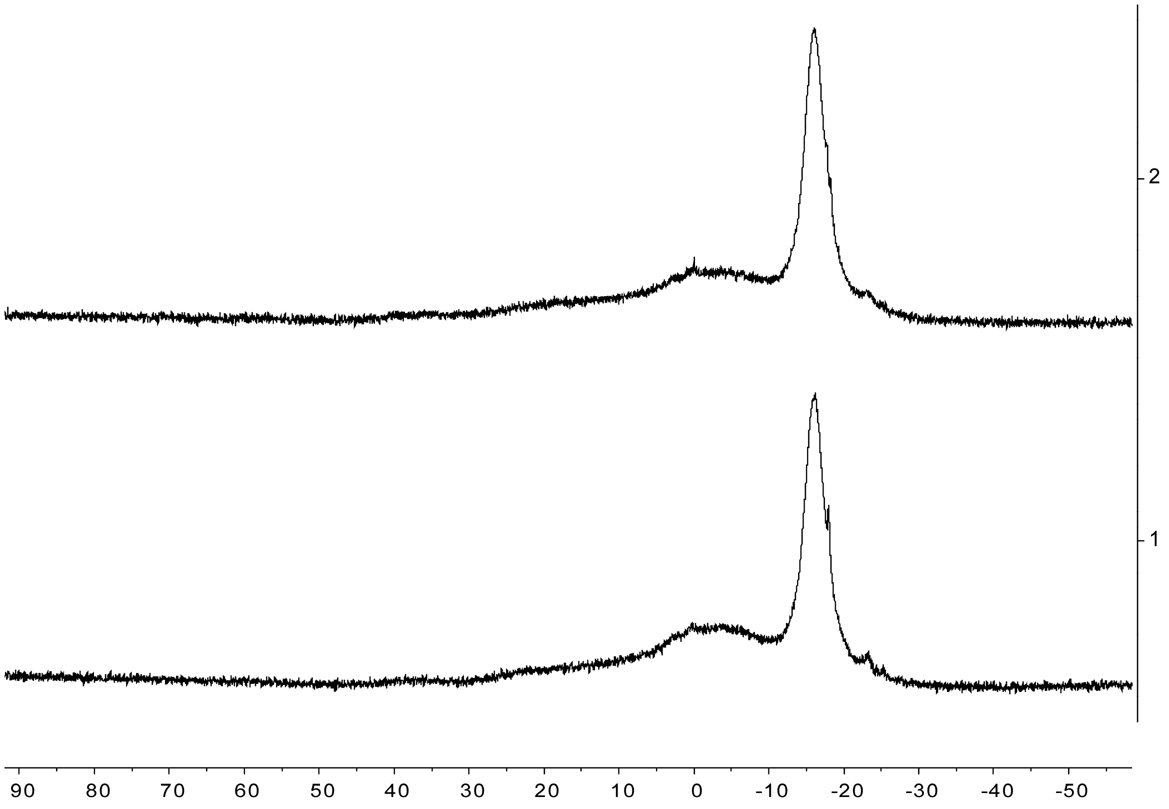

Figure S19: (1) $^{11}B\{^{1}H\}$ and (2) ^{11}B ´NMR (160 MHz, 299 K, CD_2Cl_2) of compound **8**

2nd Experiment:

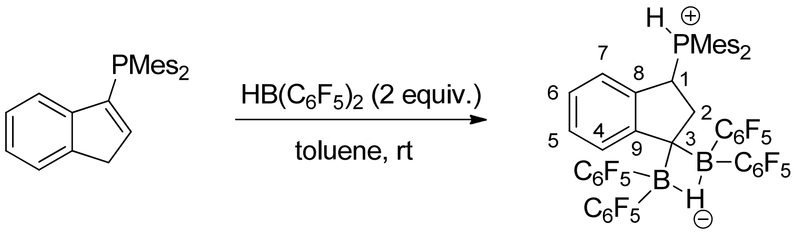

Scheme S5

Compound **4b** (38.4 mg, 0.1 mmol) was added at r.t. to a solution of $HB(C_6F_5)_2$ (69.2 mg, 0.2 mmol) in toluene (1 mL). Then the reaction mixture was stirred for 1 h. The obtained yellow solution was layered with pentane and stored in the freezer at -32 °C for 2 days until a white, amorphous powder precipitated. The liquid was removed and the solid was dried in vacuo to give compound **8** (65 mg, 60 %) as a white powder. [the NMR data of compound **8** are consistent with those listed in the 1st experiment]

3rd Experiment:

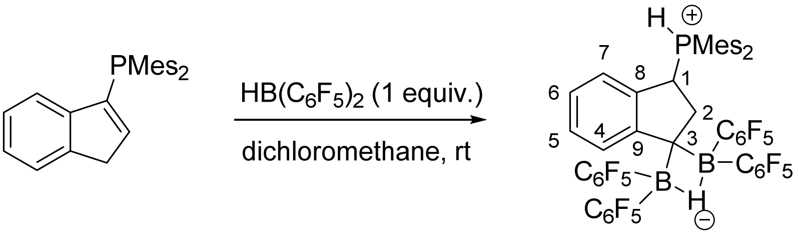

Scheme S6

NMR Scale: Compound **4b** (38.4 mg, 0.1 mmol) was added at r.t. to a solution of $HB(C_6F_5)_2$ (34.6 mg, 0.1 mmol) in CD_2Cl_2 (1 mL). Then the reaction mixture was characterized by NMR experiments:

^{31}P{^{1}H} NMR (121 MHz, 299 K, CD_2Cl_2): δ = -2.1 [dm, $^{1}J_{PH}$ = 478.0 Hz, **8** (36 mol%)], -11.8 (br d, $^{1}J_{PH}$ ~ 478.4 Hz, unidentified compound (4 mol%)], -39.4 (br d, $^{1}J_{PH}$ ~ 400 Hz, unidentified compound (19 mol%)], -42.4 [$\nu_{1/2}$ ~ 3 Hz, **4b** (41 mol%)].

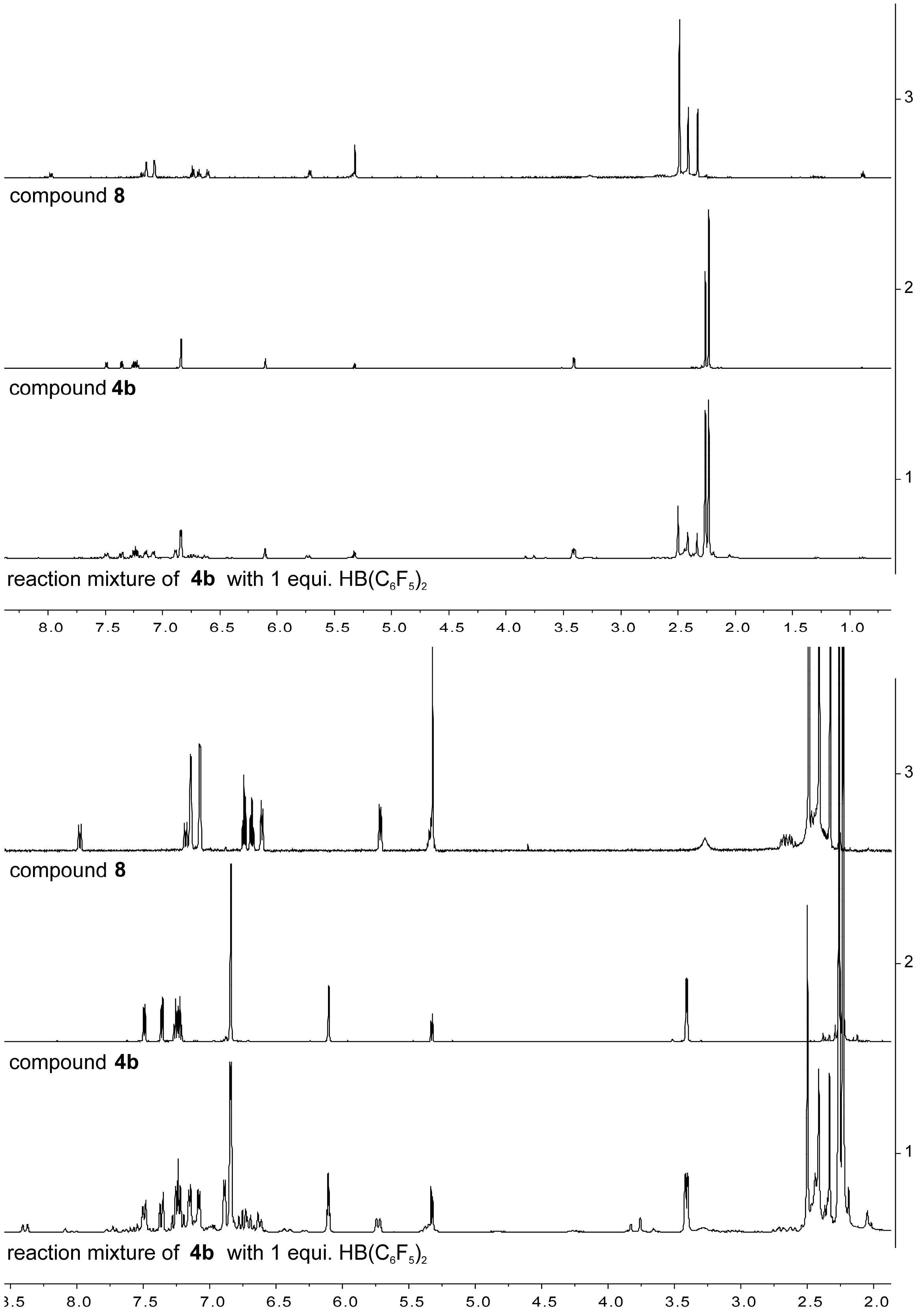

Figure S20: (1) ^{1}H NMR (600 MHz, 299 K, CD_2Cl_2) spectrum of isolated compound **8** (2) ^{1}H NMR (600 MHz, 299 K, CD_2Cl_2) spectrum of isolated compound **4b**. (3) ^{1}H NMR (300 MHz, 299 K, CD_2Cl_2) spectrum of the reaction of compound **4b** with 1 equivalent HBC_6F_5 in CD_2Cl_2

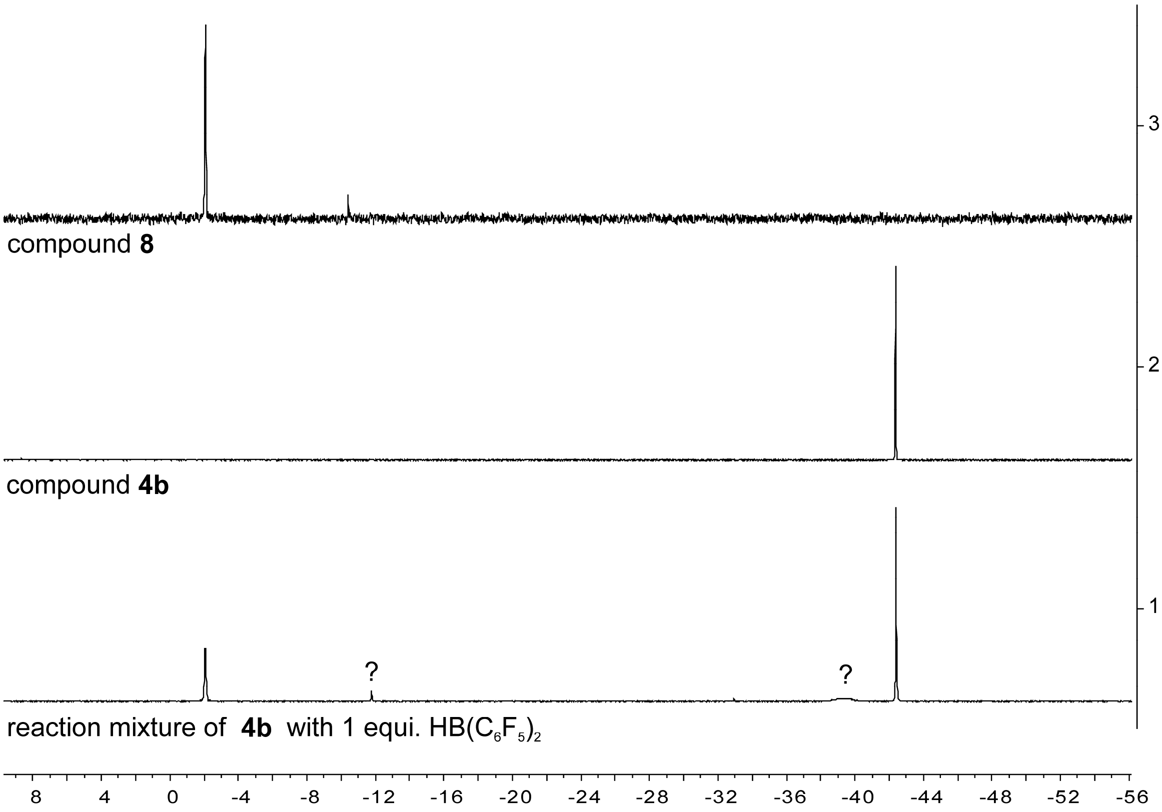

Figure S21: (1) $^{31}P\{^{1}H\}$ NMR (121 MHz, 299 K, CD_2Cl_2) spectrum of isolated compound **8**. (2) $^{31}P\{^{1}H\}$ NMR (243 MHz, 299 K, CD_2Cl_2) spectrum of isolated compound **4b**. (3) $^{31}P\{^{1}H\}$ NMR (121 MHz, 299 K, CD_2Cl_2) spectrum of the reaction of compound **4b** with 1 equivalent HBC_6F_5 in CD_2Cl_2

Compound 9.

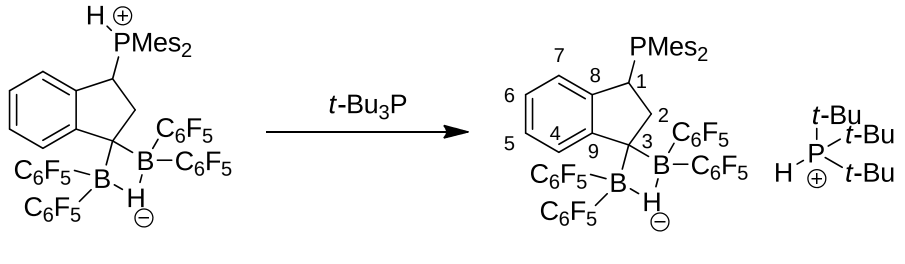

Scheme S7

t-Bu_3P (20.2 mg, 0.1 mmol) was added at r.t. to a solution of compound **8** (107.6 mg, 0.1 mmol) in CD_2Cl_2 (1 mL). Then the reaction mixture was stirred for 1 h. Crystals of compound **9** suitable for the X-ray crystal structure analysis were obtained from the reaction mixture by slow evaporation of CD_2Cl_2 at -36 °C.

Figure S22: **X-ray crystal structure analysis of compound 9**. formula $C_{63}H_{58}B_2F_{20}P_2$, M = 1278.65, pale yellow crystal, 0.54 x 0.36 x 0.22 mm, a = 14.7651(4), b = 17.3362(4), c = 23.4491(3) Å, V = 6002.3(2) Å^3, ρ_{calc} = 1.415 gcm^{-3}, μ = 1.566 mm^{-1}, empirical absorption correction (0.485 ≤ T ≤ 0.724), Z = 4, orthorhombic, space group $P2_12_12_1$ (No. 19), λ = 1.54178 Å, T = 223(2) K, ω and φ scans, 29674 reflections collected (±h, ±k, ±l), [(sinθ)/λ] = 0.60 Å^{-1}, 10182 independent (R_{int} = 0.035) and 9853 observed reflections [I>2σ(I)], 807 refined parameters, R = 0.033, wR^2 = 0.081, max. (min.) residual electron density 0.23 (-0.18) e.Å^{-3}, the hydrogens at P1 and B1-B2 atoms were refined freely; others were calculated and refined as riding atoms. Flack parameter: -0.01(1).

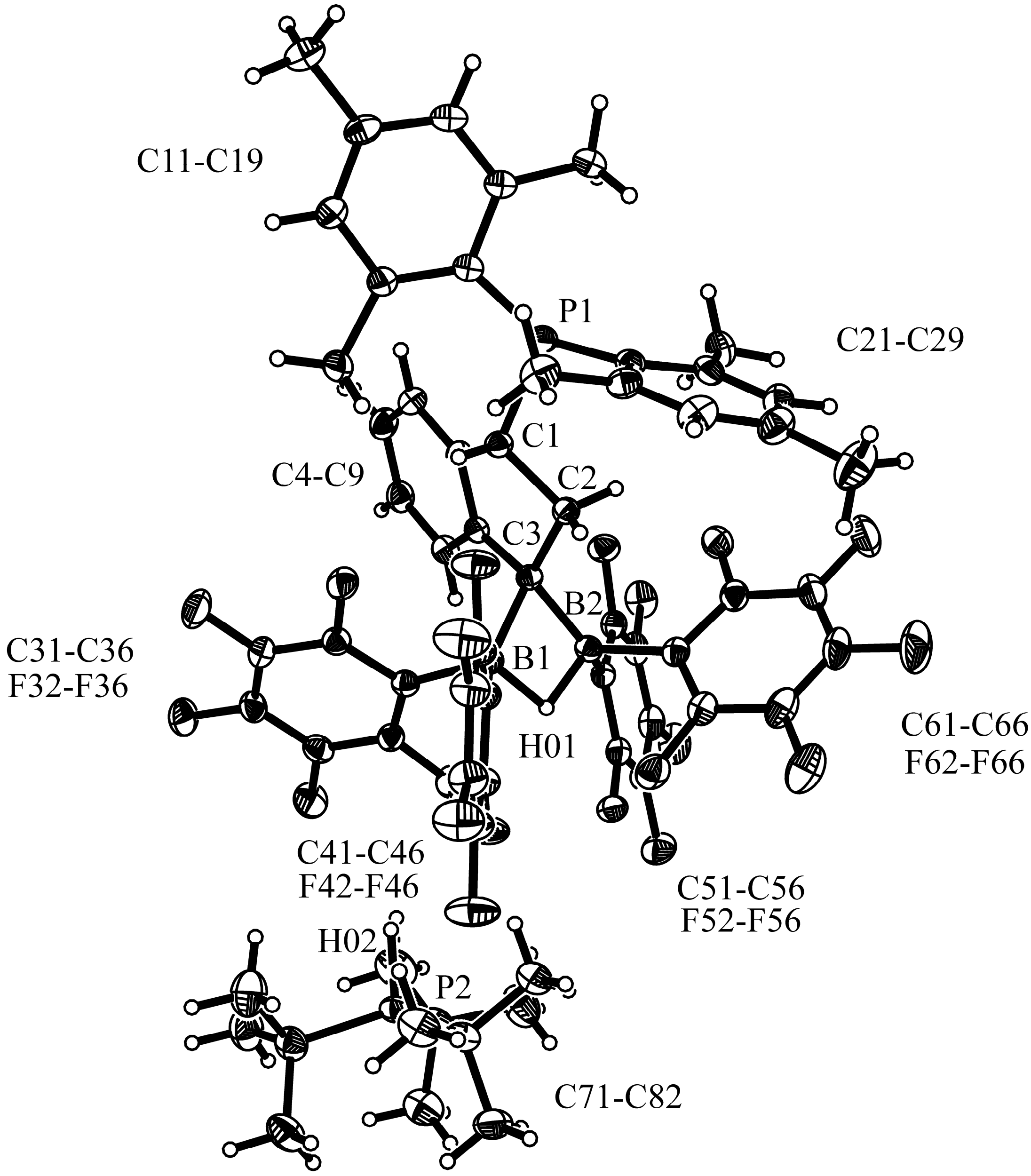

Characterization of compound 14.

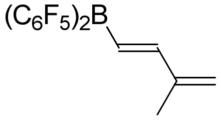

Scheme S8

NMR Scale: Bis(pentafluorophenyl)borane (34.6 mg, 0.1 mmol, 1 eq) and 2- methylbut-1-en-3-yne (7.2 mg, 1.1 mmol, 1.1 eq) were suspended in C_6D_6 (0.5 mL) and stirred for 2 h at room temperature. Then the yellow reaction mixture was transferred into an NMR tube, which was sealed in an argon atmosphere immediately.

^{1}H NMR (500 MHz, 299 K, C_6D_6): δ = 7.21 (d, $^3J_{HH}$ = 17.4 Hz, 1H, =CH), 6.90 (d, $^3J_{HH}$ = 17.4 Hz, 1H, BCH), 5.14 (m, 1H, =CH_2^E), 5.11 (m, 1H, =CH_2^Z), 1.70 (m, 3H, CH_3).

^{13}C{^{1}H} NMR (126 MHz, 299 K, C_6D_6): δ = 165.2 (=CH), 147.6 (dm, $^1J_{FC}$ ~ 245 Hz, C_6F_5), 143.6 (=CMe), 143.2 (dm, $^1J_{FC}$ ~ 255 Hz, C_6F_5), 137.6 (dm, $^1J_{FC}$ ~ 250 Hz, C_6F_5), 132.8 (br, BCH), 129.0 (=CH_2), 114.0 (br, *i*-C_6F_5), 17.5 (CH_3).

^{11}B{^{1}H} NMR (160 MHz, 299 K, C_6D_6): δ = 58.8 ($\nu_{1/2}$ ~ 800 Hz).

^{19}F NMR (470 MHz, 299 K, C_6D_6): δ = -129.9 (m, 2F, *o*-C_6F_5), -148.4 (tt, $^3J_{FF}$ = 20.7 Hz, $^4J_{FF}$ = 4.0 Hz, 1F, *p*-C_6F_5), -161.3 (m, 2F, *m*-C_6F_5), [$\Delta\delta^{19}F_{m,p}$ = 12.9].

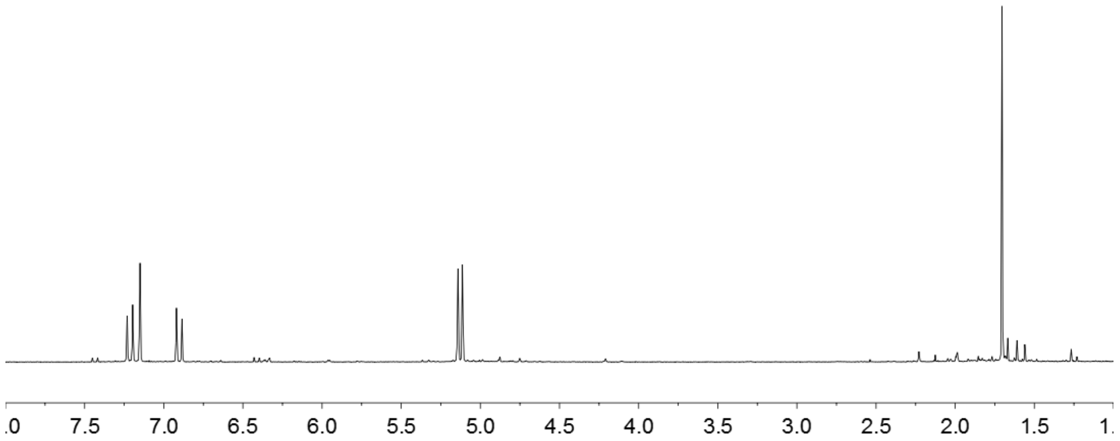

Figure S23: ^{1}H NMR (500 MHz, 299 K, C_6D_6) of compound **14**

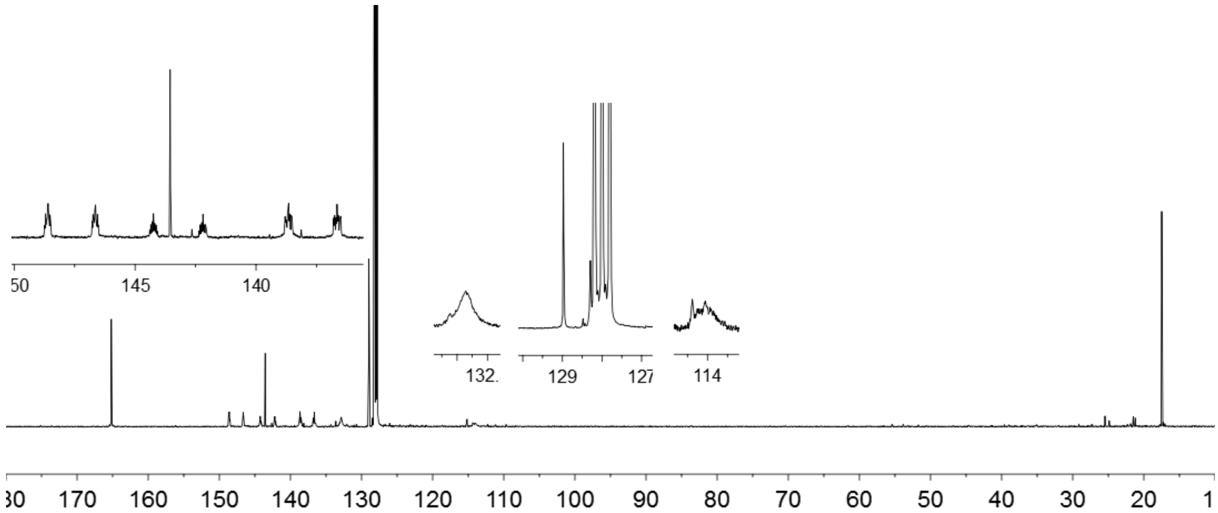

Figure S24: **^{13}C{^{1}H} NMR** (126 MHz, 299 K, CD_2Cl_2) of compound **14**

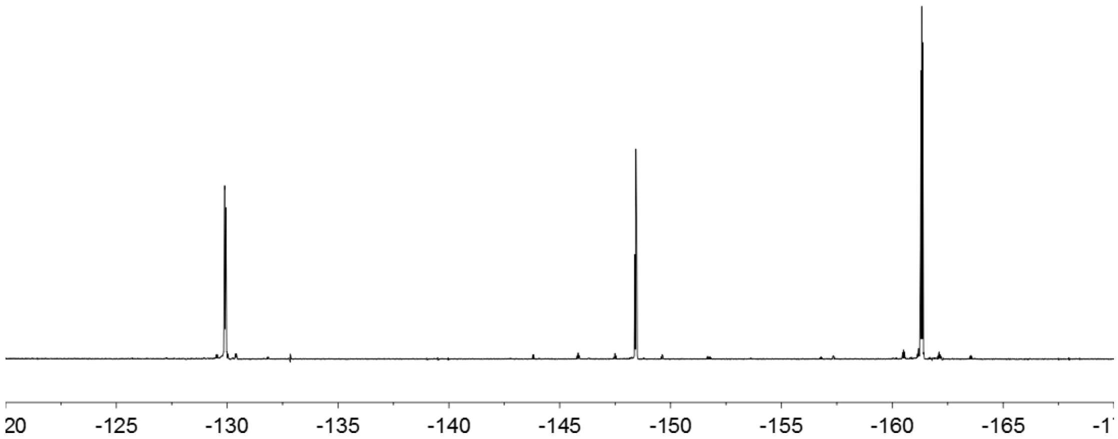

Figure S25: ^{19}F NMR (470 MHz, 299 K, C_6D_6) of compound **14**

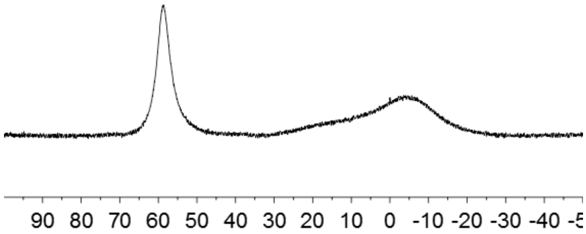

Figure S26: ^{11}B{^{1}H} NMR (160 MHz, 299 K, C_6D_6) of compound **14**

Synthesis of compound 17a.

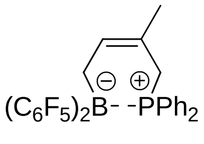

Scheme S9

Bis(pentafluorophenyl)borane (0.346 g, 1.0 mmol, 1 eq) and 2- methylbut-1-en-3-yne (0.072 g, 1.1 mmol, 1.1 eq) were suspended in toluene (5 mL) and stirred for 4 h at room temperature. Then diphenylphosphane (0.186 g, 1.0 mmol, 1 eq) in toluene (5 mL) was added and the reaction mixture was heated at 80 °C for 3 d. Subsequently all volatiles were removed in vacuo and the obtained residue was washed with cold pentane (3×1 mL). After drying in vacuo compound **17a** (0.442 g, 0.74 mmol, 74 %) was obtained as a white solid. Crystals suitable for the X-ray crystal structure analysis were obtained by slow diffusion of pentane to a dichloromethane solution of compound **17a** at -35 °C. **IR** (KBr): $\tilde{\upsilon}$ / cm^{-1} = 3069, 3027, 2963, 2930, 1642, 1518, 1457, 1378, 1285, 1100, 984, 742, 692. **M.p.**: 184 °C. **Anal. Calc.** for $C_{29}H_{18}BF_{10}P$: C: 58.22; H: 3.03. Found: C: 58.41; H: 2.82.

^{1}H NMR (500 MHz, 299 K, CD_2Cl_2): δ = 7.51 (m, 2H, *p*-Ph), 7.35 (m, 4H, *m*-Ph), 7.26 (m, 4H, *o*-Ph), 6.09 (dt, $^4J_{PH}$ = 5.4 Hz, $^3J_{HH}$ = 5.3 Hz, 1H, HC=), 2.98 (d, $^2J_{PH}$ = 13.3 Hz, 2H, PCH_2), 2.13 (dd, $^3J_{PH}$ = 23.0 Hz, $^3J_{HH}$ = 4.2 Hz, 2H, BCH_2), 1.76 (m, 3H, CH_3).

^{1}H{^{31}P} NMR (500 MHz, 299 K, CD_2Cl_2)[selected resonances]: δ = 6.09 (t, $^3J_{HH}$ = 5.3 Hz, 1H, HC=), 2.98 (s, 2H, PCH_2), 2.13 (d, $^3J_{HH}$ = 4.2 Hz, 2H, BCH_2).

^{13}C{^{1}H} NMR (126 MHz, 299 K, CD_2Cl_2): δ = 148.5 (dm, $^1J_{FC}$ ~ 240 Hz, C_6F_5), 139.7 (dm, $^1J_{FC}$ ~ 250 Hz, C_6F_5), 137.3 (dm, $^1J_{FC}$ ~ 250 Hz, C_6F_5), 132.8 (d, $^2J_{PC}$ = 7.9 Hz, *o*-Ph), 132.1 (d, $^3J_{PC}$ = 12.3 Hz, HC=), 132.0 (d, $^4J_{PC}$ = 2.8 Hz, *p*-Ph), 129.0 (d, $^3J_{PC}$ = 10.0 Hz, *m*-Ph), 127.4 (d, $^1J_{PC}$ = 53.5 Hz, *i*-Ph), 125.2 (d, $^2J_{PC}$ = 5.6 Hz, =CMe), 119.8 (br, *i*-C_6F_5), 27.7 (d, $^1J_{PC}$ = 35.5 Hz, PCH_2), 26.1 (d, $^3J_{PC}$ = 6.9 Hz, CH_3), 21.7 (br, BCH_2).

^{11}B{^{1}H} NMR (160 MHz, 299 K, CD_2Cl_2): δ = -13.6 (br d, $^1J_{PB}$ ~ 70 Hz).

^{31}P{^{1}H} NMR (202 MHz, 299 K, CD_2Cl_2): δ = 1.3 (1:1:1:1 q, partial relaxed $^1J_{PB}$ ~ 70 Hz).

^{19}F NMR (470 MHz, 299 K, CD_2Cl_2): δ = -129.2 (m, 2F, *o*-C_6F_5), -159.3 (t, $^3J_{FF}$ = 20.3 Hz, 1F, *p*-C_6F_5), -165.0 (m, 2F, *m*-C_6F_5), [$\Delta\delta^{19}F_{m,p}$= 5.7].

^{1}H, ^{1}H GCOSY (500 MHz / 500 MHz, 299 K, CD_2Cl_2)[selected traces]: δ ^{1}H / δ ^{1}H = 7.35 / 7.51, 7.26 (*m*-Ph / *p*-Ph, *o*-Ph), 6.09 / 2.98, 2.13, 1.76 (HC= / PCH_2, BCH_2, CH_3).

^{1}H, ^{13}C GHSQC (500 MHz / 126 MHz, 299 K, CD_2Cl_2): δ ^{1}H / δ ^{13}C = 7.51 / 132.0 (*p*-Ph), 7.35 / 129.0 (*m*-Ph), 7.26 / 132.8 (*o*-Ph), 6.09 / 132.1 (HC=), 2.98 / 27.7 (PCH_2), 2.13 / 21.7 (BCH_2), 1.76 / 26.1 (CH_3).

^{1}H, ^{13}C GHMBC (500 MHz / 126 MHz, 299 K, CD_2Cl_2)[selected traces]: δ ^{1}H / δ ^{13}C = 7.35 / 132.0, 128.9, 127.4 (*m*-Ph / *p*-, *m*-, *i*-Ph), 2.98 / 132.1, 127.4, 125.2, 26.1 (PCH_2 / HC=, *i*-Ph, =CMe, CH_3), 2.13 / 132.1, 125.2, 119.8 (BCH_2 / HC=, =CMe, *i*-C_6F_5), 1.76 / 132.1, 125.2, 27.7 (CH_3 / HC=, =CMe, PCH_2).

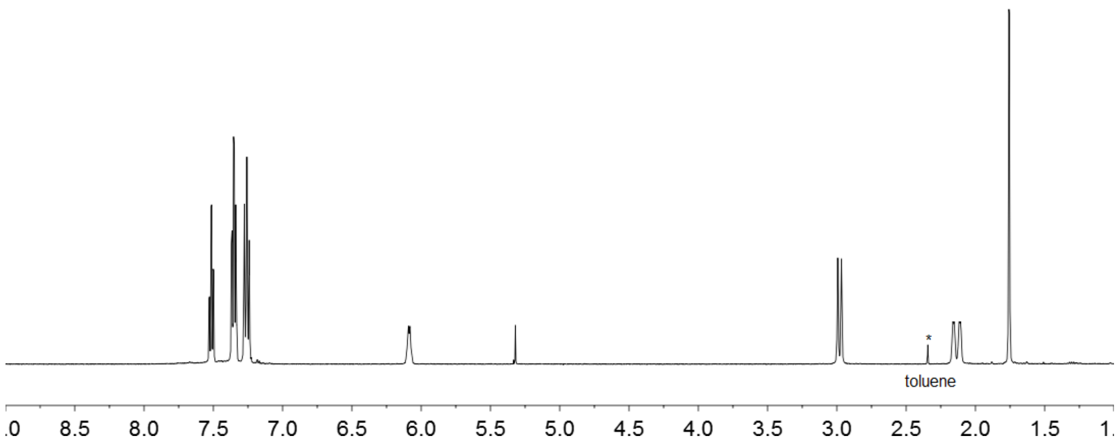

Figure S27: **^{1}H NMR** (500 MHz, 299 K, CD_2Cl_2) of compound **17a**

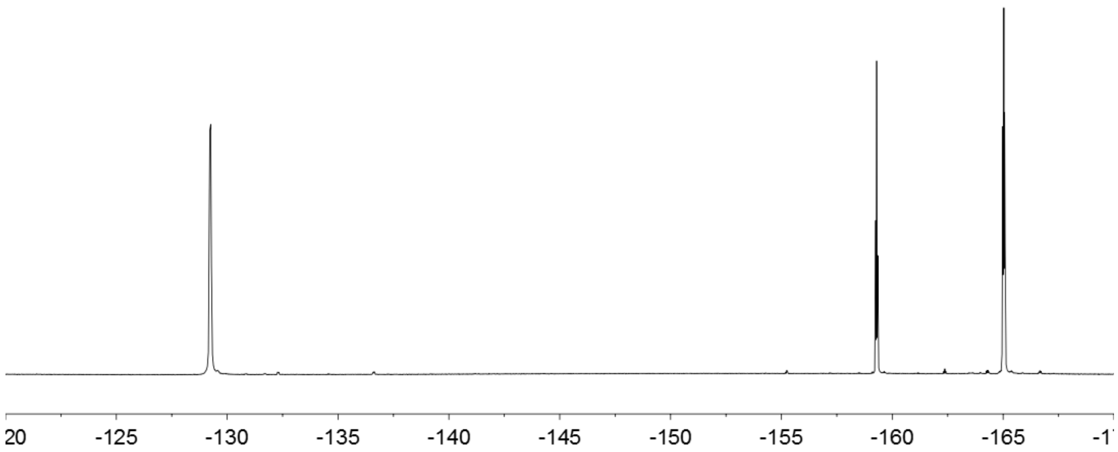

Figure S28: **^{19}F NMR** (470 MHz, 299 K, CD_2Cl_2) of compound **17a**

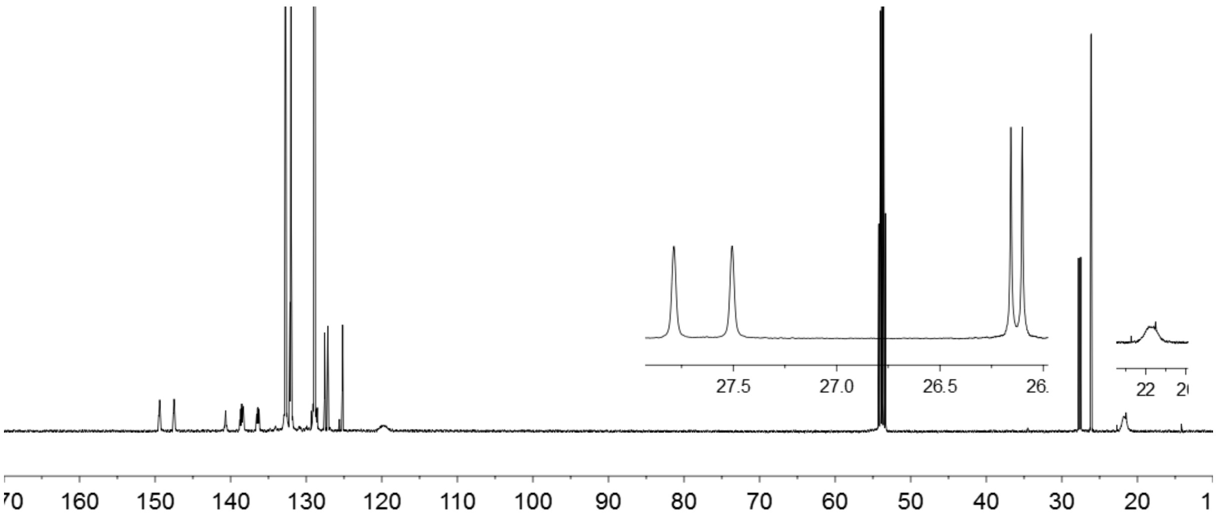

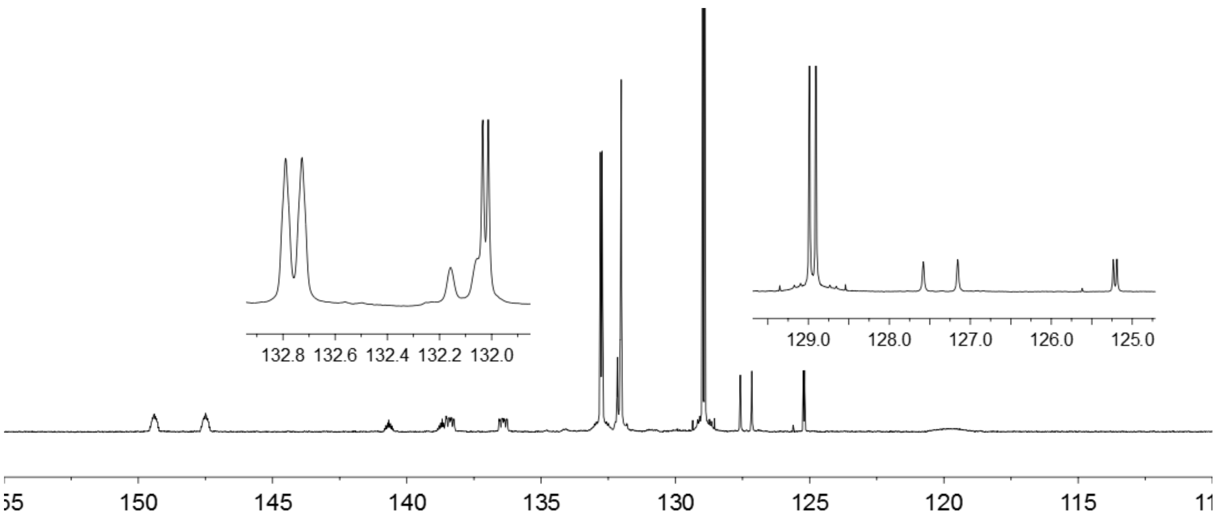

Figure S29: **$^{13}C\{^{1}H\}$ NMR** (126 MHz, 299 K, CD_2Cl_2) of compound **17a**

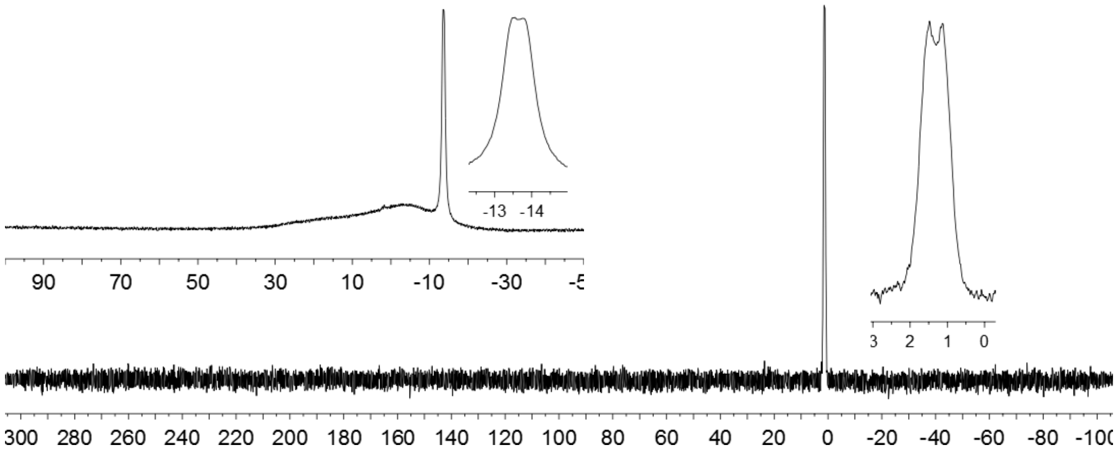

Figure S30: **$^{11}B\{^{1}H\}$ NMR** (160 MHz, 299 K, CD_2Cl_2) and **$^{31}P\{^{1}H\}$ NMR** (202 MHz, 299 K, CD_2Cl_2) of compound **17a**

Dynamic ^{19}F NMR:

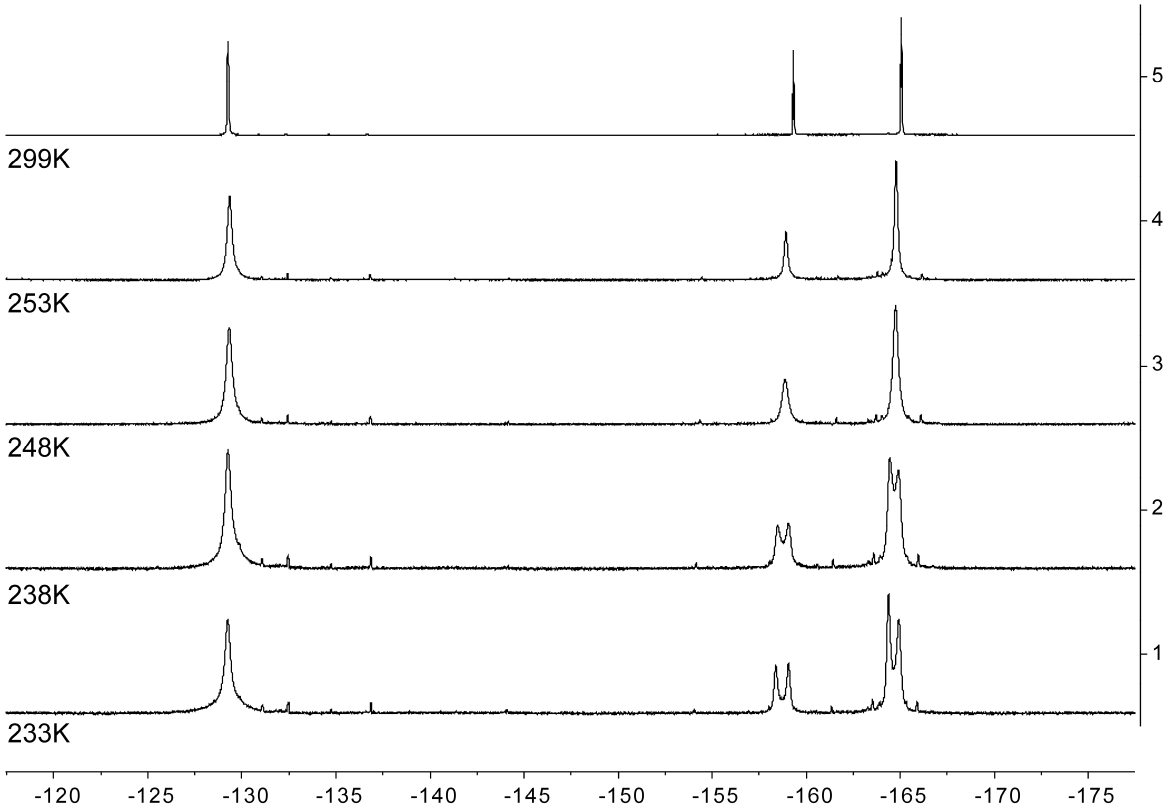

Figure S31: **^{19}F NMR** (564 MHz, CD_2Cl_2) of compound **17a**

$\Delta G^{\ddagger} = RT_c(22.96 + \ln(T_c/\delta\nu))$

T_c = coalescence temperature [K]: 243 K (^{19}F, *p*-BC_6F_5)

$\Delta\nu$ = chemical shift difference [Hz] (^{19}F, *p*-BC_6F_5, 233 K): 390 Hz

R = 8.314 J/(mol·K); 1 J = 0.239 cal

$\Delta G^{\ddagger}$[243K, $\delta\nu$(233 K) = 390 Hz] = 45430 J/mol = 10.9 ± 0.3 kcal/mol

Figure S32: **X-ray crystal structure analysis of compound 17a**. formula $C_{29}H_{18}BF_{10}P$, M = 598.21, colourless crystal, 0.35 x 0.15 x 0.13 mm, a = 10.0670(7), b = 11.7645(11), c = 13.4704(13) Å, α = 109.385(3), β = 97.336(5), γ = 114.390(5)°, V = 1303.9(2) Å^3, ρ_{calc} = 1.524 gcm^{-3}, μ = 1.767 mm^{-1}, empirical absorption correction (0.576 ≤ T ≤ 0.802), Z = 2, triclinic, space group $P\bar{1}$ (No. 2), λ = 1.54178 Å, T = 223(2) K, ω and φ scans, 14759 reflections collected ($\pm h$, $\pm k$, $\pm l$), [(sinθ)/λ] = 0.60 Å^{-1}, 4498 independent (R_{int} = 0.036) and 4284 observed reflections [I>2σ(I)], 371 refined parameters, R = 0.035, wR^2 = 0.094, max. (min.) residual electron density 0.30 (-0.23) e.Å^{-3}, hydrogen atoms were calculated and refined as riding atoms.

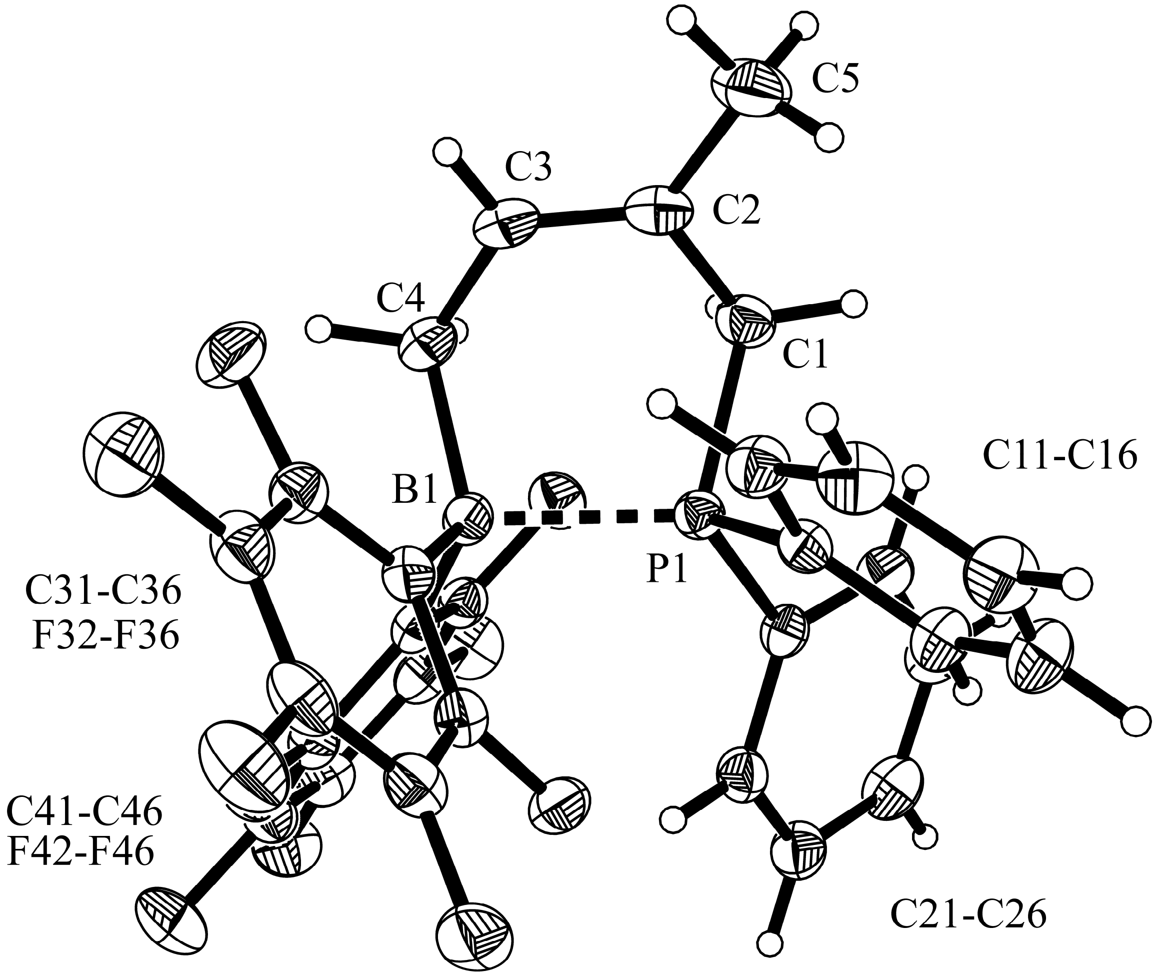

Generation of compound **17a**-D:

a) Preparation of $DPPh_2$: ^{n}BuLi (10 mL, 1.6 M in hexane, 16 mmol, 1eq) was added to diphenylphosphane (2.98 g, 16 mmol, 1 eq) in Et_2O (40 mL) at -78 °C. The solution was stirred for 1 h at -78 ^{o}C and 1 h at room temperature. Then D_2O (degassed, 1 mL) was added at 0°C, and the mixture was stirred vigorously at room temperature for 1 h. After H_2O (degassed, 10 mL) was added to the reaction mixture the organic phase was separated, washed by saturated aqueous NH_4Cl solution (degassed, 10 mL) and dried with $MgSO_4$. After removal of all volatiles, a mixture of $DPPh_2$ and $HPPh_2$ (55/45, [^{31}P]) was obtained as a colorless liquid (2.20 g, 11.8 mmol, 74%).

^{31}P{^{1}H} NMR (243 MHz, 299 K, CD_2Cl_2): δ = -40.2 ($\nu_{1/2}$ ~ 2 Hz, $HPPh_2$), -41.6 (1:1:1 t, $^{1}J_{PD}$ = 33.9 Hz, $DPPh_2$).

^{31}P NMR (243 MHz, 299 K, CD_2Cl_2): δ = -40.2 (dm, $^{1}J_{PH}$ = 219.0 Hz, $HPPh_2$), -41.6 (1:1:1 tm, $^{1}J_{PD}$ = 33.9 Hz, $DPPh_2$).

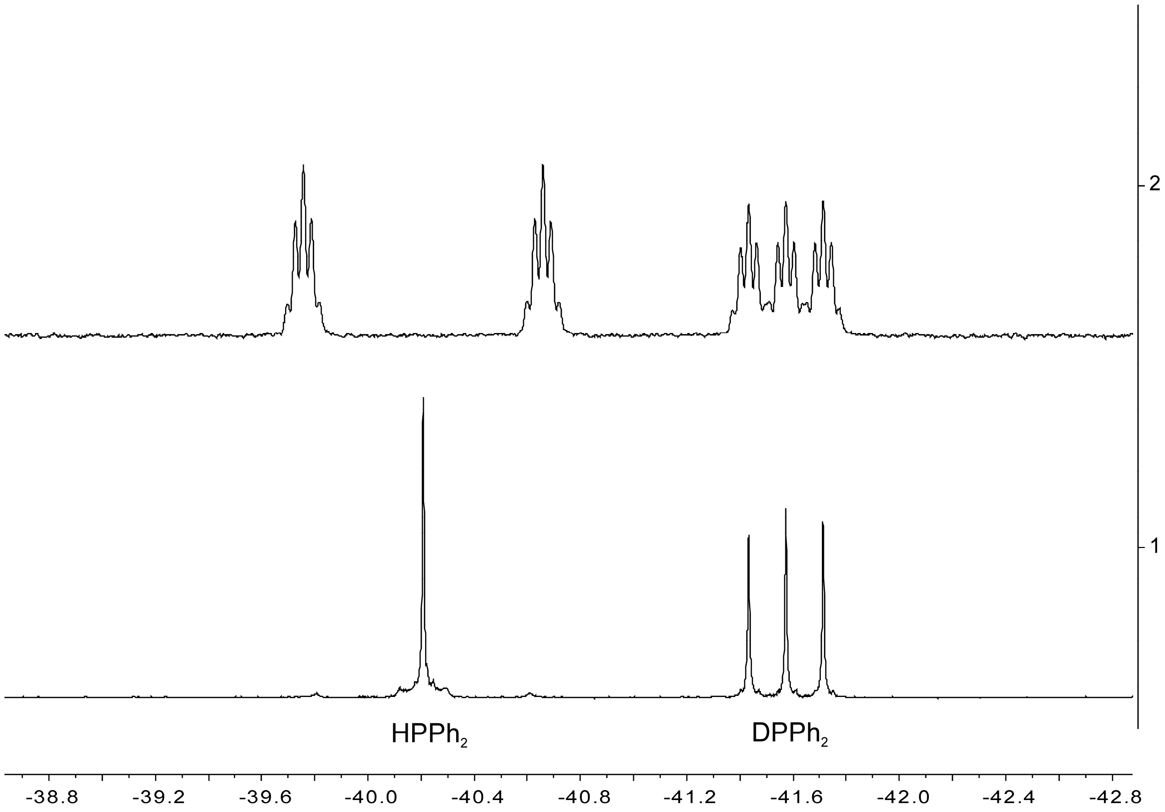

Figure S33: ^{31}**P{^{1}H} NMR** (243 MHz, 299 K, CD_2Cl_2) of $DPPh_2$ and $HPPh_2$ (55/45).

*b) Preparation of compound **17a**-D:* Following the procedure as described for the preparation of compound **17a**: bis(pentafluorophenyl)borane (103.8 mg, 0.3 mmol, 1 eq) and 2-methylbut-1-en-3-yne (21.8 mg, 0.33 mmol, 1.1 eq) in toluene (2 mL) were stirred for 3 h at room temperature. Then deuterodiphenylphosphane (56.1 mg, 0.3 mmol, 1eq, $DPPh_2/HPPh_2$ ~ 55:45) in toluene (1 mL) was added to finally give (80 °C for 3 d) a mixture of **17a**-D and **17a** (ca. 50/50, [^{1}H])(127.1 mg, 0.21 mmol, 71 %) as a white solid.

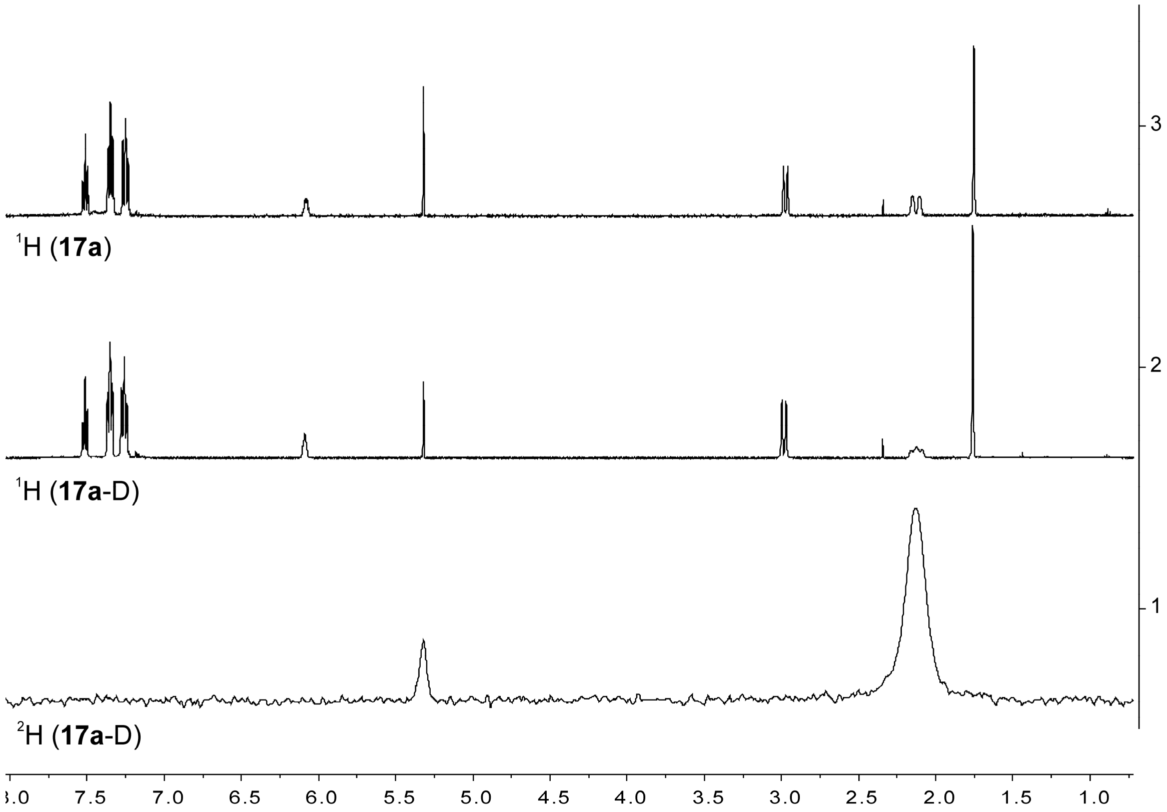

Figure S34: (1) ^{1}**H NMR** (500 MHz, 299K, CD_2Cl_2) of compound **17a**. (2) ^{1}**H NMR** (500 MHz, 299, CD_2Cl_2) of compound **17a**-D. (3) ^{2}**H NMR** (77 MHz, 299K, CH_2Cl_2) of compound **17a**-D.

Synthesis of compound 17b.

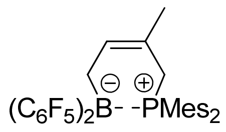

Scheme S10

Bis(pentafluorophenyl)borane (0.346 g, 1.0 mmol, 1 eq) and 2- methylbut-1-en-3-yne (0.072 g, 1.1 mmol, 1.1 eq) were suspended in toluene (5 mL) and stirred for 4 h at room temperature. Then dimesitylphosphane (0.270 g, 1.0 mmol, 1 eq) in toluene (5 mL) was added and the reaction mixture was heated at 60 °C for 16 h. Subsequently all volatiles were removed in vacuo and the residue was extracted with pentane (50 mL) *via* filter cannula. The solvent of the solution was removed in vacuo and the obtained solid was washed with cold pentane (3x1 mL). After drying in vacuo compound **17b** (0.477 g, 0.70 mmol, 70 %) was obtained as a light yellow solid. Crystals suitable for the X-ray crystal structure analysis were obtained by slow diffusion of pentane to a dichloromethane solution of compound **17b** at -35

^{o}C. **IR** (KBr): $\tilde{\nu}$ / cm^{-1} = 3028, 2979, 2925, 1641, 1606, 1516, 1450, 1381, 1281, 1098, 971, 785. **M.p.**: 167 ^{o}C. **Anal. Calc.** for $C_{35}H_{30}BF_{10}P$: C: 61.60; H: 4.43. Found: C: 61.52; H: 4.29.

^{1}H NMR (600 MHz, 243 K, CD_2Cl_2): δ = 7.01 (s, 1H, *m*-Mes^{a}), 6.88 (d, $^{4}J_{PH}$ = 4.0 Hz, 1H, *m*-Mes^{b}), 6.78 (s, 1H, *m*'-Mes^{b}), 6.58 (s, 1H, *m*'-Mes^{a}), 5.46 (br m, 1H, HC=), 3.98, 2.71 (each m, each 1H, PCH_2), 2.69 (s, 3H, *o*-$CH_3^{Mes,a}$), 2.50 (d, $^{4}J_{PH}$ = 1.8 Hz, 3H, *o*-$CH_3^{Mes,b}$), 2.25 (s, 3H, *p*-$CH_3^{Mes,a}$), 2.23 (m), 1.71 (ddd, $^{3}J_{PH}$ = 43.5 Hz, $^{2}J_{HH}$ = 17.2 Hz, $^{3}J_{HH}$ = 9.5 Hz)(each 1H, BCH_2), 2.20 (s, 3H, *p*-$CH_3^{Mes,b}$), 2.07 (s, 3H, *o*'-$CH_3^{Mes,b}$), 1.62 (s, 3H, CH_3), 1.13 (s, 3H, *o*'-$CH_3^{Mes,a}$).

^{13}C{^{1}H} NMR (151 MHz, 243 K, CD_2Cl_2): δ = 142.8 (d, $^{2}J_{PC}$ = 3.7 Hz, *o*-Mes^{a}), 142.6 (dm, $^{2}J_{PC}$ = 15.4 Hz, *o*-Mes^{b}), 141.8 (d, $^{2}J_{PC}$ = 12.3 Hz, *o*'-Mes^{a}), 140.9 (d, $^{4}J_{PC}$ = 2.5 Hz, *p*-Mes^{a}), 140.9 (*o*'-Mes^{b}), 140.5 (d, $^{4}J_{PC}$ = 2.2 Hz, *p*-Mes^{b}), 132.0 (d, $^{3}J_{PC}$ = 8.3 Hz, *m*-Mes^{a}), 131.6 (d, $^{3}J_{PC}$ = 6.9 Hz, *m*'-Mes^{b}), 130.9 (d, $^{3}J_{PC}$ = 9.6 Hz, *m*'-Mes^{a}), 130.5 (d, $^{1}J_{PC}$ = 45.8 Hz, *i*-Mes^{b}), 129.4 (d, $^{3}J_{PC}$ = 9.2 Hz, *m*-Mes^{b}), 128.4 (d, $^{3}J_{PC}$ = 5.7 Hz, HC=), 128.3 (d, $^{2}J_{PC}$ = 3.5 Hz, =CMe), 123.3 (br, C_6F_5), 122.8 (dd, $^{1}J_{PC}$ = 51.2 Hz, J = 2.7 Hz, *i*-Mes^{a}), 31.6 (dd, $^{1}J_{PC}$ = 31.6 Hz, J = 11.1 Hz, PCH_2), 24.8 (d, $^{3}J_{PC}$ = 12.4 Hz, CH_3), 23.9 (m, *o*-$CH_3^{Mes,b}$), 22.8 (d, $^{3}J_{PC}$ = 6.5 Hz, *o*'-$CH_3^{Mes,a}$), 22.4 (d, $^{3}J_{PC}$ = 3.0 Hz, *o*'-$CH_3^{Mes,b}$), 22.1 (d, $^{3}J_{PC}$ = 1.9 Hz, *o*-$CH_3^{Mes,a}$), 20.6 (d, $^{5}J_{PC}$ = 1.0 Hz, *p*-$CH_3^{Mes,a}$), 20.5 (d, $^{5}J_{PC}$ = 1.0 Hz, *p*-$CH_3^{Mes,b}$), 19.5 (br, BCH_2), [C_6F_5 not listed].

^{11}B{^{1}H} NMR (192 MHz, 243 K, CD_2Cl_2): δ = -8.1 ($\nu_{1/2}$ ~ 500 Hz).

^{31}P{^{1}H} NMR (243 MHz, 243 K, CD_2Cl_2): δ = 1.7 ($\nu_{1/2}$ ~ 60 Hz).

^{19}F NMR (564 MHz, 243 K, CD_2Cl_2): δ = -125.5 (d, $^{3}J_{FF}$ = 22.5 Hz, 1F, *o*), -127.4 (d, $^{3}J_{FF}$ = 22.0 Hz, 1F, *o*'), -158.0 (t, $^{3}J_{FF}$ = 20.4 Hz, 1F, *p*), -164.7 (m, 2F, *m*-, *m*')($BC_6F_5^{a}$)[$\Delta\delta^{19}F_{m,p}$ = 6.7], -128.6 (br, *o*), -131.5 (d, $^{3}J_{FF}$ = 24.0 Hz, *o*'), -160.9 (t, $^{3}J_{FF}$ = 21.0 Hz, *p*), -165.2 (m, *m*'), -165.8 (m, *m*)(each 1F, $BC_6F_5^{b}$)[$\Delta\delta^{19}F_{m,p}$ = 4.3, 4.9].

^{1}H, ^{1}H GCOSY (600 MHz / 600 MHz, 243 K, CD_2Cl_2)[selected traces]: δ ^{1}H / δ ^{1}H = 7.01 / 6.58, 2.69, 2.25, 1.13 (*m*-Mes^{a} / *m*'-Mes^{a}, *o*-$CH_3^{Mes,a}$, *p*-$CH_3^{Mes,a}$, *o*'-$CH_3^{Mes,a}$), 6.88 / 6.78, 2.50, 2.20, 2.07 (*m*-Mes^{b} / *m*'-Mes^{b}, *o*-$CH_3^{Mes,b}$, *p*-$CH_3^{Mes,b}$, *o*'-$CH_3^{Mes,b}$), 5.46 / 3.98, 2.71, 2.24, 1.71 (HC= / PCH_2, PCH_2, BCH_2, BCH_2), 3.98 / 2.71, 2.24, 1.62 (PCH_2 / PCH_2, BCH_2, CH_3).

^{1}H, ^{13}C GHSQC (600 MHz / 151 MHz, 243 K, CD_2Cl_2): δ ^{1}H / δ ^{13}C = 7.01 / 132.0 (*m*-Mes^{a}), 6.88 / 129.4 (*m*-Mes^{b}), 6.78 / 131.6 (*m*'-Mes^{b}), 6.58 / 130.9 (*m*'-Mes^{a}), 5.46 / 128.4 (HC=), 3.98, 2.71 / 31.6 (PCH_2), 2.69 / 22.1 (*o*-$CH_3^{Mes,a}$), 2.50 / 23.9 (*o*-$CH_3^{Mes,b}$), 2.25 / 20.6 (*p*-$CH_3^{Mes,a}$), 2.24, 1.71 / 19.5 (BCH_2), 2.20 / 20.5 (*p*-$CH_3^{Mes,b}$), 2.07 / 22.4 (*o*'-$CH_3^{Mes,b}$), 1.62 / 24.8 (CH_3), 1.13/ 22.8 (*o*'-$CH_3^{Mes,a}$).

^{1}H, ^{13}C GHMBC (600 MHz / 151 MHz, 243 K, CD_2Cl_2)[selected traces]: δ ^{1}H / δ ^{13}C = 5.46 / 31.6, 24.8 (HC= / PCH_2, CH_3), 2.69 / 142.8, 132.0, 122.8 (*o*-$CH_3^{Mes,a}$ / *o*-Mes^{a}, *m*-Mes^{a}, *i*-Mes^{a}), 2.50 / 142.6, 130.5, 129.4 (*o*-$CH_3^{Mes,b}$ / *o*-Mes^{b}, *i*-Mes^{b}, *m*-Mes^{b}), 2.25 / 140.9, 132.0, 130.9 (*p*-$CH_3^{Mes,a}$ / *p*-Mes^{a}, *m*-Mes^{a}, *m*'-Mes^{a}), 2.20 / 140.5, 131.6, 129.4 (*p*-$CH_3^{Mes,b}$ / *p*-Mes^{b}, *m*'-Mes^{b}, *m*-Mes^{b}), 2.07 / 140.8, 131.6, 130.5 (*o*'-$CH_3^{Mes,b}$ / *o*'-Mes^{b}, *m*'-Mes^{b}, *i*-Mes^{b}), 1.13/ 141.8, 130.9, 122.8 (*o*'-$CH_3^{Mes,a}$ / *o*'-Mes^{a}, *m*'-Mes^{a}, *i*-Mes^{a}).

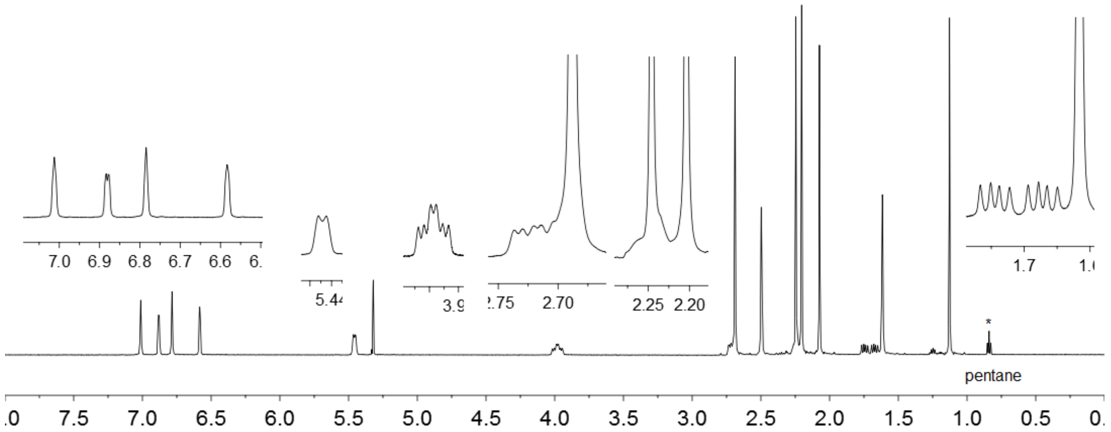

Figure S35: **^{1}H NMR** (600 MHz, 243 K, CD_2Cl_2) of compound **17b**

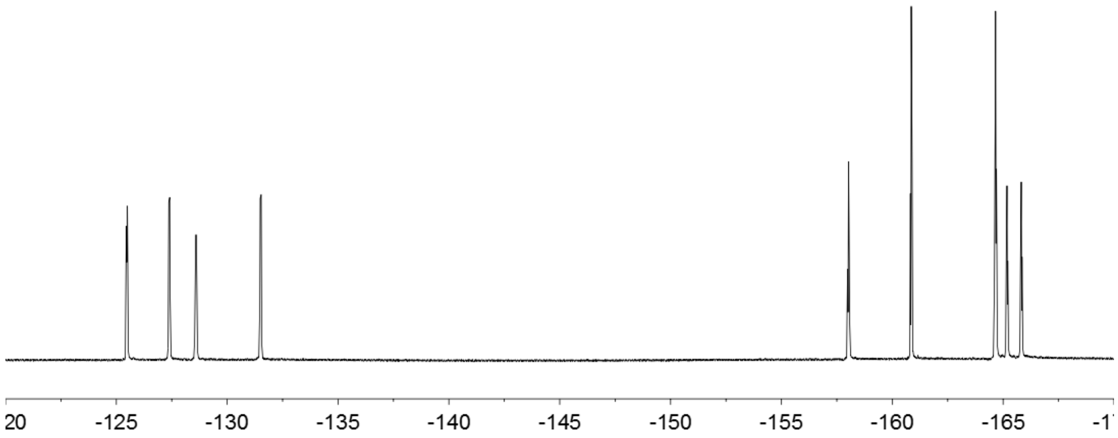

Figure S36: **^{19}F{^{1}H} NMR** (564 MHz, 243 K, CD_2Cl_2) of compound **17b**

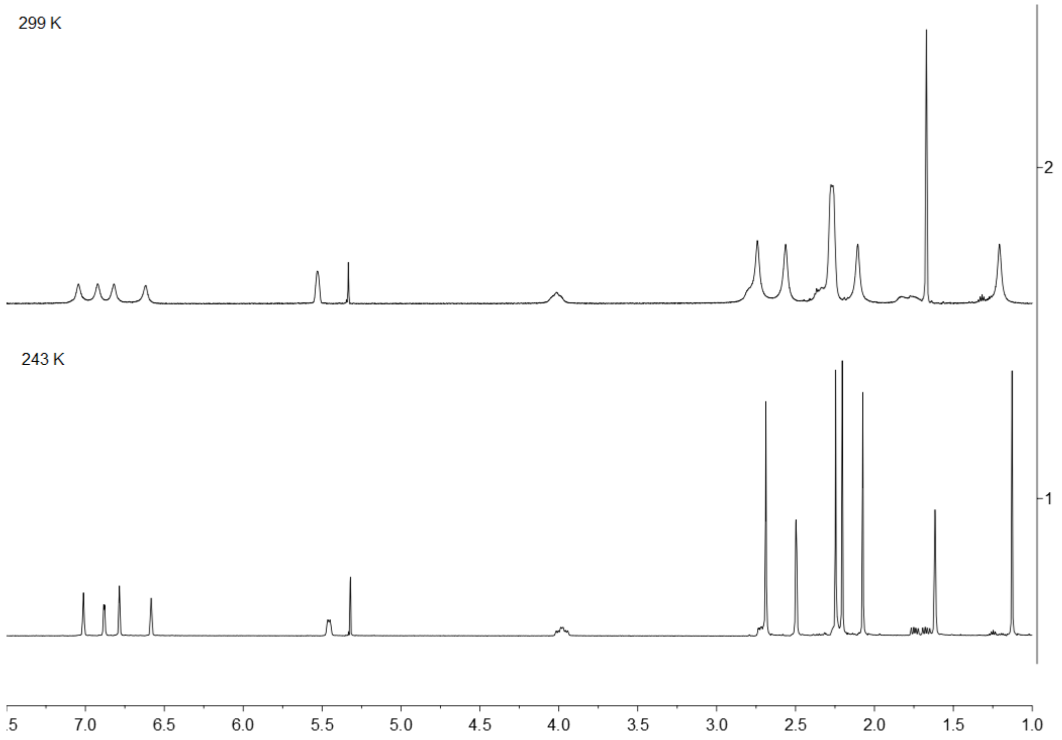

Figure S37: **^{1}H NMR** (600 MHz, CD_2Cl_2) of compound **17b** at 299K (2) and 243K (1).

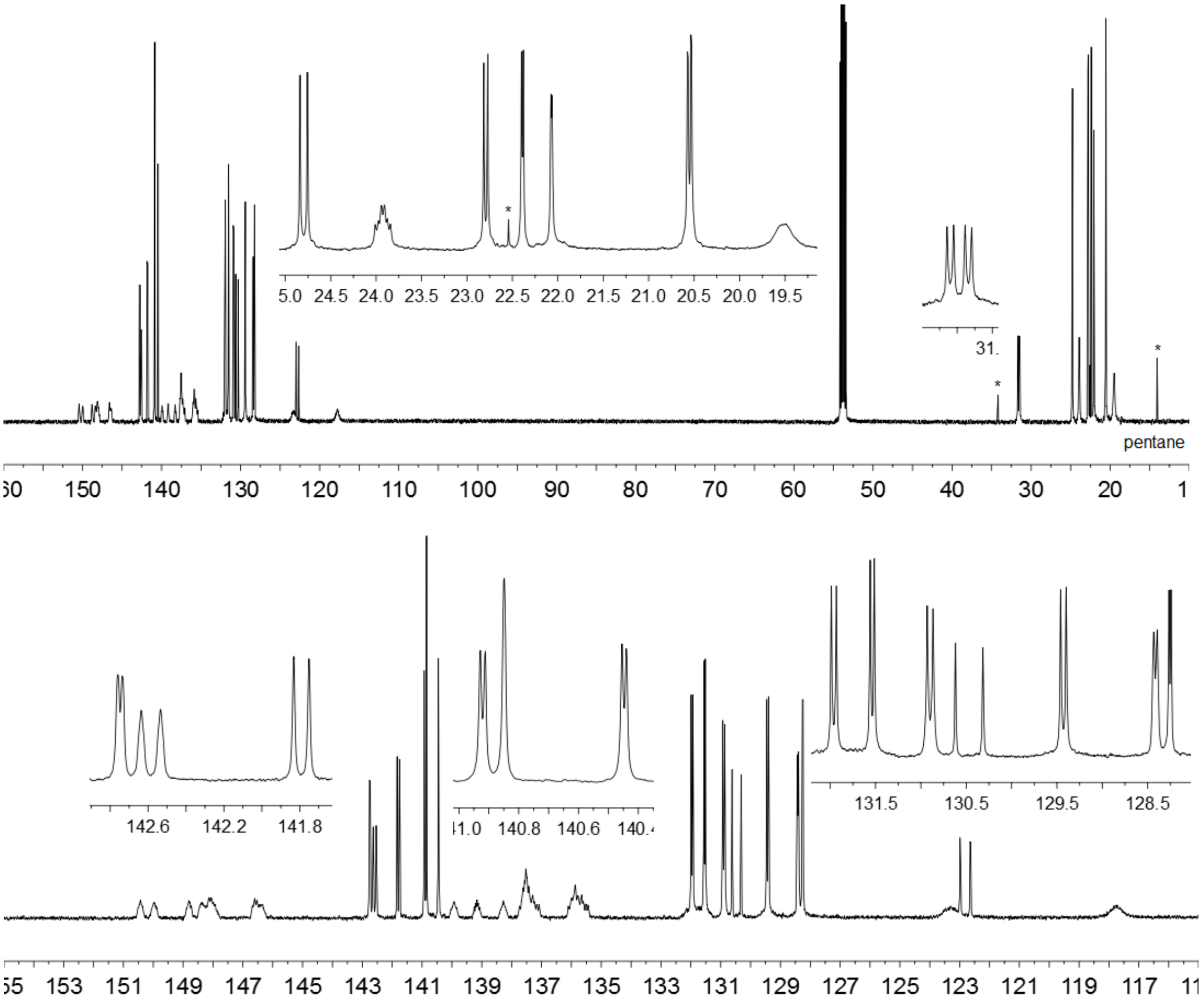

Figure S38: **^{13}C{^{1}H} NMR** (151 MHz, 243 K, CD_2Cl_2) of compound **17b**

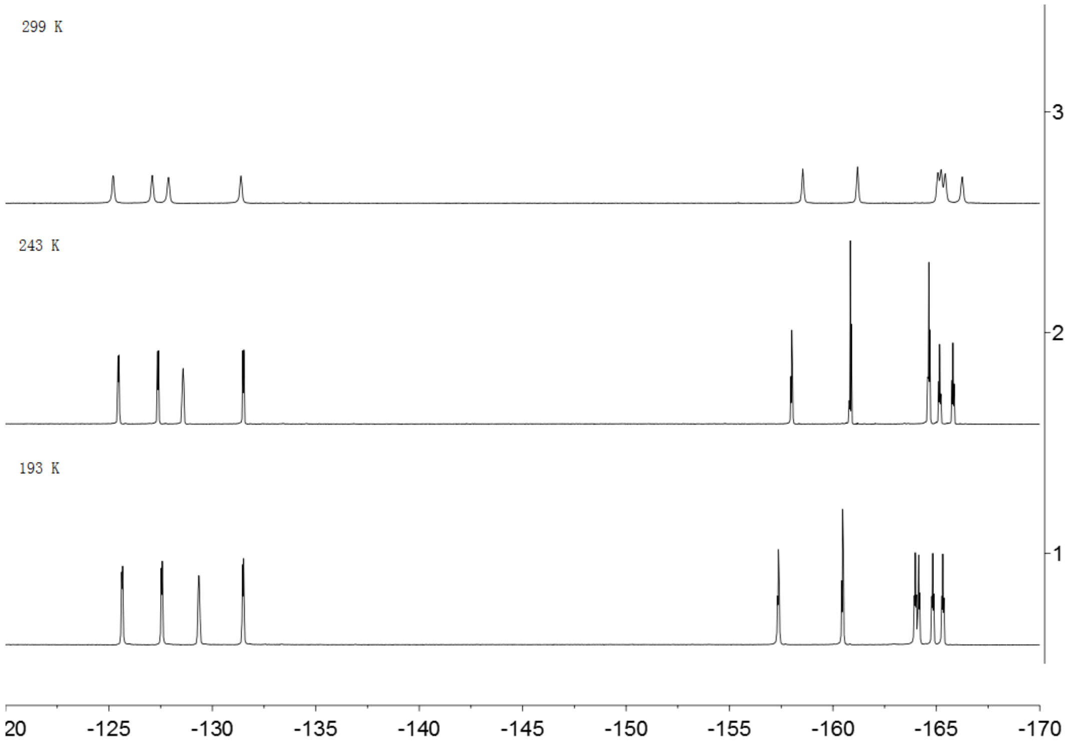

Figure S39: **^{19}F NMR** (470 MHz, CD_2Cl_2) of compound **17b**

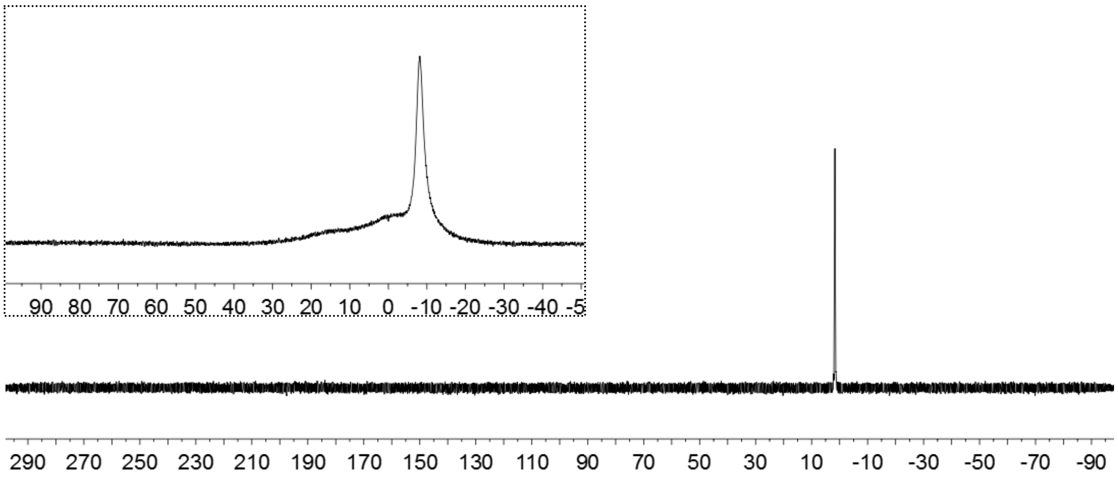

Figure S40: **^{11}B{^{1}H} NMR** (192 MHz, 243 K, CD_2Cl_2) and **^{31}P{^{1}H} NMR** (243 MHz, 243 K, CD_2Cl_2) of compound **17b**

Figure S41: **X-ray crystal structure analysis of compound 17b**. formula $C_{29}H_{18}BF_{10}P$, M = 682.37, colourless crystal, 0.33 x 0.23 x 0.17 mm, a = 9.1195(3), b = 19.9550(5), c = 18.0470(5) Å, β = 91.390(2) °, V = 3283.2(2) Å^3, ρ_{calc} = 1.380 gcm^{-3}, μ = 1.470 mm^{-1}, empirical absorption correction (0.642 ≤ T ≤ 0.788), Z = 4, monoclinic, space group $P2_1/c$ (No. 14), λ = 1.54178 Å, T = 223(2) K, ω and φ scans, 22341 reflections collected (±h, ±k, ±l), [(sinθ)/λ] = 0.60 Å^{-1}, 5728 independent (R_{int} = 0.043) and 5215 observed reflections [I>2σ(I)], 431 refined parameters, R = 0.043, wR^2 = 0.119, max. (min.) residual electron density 0.25 (-0.23) e.Å^{-3}, hydrogen atoms calculated and refined as riding atoms.

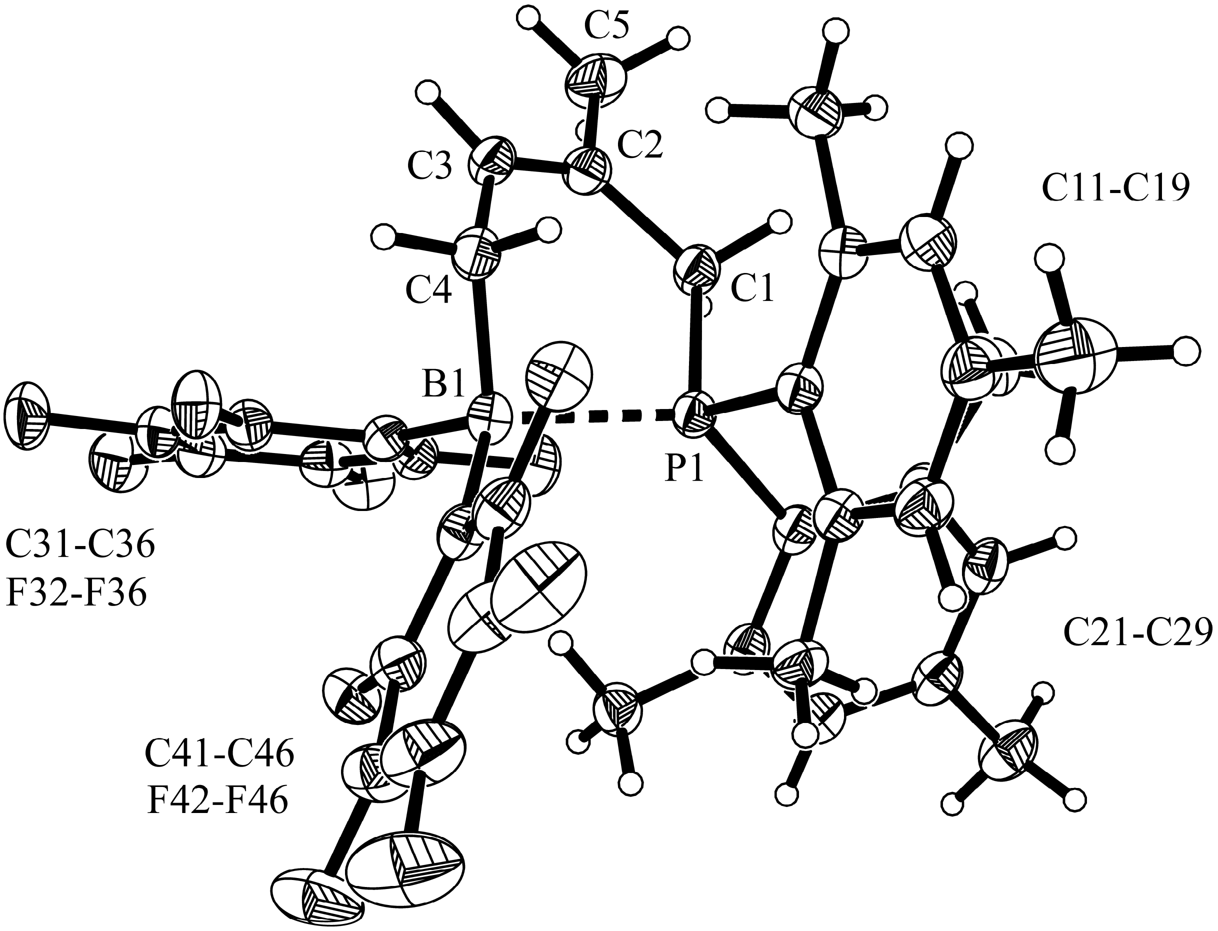

Synthesis of compound 17c.

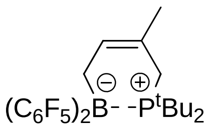

Scheme S11

Bis(pentafluorophenyl)borane (0.346 g, 1.0 mmol, 1 eq) and 2- methylbut-1-en-3-yne (0.072 g, 1.1 mmol, 1.1 eq) were suspended in toluene (5 mL) and stirred for 4 h at room temperature. Then di-*tert*-butylphosphane (0.146 g, 1.0 mmol, 1 eq) in toluene (5 mL) was added and the reaction mixture was heated at 60 °C for 16 h. Subsequently all volatiles were removed in vacuo and the residue was extracted with pentane (50 mL) *via* filter cannula. The solvent of the solution was removed in vacuo and the obtained solid was washed with cold pentane (3x1 mL). After drying in vacuo compound **17c** (0.439 g, 0.79 mmol, 79 %) was obtained as a white solid. Crystals suitable for the X-ray crystal structure analysis were obtained by slow diffusion of pentane to a dichloromethane solution of compound **17c** at -35

°C. **IR** (KBr): $\tilde{\upsilon}$ / cm^{-1} = 2974, 2912, 1643, 1517, 1456, 1373, 1279, 1098, 975, 778. **M.p.**: 187 °C. **Anal. Calc.** for $C_{25}H_{26}BF_{10}P$: C: 53.79; H: 4.69. Found: C: 54.13; H: 4.53.

^{1}H NMR (500 MHz, 299 K, CD_2Cl_2): δ = 5.78 (br, 1H, HC=), 2.38 (d, $^2J_{PH}$ = 10.7 Hz, 2H, PCH_2), 2.03 (br, 2H, BCH_2), 1.89 (s, 3H, CH_3), 1.27 (d, $^3J_{PH}$ = 12.7 Hz, 18H, ^{t}Bu).

^{13}C{^{1}H} NMR (126 MHz, 299 K, CD_2Cl_2): δ = 148.2 (dm, $^1J_{FC}$ ~ 240 Hz, C_6F_5), 139.6 (dm, $^1J_{FC}$ ~ 250 Hz, C_6F_5), 137.5 (dm, $^1J_{FC}$ ~ 250 Hz, C_6F_5), 131.1 (d, $^3J_{PC}$ = 9.9 Hz, HC=), 124.7 (d, $^2J_{PC}$ = 5.5 Hz, =CMe), 124.2 (br, *i*-C_6F_5), 37.3 (d, $^1J_{PC}$ = 17.5 Hz, ^{t}Bu), 29.8 (^{t}Bu), 27.3 (br, BCH_2), 26.0 (d, $^3J_{PC}$ = 6.5 Hz, CH_3), 21.5 (d, $^1J_{PC}$ = 30.4 Hz, PCH_2).

^{11}B{^{1}H} NMR (160 MHz, 299 K, CD_2Cl_2): δ = -13.9 (br d, $^1J_{PB}$ ~ 55 Hz).

^{31}P{^{1}H} NMR (202 MHz, 299 K, CD_2Cl_2): δ = 15.0 (1:1:1:1 q, partial relaxed $^1J_{PB}$ ~ 55 Hz).

^{19}F NMR (470 MHz, 299 K, CD_2Cl_2): δ = -127.8 (br, 2F, *o*-BC_6F_5), -159.7 (br, 1F, *p*-BC_6F_5), -164.8 (br, 2F, *m*-BC_6F_5), [$\Delta\delta^{19}F_{m,p}$ = 5.1].

^{19}F NMR (470 MHz, 193 K, CD_2Cl_2): δ = -124.0 (m, *o*), -129.8 (m, *o*'), -160.7 (t, $^3J_{FF}$ = 21.3 Hz, *p*), -164.0 (m, *m*'), -165.0 (m, *m*)(each 1F, $BC_6F_5^a$)[$\Delta\delta^{19}F_{m,p}$ = 3.3, 4.3], -125.5 (m, *o*), -132.1 (m, *o*'), -157.4 (br t, $^3J_{FF}$ = 21.1 Hz, *p*), -163.0 (m, *m*'), -163.8 (m, *m*)(each 1F, $BC_6F_5^b$)[$\Delta\delta^{19}F_{m,p}$ = 5.6, 6.4].

^{1}H, ^{1}H GCOSY (500 MHz / 500 MHz, 299 K, CD_2Cl_2)[selected trace]: δ ^{1}H / δ ^{1}H = 5.78 / 2.38, 2.03, 1.89 (HC= / PCH_2, BCH_2, CH_3).

^{1}H, ^{13}C GHSQC (500 MHz / 126 MHz, 299 K, CD_2Cl_2): δ ^{1}H / δ ^{13}C = 5.78 / 131.1 (HC=), 2.38 / 21.5 (PCH_2), 1.89 / 26.0 (CH_3), 1.27 / 29.8 (^{t}Bu).

^{1}H, ^{13}C GHMBC (500 MHz / 126 MHz, 299 K, CD_2Cl_2)[selected traces]: δ ^{1}H / δ ^{13}C = 1.89 / 131.1, 124.6, 21.5 (CH_3 / HC=, =CMe, PCH_2), 1.27 / 37.3, 29.8 (^{t}Bu / ^{t}Bu, ^{t}Bu).

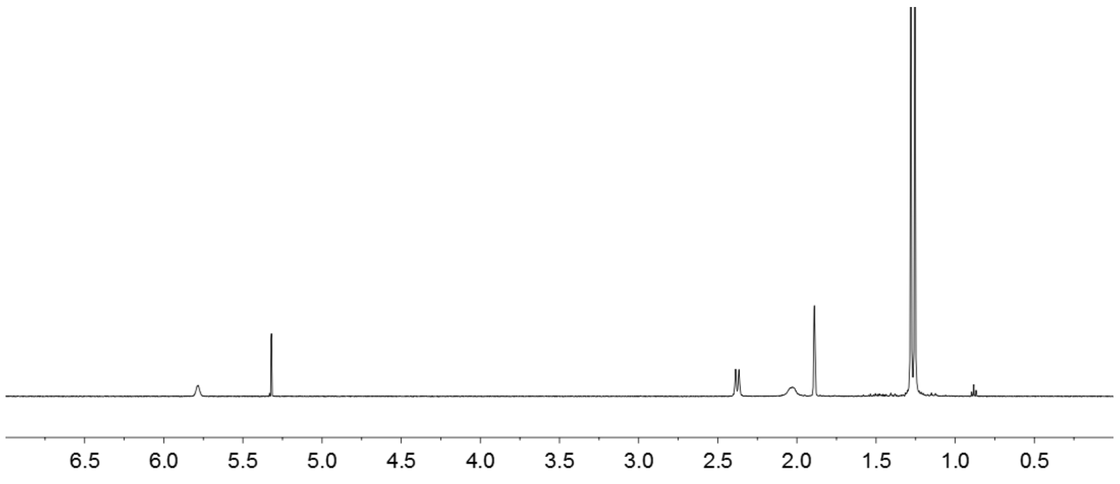

Figure S42: **^{1}H NMR** (500 MHz, 299 K, CD_2Cl_2) of compound **17c**

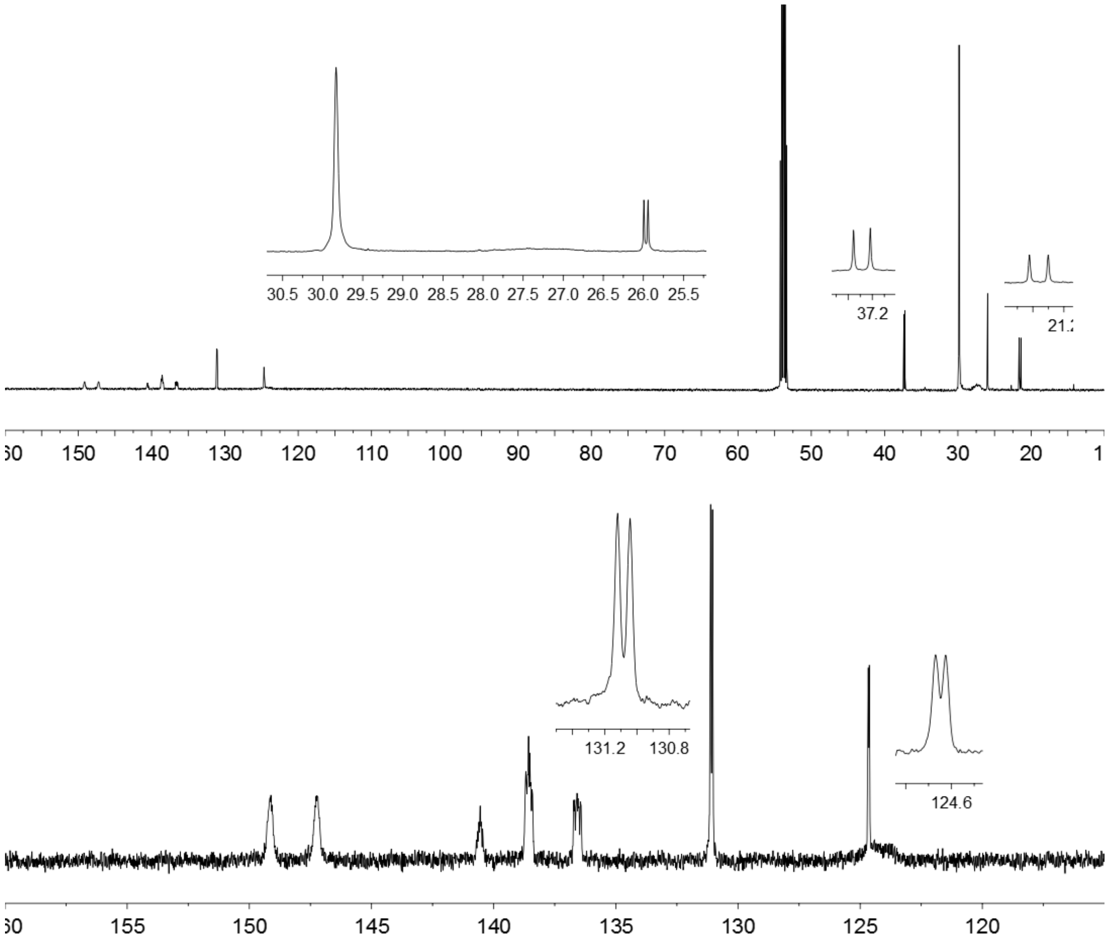

Figure S43: ^{13}**C{^{1}H} NMR** (126 MHz, 299 K, CD_2Cl_2) of compound **17c**

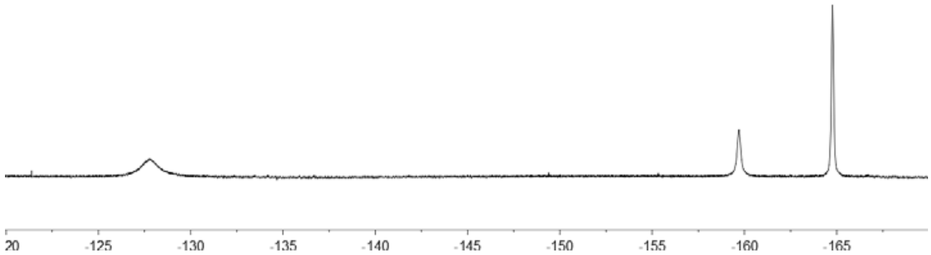

Figure S44: ^{19}**F NMR** (470 MHz, 299 K, CD_2Cl_2) of compound **17c**

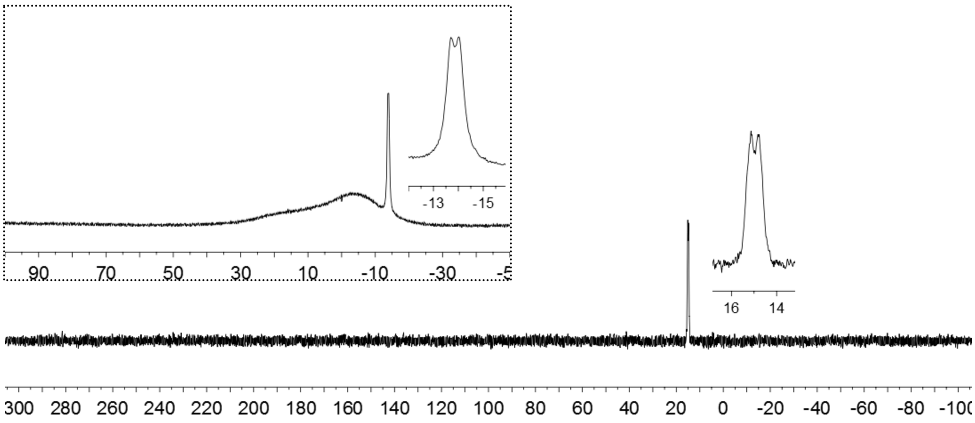

Figure S45: ^{11}**B{^{1}H} NMR** (160 MHz, 299 K, CD_2Cl_2) and ^{31}**P{^{1}H} NMR** (202 MHz, 299 K, CD_2Cl_2) of compound **17c**

Dynamic ^{19}F NMR:

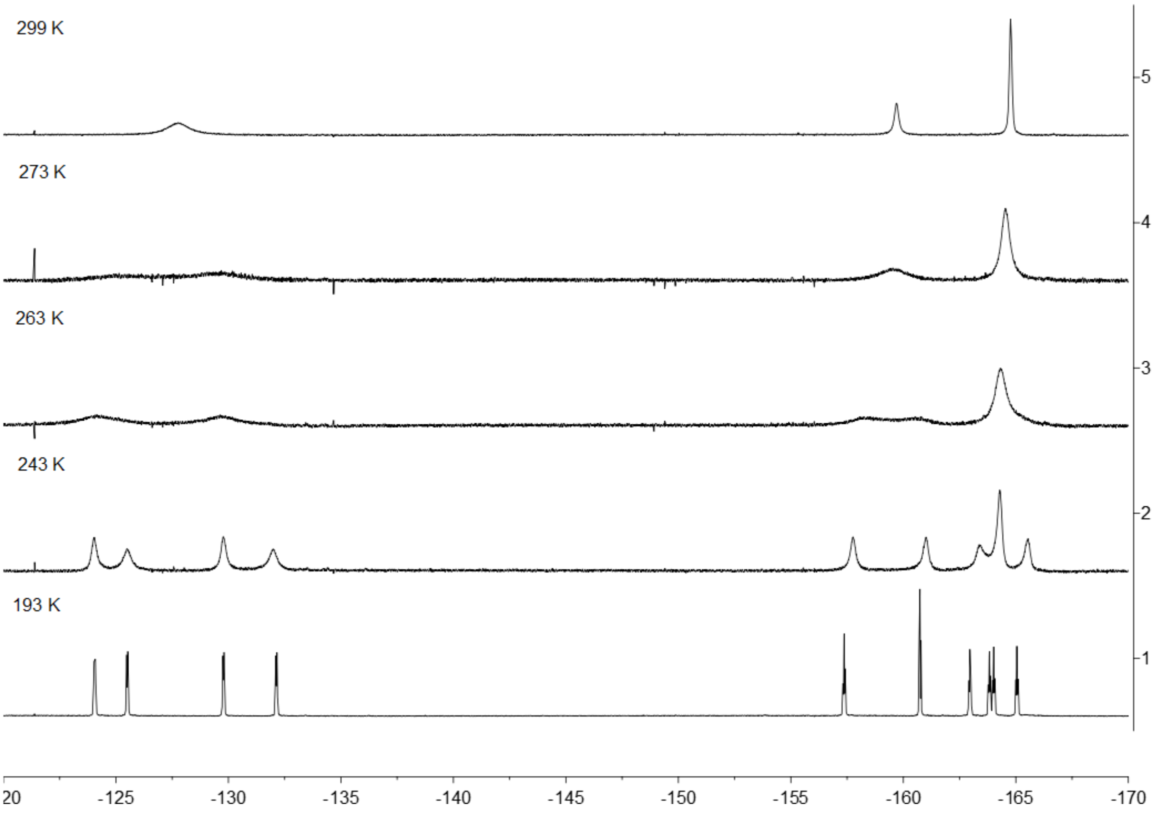

Figure S46: **^{19}F NMR** (470 MHz, CD_2Cl_2) of compound **17c**

$\Delta G^‡ = RT_c(22.96 + \ln(T_c/\delta\nu))$

T_c = coalescence temperature [K]: 268 K (^{19}F, *p*-BC_6F_5)

$\Delta\nu$ = chemical shift difference [Hz] (^{19}F, *p*-BC_6F_5, 193 K): 1586 Hz

R = 8.314 J/(mol·K); 1 J = 0.239 cal

$\Delta G^‡$[268K, δν(193 K) = 1586 Hz] = 47197 J/mol = 11.3 ± 0.3 kcal/mol

Figure S47: **X-ray crystal structure analysis of compound 17c**. formula $C_{25}H_{26}BF_{10}P$, M = 558.24, colourless crystal, 0.15 x 0.12 x 0.03 mm, a = 25.4740(7), b = 12.5753(3), c = 19.1732(4) Å, β = 109.881(2) °, V = 5775.9(2) Å^3, ρ_{calc} = 1.284 gcm^{-3}, μ = 1.547 mm^{-1}, empirical absorption correction (0.801 ≤ T ≤ 0.955), Z = 8, monoclinic, space group $C2/c$ (No. 15), λ = 1.54178 Å, T = 223(2) K, ω and φ scans, 41845 reflections collected (±h, ±k, ±l), [(sinθ)/λ] = 0.60 Å^{-1}, 5118 independent (R_{int} = 0.059) and 4115 observed reflections [I>2σ(I)], 341 refined parameters, R = 0.039, wR^2 = 0.106, max. (min.) residual electron density 0.19 (-0.26) e.Å^{-3}, hydrogen atoms calculated and refined as riding atoms.

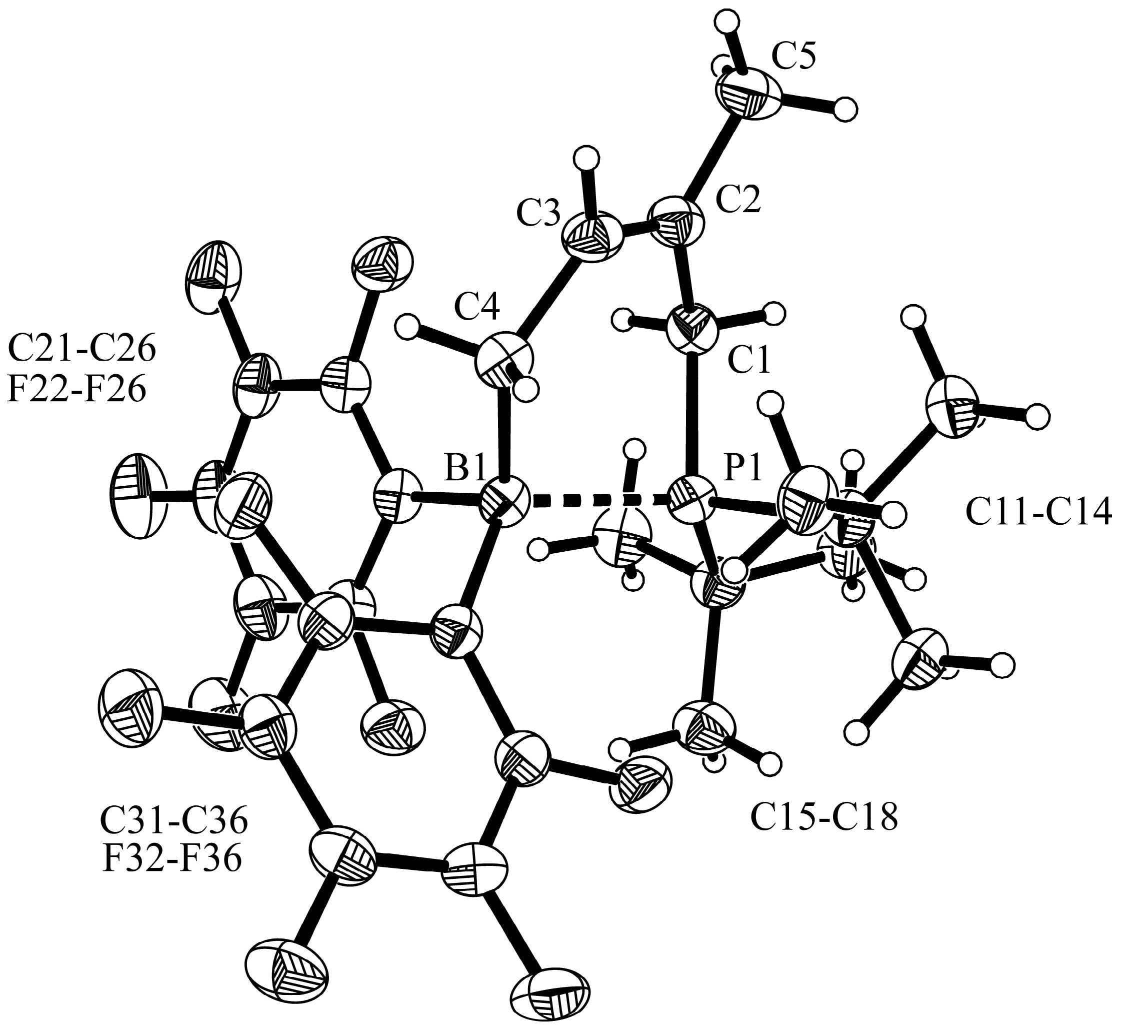
C5
C3
C2
C4
C21-C26
F22-F26
C1
B1
P1
C11-C14
C31-C36
F32-F36
C15-C18

pK_a Calculation

$$R_3P + RCH_2[B] \overset{K}{\rightleftharpoons} R_3\overset{\oplus}{P}H + R\overset{\ominus}{C}H[B]$$

$$K = \frac{[R_3PH^+]\,[RCH^-B]}{[R_3P]\,[RCH_2B]} = \frac{[R_3PH^+]\,[RCH^-B]\,[H_3O^+]}{[R_3P]\,[H_3O^+]\,[RCH_2B]} = \frac{K_{a(C)}}{K_{a(P)}}$$

$$\log K = \log K_{a(C)} - \log K_{a(P)}$$

$$- \log K = - \log K_{a(C)} - (- \log K_{a(P)}) = pK_{a(C)} - pK_{a(P)}$$

$$\Delta G\ (300\ K) = - RT \ln K = 0.6 \cdot 2.303 \cdot (pK_{a(C)} - pK_{a(P)})$$

DFT calculations

All calculations were performed with the Turbomole programs: a) TURBOMOLE V6.5 2013, a development of University of Karlsruhe and Forschungszentrum Karlsruhe GmbH, 1989-2007, TURBOMOLE GmbH, since 2007; available from http://www.turbomole.com. TPSS functional: a) J. Tao, J. P. Perdew, V. N. Staroverov and G. E. Scuseria, *Phys. Rev. Lett.*, **91**, 146401 (2003); Dispersion correction (D3): b) S. Grimme, J. Antony, S. Ehrlich, H. Krieg, *J. Chem. Phys.* **2010**, *132*, 154104-154123; c) S. Grimme, S. Ehrlich, L. Goerigk, *J. Comput. Chem.* **2011**, *32*, 1456-1465; d) def2-TZVP basis set: F. Weigend; R. Ahlrichs. *Phys. Chem. Chem. Phys.* **2005**, *7*, 3297-3305; e) COSMO solvation model: A. Klamt, G. Schüürmann, *J. Chem. Soc. Perkin Trans.* **1993**, *2*, 799-805. The thermodynamic correction was made with the aoforce program using a rotor approximation for low lying modes: f) S. Grimme, Chem. Eur. J. **2012**, *18*, 9955-9964.

All structures were optimized in vacuum calculations, the harmonic vibrational frequencies of the normal modes were also obtained without solvent model. COSMO energies were obtained in single point calculations using the gas phase geometries.

Table S1 Energies and thermodynamic corrections for CH-boryl and other reference compounds, and the respective deprotonated bases (cf. Table 1 of the manuscript) obtained with TPSS-D3/def2-TZVP (+ COSMO corrections)

Entry	Compound	E_{vac} [E_h]	E(ε=9.08) [E_h]	E(ε=46.7) [E_h]	E(ε=78.3) [E_h]	G^{298} [kcal mol^{-1}]
1	C_5H_9-H	-196.67335309	-196.67412277	-196.67424208	-196.67425435	69.028
1	C_5H_9[illegible]	-195.99469796	-196.07547556	-196.08833669	-196.08966920	58.444
2	$PhCH_2$-H	-271.72882883	-271.73255238	-271.73318668	-271.73325287	59.943
2	$PhCH_2$[illegible]	-271.10273802	-271.17740361	-271.18887840	-271.19005802	49.990
3	9-BBN-CH_2-H	-378.28827058	-378.28953895	-378.28973686	-378.28975724	130.516
3	9-BBN-CH_2[illegible]	-377.68842831	-377.75650027	-377.76720656	-377.76831258	122.545
4	mes_2B-CH_2-H	-764.44412508	-764.44412508	-764.44511022	-764.44521305	208.441
4	mes_2B-CH_2[illegible]	-763.85971493	-763.92037675	-763.93028479	-763.93131575	199.776
5	$(C_6F_5)_2B$-CH_2-H	-1521.23893679	-1521.24428409	-1521.24517225	-1521.24526463	56.086
5	$(C_6F_5)_2B$-CH_2[illegible]	-1520.69282381	-1520.74606815	-1520.75432854	-1520.75517956	47.919
6	C_5H_5-H	-194.21976736	-194.22309545	-194.22365421	-194.22371242	40.377
6	C_5H_5[illegible]	-193.63736335	-193.71985115	-193.73237738	-193.73366171	31.464
7	$(C_6F_5)_2B$-$C(CH_3)_2$-H	-1599.90673422	-1599.91212501	-1599.91301684	-1599.91310952	89.515
7	$(C_6F_5)_2B$-$C(CH_3)_2$[illegible]	-1599.37031650	-1599.42044798	-1599.42811315	-1599.42890066	81.055
8	$(C_6F_5)_2B$-CH(Ph)-H	-1752.42967892	-1752.43667516	-1752.43784159	-1752.43796299	103.068
8	$(C_6F_5)_2B$-CH(Ph)[illegible]	-1751.90200392	-1751.95324366	-1751.96127412	-1751.96210318	94.734
9	$(C_6F_5)_2B$-CH(CH=CH_2)-H	-1598.67727462	-1598.68329874	-1598.68429039	-1598.68439336	75.881
9	$(C_6F_5)_2B$-CH(CH=CH_2)[illegible]	-1598.15288260	-1598.20470112	-1598.21273107	-1598.21355829	67.090
10	mes_2EtP-H[illegible]	-1120.69568514	-1120.75053872	-1120.75890974	-1120.75976966	231.387
10	mes_2EtP	-1120.28829630	-1120.29498675	-1120.29616400	-1120.29628782	225.413

Table S2 Relative free enthalpies of deprotonation and derived pK_a values in DMSO and H_2O with $C_5H_5^-$ of CH-boryl and other reference compounds, based on the values in Table S1 (TPSS-D3/def2-TZVP + COSMO)

Entry	"acid"	ΔG^{298} (DMSO) [kcal/mol]	ΔG^{298} (H_2O) [kcal/mol]	pK_a[a] (DMSO)	pK_a[a] (H_2O)
1	C_5H_9-H	+57.68	+57.62	60.4	60.3
2	$PhCH_2$-H	+32.21	+32.28	41.7	41.7
3	9-BBN-CH_2-H	+20.53	+20.61	33.1	33.1
4	mes_2B-CH_2-H	+15.21	+15.40	29.2	29.3
5	$(C_6F_5)_2B$-CH_2-H	+0.45	+0.74	18.3	18.5
6	C_5H_5-H	0.00	0.00	*18.0*[b]	*18.0*[b]
7	$(C_6F_5)_2B$-$C(CH_3)_2$-H	-3.57	-3.24	15.4	15.6
8	$(C_6F_5)_2B$-CH(Ph)-H	-8.68	-8.35	11.6	11.9
9	$(C_6F_5)_2B$-CH(CH=CH_2)-H	-12.28	-11.96	9.0	9.2
10	mes_2EtP-H[illegible]	-15.89	-15.11	6.3	7.2

[a] predicted pK_a calculated at 298 K, obtained from ΔG^{298} and the experimental pK_a value of C_5H_5-H in DMSO (18.0): $pK_a = 18.0 + \Delta G^{298}/(\ln(10)RT)$
[b] experimental value in DMSO (see F. G. Bordwell, G. E. Drucker and H. E. Fried, *J. Org. Chem.* 1981, **46**, 632.)

Figure S48 Optimized molecular conformations (TPSS-D3/def2-TZVP) of CH-boryl and other reference compounds, and the respective deprotonated bases (cf. Table 1 of the manuscript). C-C/C-B distances in Å.

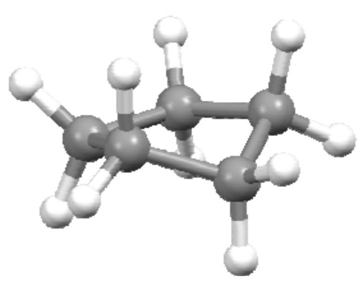

C_5H_9-H

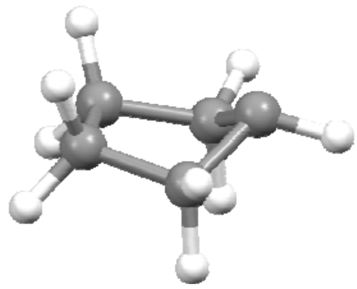

$C_5H_9^-$

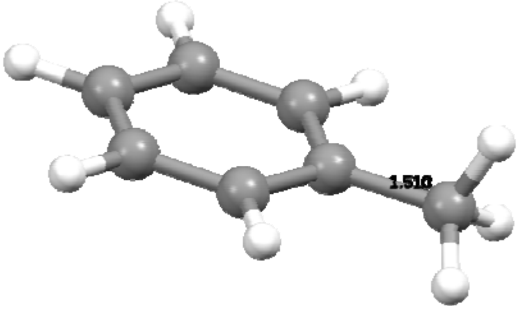

$PhCH_2$-H

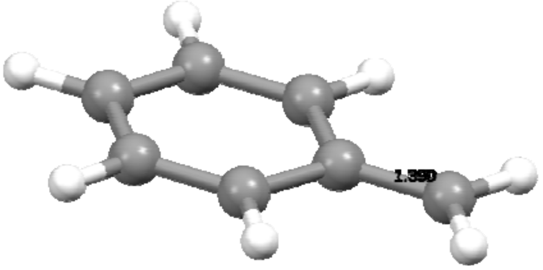

$PhCH_2^-$

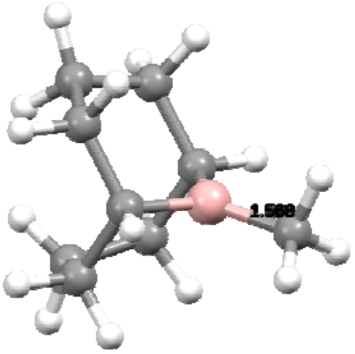

9-BBN-CH_2-H

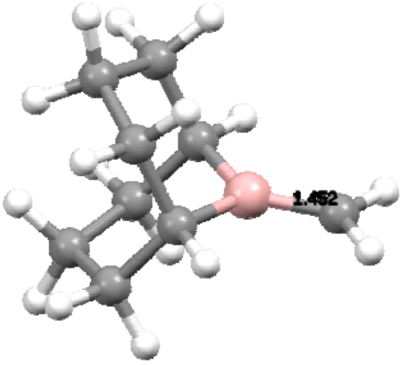

9-BBN-CH_2^-

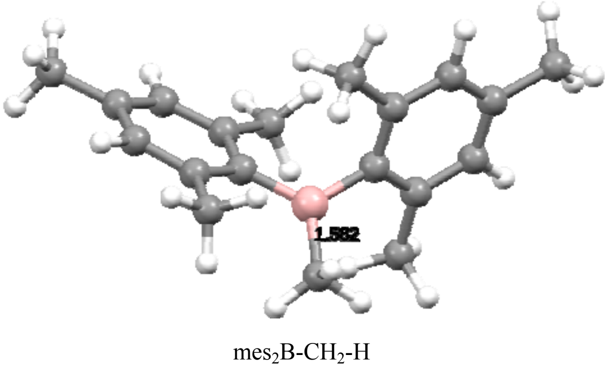

mes_2B-CH_2-H

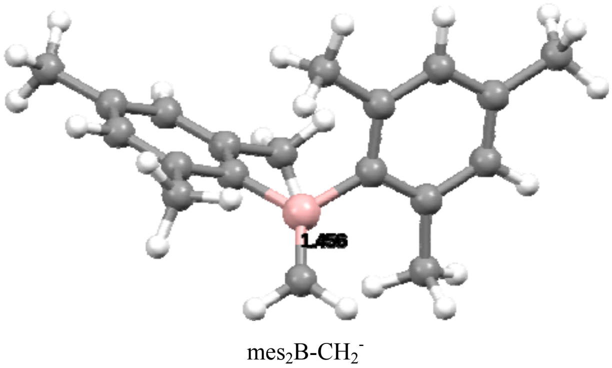

mes_2B-CH_2^-

Figure S48 (continued)

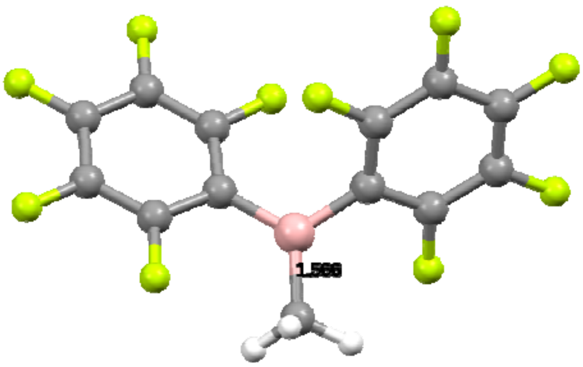

$(C_6F_5)_2B\text{-}CH_2\text{-}H$

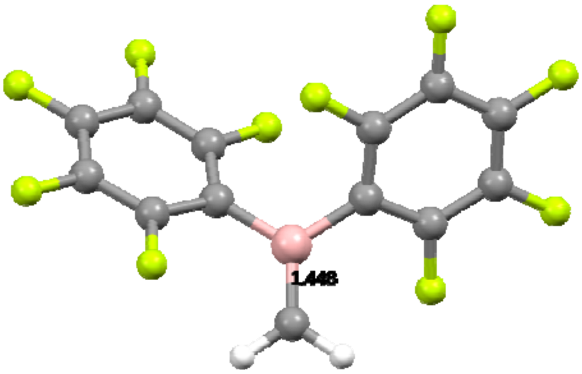

$(C_6F_5)_2B\text{-}CH_2^-$

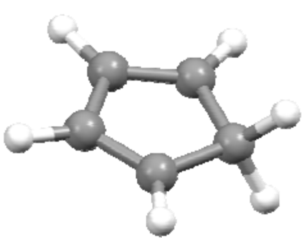

$C_5H_5\text{-}H$

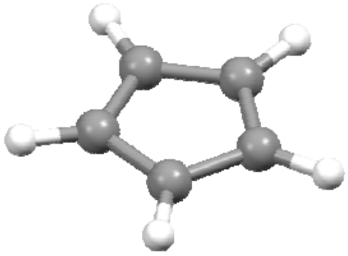

$C_5H_5^-$

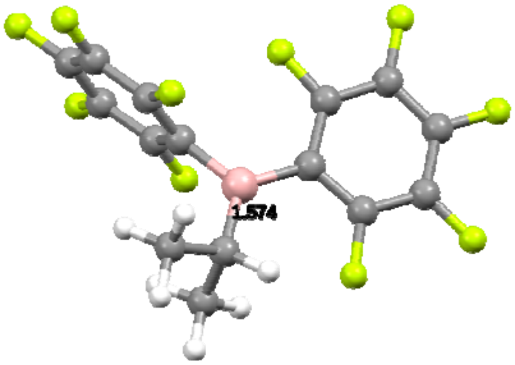

$(C_6F_5)_2B\text{-}C(CH_3)_2\text{-}H$

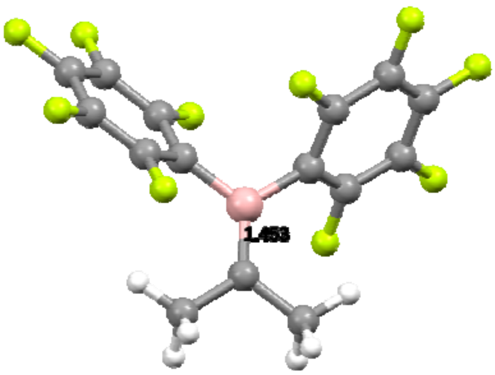

$(C_6F_5)_2B\text{-}C(CH_3)_2^-$

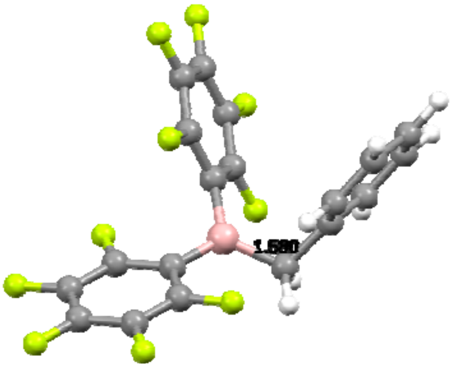

$(C_6F_5)_2B\text{-}CH(Ph)\text{-}H$

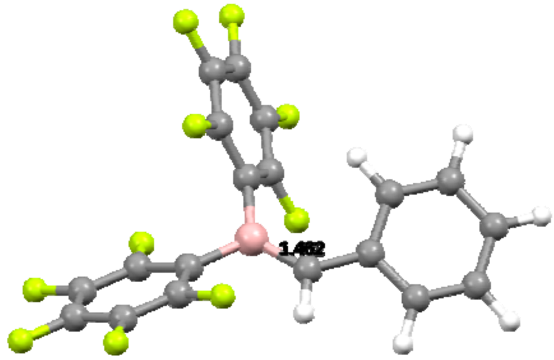

$(C_6F_5)_2B\text{-}CH(Ph)^-$

Figure S48 (continued)

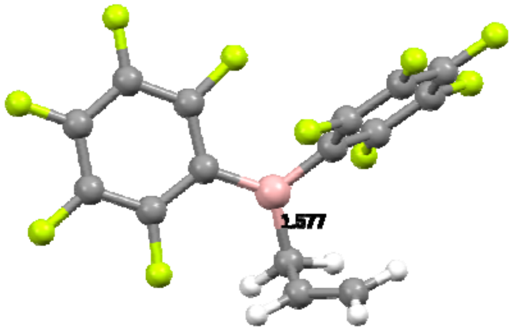

$(C_6F_5)_2B\text{-}CH(CH{=}CH_2)\text{-}H$

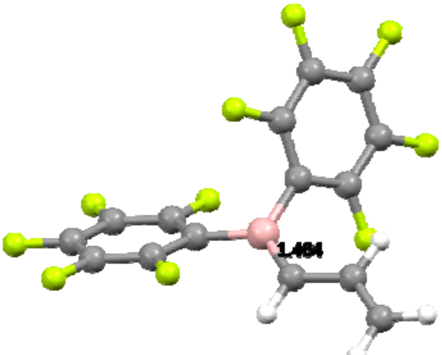

$(C_6F_5)_2B\text{-}CH(CH{=}CH_2)^-$

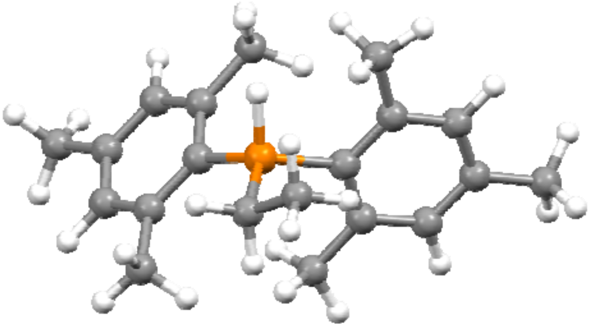

$mes_2EtP\text{-}H^+$

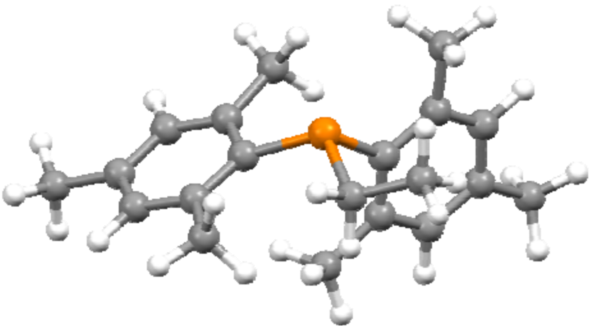

mes_2EtP

Figure S49 HOMO (Highest Occupied Molecular Orbital) of the anions of entries 2, 5, 3, and 7 of Table S2. (TPSS-D3/def2-TZVP)

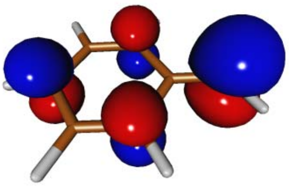

$[Ph\text{-}CH_2]^-$

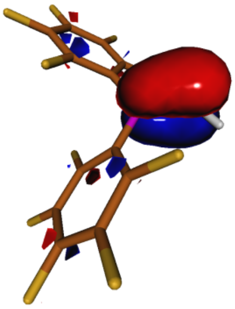

$[(C_6F_5)_2B\text{-}CH_2]^-$

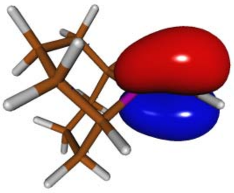

$[9\text{-}BBN\text{-}CH_2]^-$

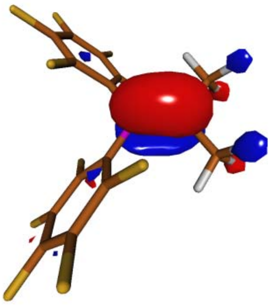

$[(C_6F_5)_2B\text{-}C(CH_3)_2]^-$

Table S3 Energies and thermodynamic corrections for structures discussed in the manuscript, obtained with TPSS-D3/def2-TZVP (+ COSMO corrections).

Compound	E_{vac} [E_h]	E(ε=9.08) [E_h]	E(ε=46.7) [E_h]	G^{298} [kcal mol^{-1}]	ΔG^{298} (CH_2Cl_2)[a] [kcal/mol]	ΔG^{298} (DMSO)[a] [kcal/mol]
**10** (open)	-2600.98872715	-2600.99860810	-2601.00028483	269.049	+9.60	+9.58
**10** (closed)	-2601.00827705	-2601.01809444	-2601.01974475	271.682	0.00	0.00
**11**	-2600.97243208	-2600.99114245	-2600.99451204	267.781	+13.01	+11.93
**12**	-2795.25271320	-2795.26098933	-2795.26235875	330.770	0.0	0.0
**13**	-2795.23986446	-2795.25475355	-2795.25738030	328.758	+1.90	+1.11
**6**	-2870.31808783	-2870.32865245	-2870.33043858	321.632	0.0	0.0
**7**	-2870.30580347	-2870.32615738	-2870.32983094	320.362	+0.30	-0.89
$HB(C_6F_5)_2$	-1481.88713962	-1481.89235577	-1481.89322546	40.396	–	–
**8**	-4352.24726287	-4352.26993735	-4352.27387646	379.793	-12.94	-13.74
**14*15** (complex)	-2717.7773967	-2717.7880430	-2717.789868	301.791	0.00	0.00
E-**16**	-2717.7607589	-2717.7820440	-2717.785910	302.380	4.35	3.07
Z-**16**	-2717.7734048	-2717.7899920	-2717.792990	304.682	1.67	0.93
**17**	-2717.8099041	-2717.8211920	-2717.823106	308.868	-13.72	-13.78

[a]ΔG^{298} relative to the respective reference structure (0.0) of each of the four blocks

Figure S50 Optimized molecular conformations of compounds discussed in the manuscript, obtained with TPSS-D3/def2-TZVP

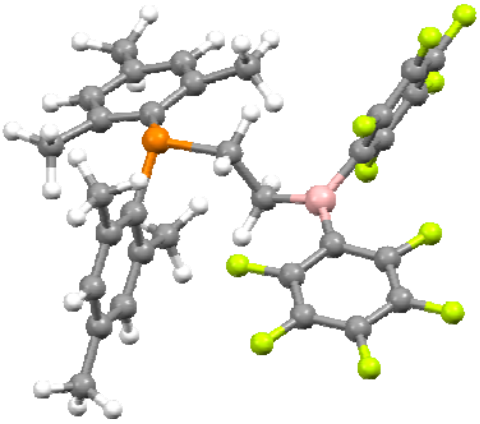

10 (open)

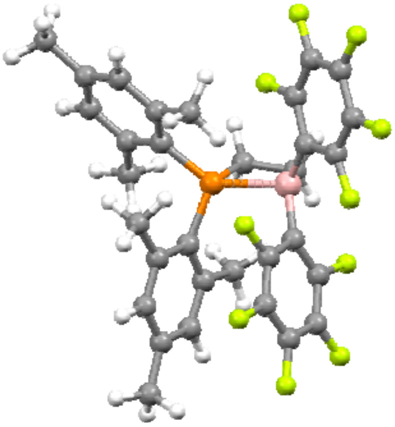

10 (closed)

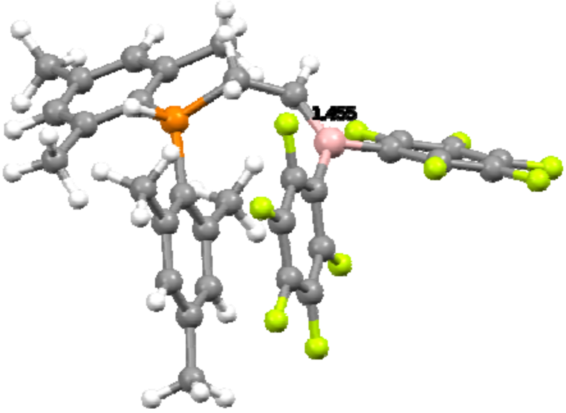

11

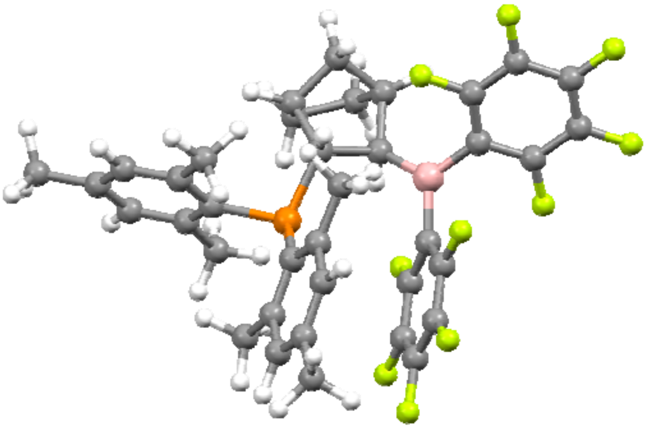

12

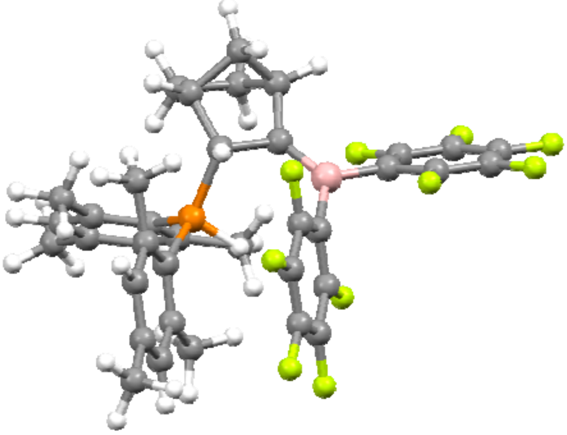

13

Figure S50 (continued)

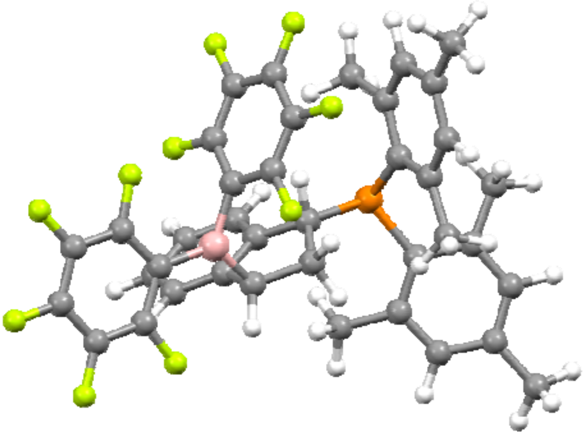

6

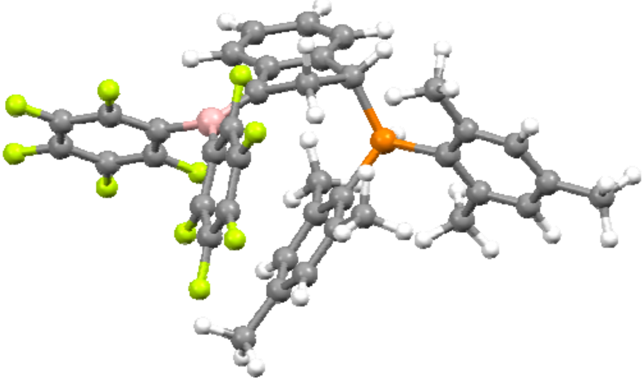

7

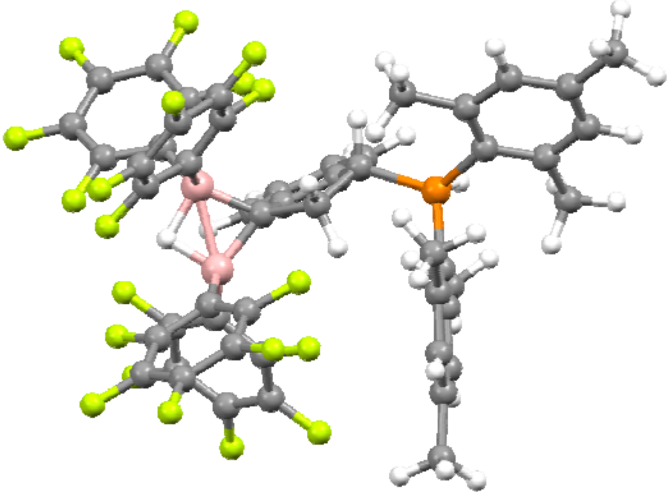

8

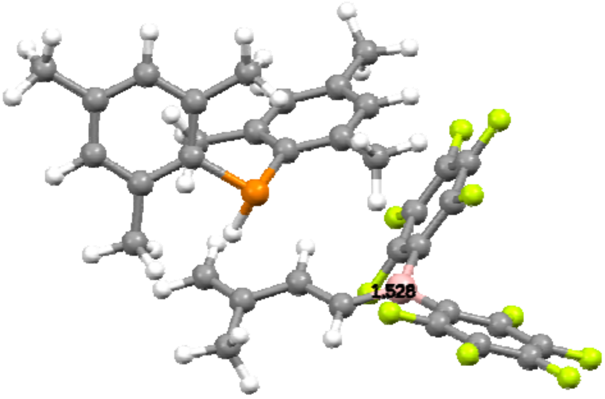

14*15 (complex)

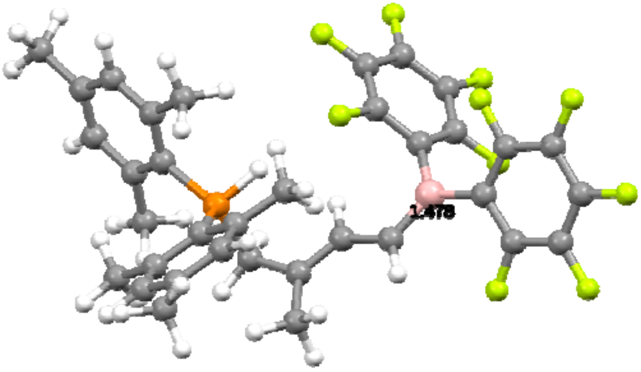

(E)-**16**

Figure S50 (continued)

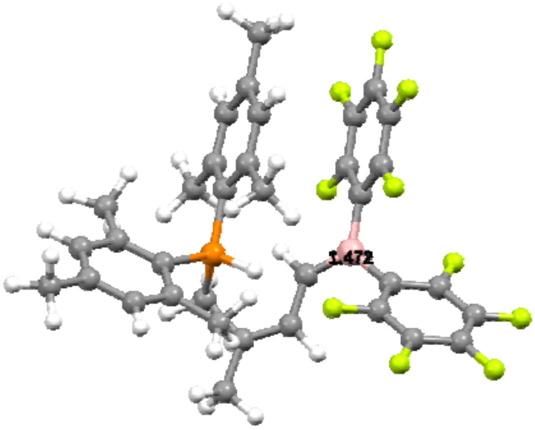

(Z)-**16**

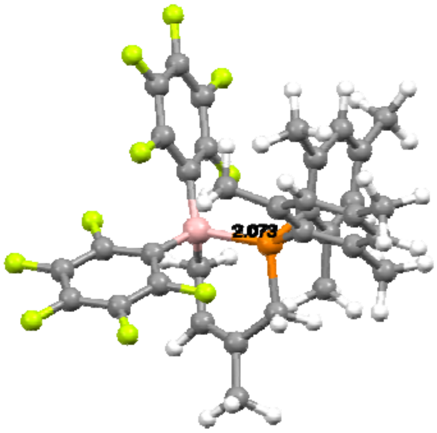

17
